# A *Vitis vinifera* basic helix–loop–helix transcription factor enhances plant cell size, vegetative biomass and reproductive yield

**DOI:** 10.1111/pbi.12898

**Published:** 2018-04-16

**Authors:** Sung Don Lim, Won Choel Yim, Degao Liu, Rongbin Hu, Xiaohan Yang, John C. Cushman

**Affiliations:** ^1^ Department of Biochemistry and Molecular Biology University of Nevada, Reno Reno NV USA; ^2^ Biosciences Division Oak Ridge National Laboratory Oak Ridge TN USA

**Keywords:** basic helix–loop–helix transcription factor, cell expansion, auxin, delayed flowering, biomass production, *Arabidopsis thaliana*

## Abstract

Strategies for improving plant size are critical targets for plant biotechnology to increase vegetative biomass or reproductive yield. To improve biomass production, a codon‐optimized helix–loop–helix transcription factor (*VvCEB1*_*opt*_) from wine grape was overexpressed in *Arabidopsis thaliana* resulting in significantly increased leaf number, leaf and rosette area, fresh weight and dry weight. Cell size, but typically not cell number, was increased in all tissues resulting in increased vegetative biomass and reproductive organ size, number and seed yield. Ionomic analysis of leaves revealed the *VvCEB1*_*opt*_‐overexpressing plants had significantly elevated, K, S and Mo contents relative to control lines. Increased K content likely drives increased osmotic potential within cells leading to greater cellular growth and expansion. To understand the mechanistic basis of *VvCEB1*_*opt*_ action, one transgenic line was genotyped using RNA‐Seq mRNA expression profiling and revealed a novel transcriptional reprogramming network with significant changes in mRNA abundance for genes with functions in delayed flowering, pathogen–defence responses, iron homeostasis, vesicle‐mediated cell wall formation and auxin‐mediated signalling and responses. Direct testing of *VvCEB1*_*opt*_‐overexpressing plants showed that they had significantly elevated auxin content and a significantly increased number of lateral leaf primordia within meristems relative to controls, confirming that cell expansion and organ number proliferation were likely an auxin‐mediated process. *VvCEB1*_*opt*_ overexpression in *Nicotiana sylvestris* also showed larger cells, organ size and biomass demonstrating the potential applicability of this innovative strategy for improving plant biomass and reproductive yield in crops.

## Introduction

Increasing plant biomass and seed yield in plants using biotechnological approaches is an important goal to increase crop productivity. Many genes have been discovered that play roles in altering cell or organ size that have the potential to lead to increased crop yields (Hardin and Wang, 2013; Krizek, 2009; Rojas *et al*., 2010; Vanhaeren *et al*., 2014). Both auxins and brassinosteroids (BRs) stimulate plant organ size by stimulating both cell proliferation and cell expansion. Several reports have implicated various factors that increase cell size or expansion through the action of these phytohormones. Overexpression of the *Arabidopsis* Auxin‐regulated Gene Involved in Organ Size (ARGOS) gene, which is highly induced by auxin, increased aerial organ size mainly through increasing cell proliferation (Hu *et al*., 2003). Similarly, the overexpression of the *Arabidopsis ARGOS‐LIKE* (*ARL*) gene, which is an auxin‐regulated gene involved in controlling organ size, resulted in larger cotyledons and leaves and other lateral organs by regulating both cell proliferation and expansion in *Arabidopsis* (Hu *et al*., 2006) and rice (Wang *et al*., 2009). *ARGOS* and *ARL* orchestrate organ growth and final organ size redundantly with the *ORGAN SIZE RELATED 1* (*OSR1*) gene (Feng *et al*., 2011). *ARGOS* acts upstream of *AINTEGUMENTA* (*ANT*), a member of the *AP2/ERF* transcription factor (TF), which, when overexpressed, resulted in larger aerial organs due to increased cell number, as well as in cell size in certain organs including carpels, petals and stamens (Krizek, 1999; Mizukami and Fischer, 2000). The cytochrome P450 *ROTUNDIFOLIA3* (*ROT3*), which encodes CYP90C1 and participates in BR biosynthesis, resulted in cellular expansion, stimulating cell expansion specifically in the longitudinal direction and increasing leaf length (Kim *et al*., 2005). *SMALL AUXIN UP RNA* (*SAUR*) genes 19–24 positively regulate leaf growth when overexpressed (Spartz *et al*., 2012; Vanhaeren *et al*., 2014). Lastly, overexpression of *EXPANSIN 10* enhanced cell and leaf expansion (Cho and Cosgrove, 2000; Vanhaeren *et al*., 2014).

Overexpression of the *Vitis vinifera* cell elongation bHLH protein (*VvCEB1*) gene, which encodes a basic helix–loop–helix (bHLH) family TF (Pires and Dolan, 2009) expressed specifically during berry expansion phases, plays a role in cell expansion driving berry engustment around véraision (Nicolas *et al*., 2013). Overexpression of this bHLH TF in grape embryos showed that this protein controls cell expansion and effects the expression of several auxin metabolism and auxin signalling genes including AUX/IAA and auxin‐response TFs, other auxin‐responsive genes, genes encoding cell wall modification proteins and enzymes, and aquaporins (Nicolas *et al*., 2013). *VvCEB1* is related to a subfamily of bHLH TFs associated with cell growth and organ size such as *BIGPETALp*, which functions as a negative regulator of cell expansion and petal growth in an auxin‐dependent manner (Szécsi *et al*., 2006; Varaud *et al*., 2011). In contrast, the *UPA20* gene, which encodes a bHLH TF from sweet bell pepper (*Capsicum annuum*), functions as a positive regulator of cell size (Kay *et al*., 2007). Other members of the bHLH gene family function to negatively (e.g. *AtbHLH137*) (Zentella *et al*., 2007) or positively regulate gibberellic acid (GA) signalling (e.g. *BEE1*,* BEE2* and *BEE3*) to alter plant growth and development (Friedrichsen *et al*., 2002). Overexpression of *BEE1* resulted in increased floral organ size, increased sensitivity to BRs and partial insensitivity to abscisic acid (Friedrichsen *et al*., 2002). Another bHLH TF family member, *CRYPTOCHROME‐INTERACTING bHLH (AtCIB1)*, is involved in *CRYTOCHROME 2* (*CRY2*) interaction in a blue light‐specific manner to promote floral initiation by promoting *FLOWERING LOCUS T (FT)* mRNA expression (Liu *et al*., 2008). *AtCIB1* also interacts with *AtCIB5* via heterodimerization and acts redundantly with *AtCIB1* (and possible *AtCIB2* and *AtCIB4*) to activate FT transcription and promote flowering (Liu *et al*., 2013).

Here, overexpression of the *VvCEB1*_*opt*_ gene in *Arabidopsis* resulted in a global increase in plant cell size, vegetative biomass and seed production along with a 2‐week delay in flowering. RNA‐Seq analysis showed that a complex set of mRNA expression changes occurred across genes from functionally diverse pathways including those that orchestrate auxin‐mediated responses that drive cell expansion and proliferation. A significant increase in leaf and root auxin content and proliferation in lateral leaf primordia within meristems were observed in *VvCEB1*_*opt*_ overexpression lines resulting in increased biomass production and seed yield. *VvCEB1*_*opt*_ overexpression in tobacco (*Nicotiana sylvestris*) also resulted in larger cells, organ size and biomass demonstrating the potential applicability of this innovative approach for improving plant biomass and reproductive yield in crops.

## Results and discussion

### VvCEB1_*opt*_ overexpression increases organ size and biomass

**Figure 1:**
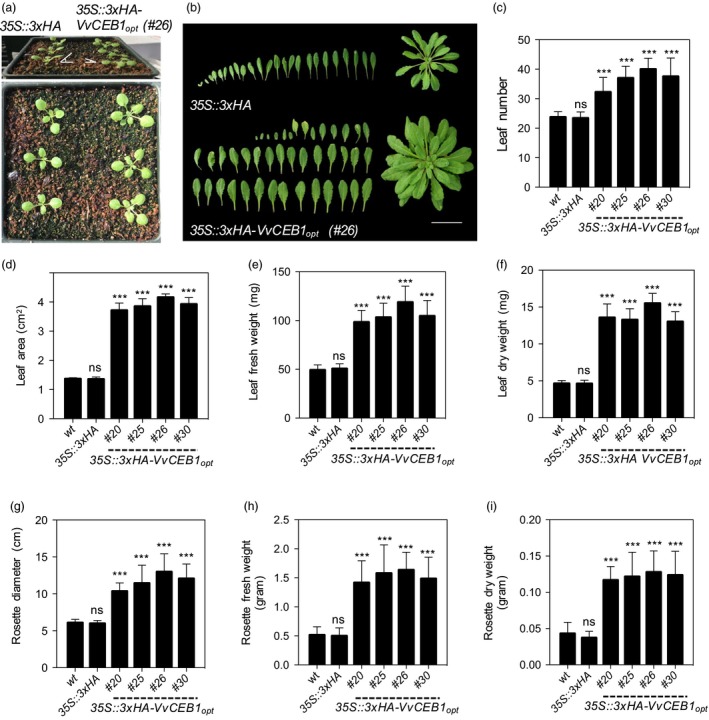
*Vv* CEB1_opt_ overexpression increases biomass in *Arabidopsis*. (a) Seedling images (1‐week‐old) of the *35S::3xHA* empty‐vector control line and *VvCEB1*_*opt*_‐overexpressing line (#26). (b) Leaf and rosette images (4‐week‐old) of the 35S::3xHA empty‐vector line and *VvCEB1*_*opt*_‐overexpressing line (#26). Scale bar, 5 cm. (c) Comparison of leaf number (*n *=* *15). (d) Leaf area of fifth leaf (*n *=* *10). (e) Leaf fresh weight (*n *=* *12). (f) Leaf dry weight (*n *=* *12). (g) Rosette diameter (*n *=* *20). (h) Rosette fresh weight (*n *=* *12). (i) Rosette dry weight (*n *=* *12). Values represent means ± SD, ns = non‐significant, ***P <* *0.001, one‐way anova with Dunnett's multiple comparison test.

Four independent transformants expressing a codon‐optimized version of the *VvCEB1* gene (*VvCEB1*_*opt*_ lines #20, 25, 26 and 30) with an N‐terminal 3xHA tag in *Arabidopsis* under the control of the CaMV 35S promoter were generated (Figure S1). Empty‐vector (EV) control lines expressing only a 3xHA tag were also constructed to serve as controls (Figure S1). C‐terminal *VvCEB1*_*opt*_ *::sGFP* fusions were localized to the nucleus (Figure S1). The relative mRNA and fusion protein expression varied among the four lines, and line *35S::3xHA‐VvCEB1*_*opt*_ #26 had the greatest *VvCEB1*_*opt*_ expression (Figure S2). The *VvCEB1*_*opt*_‐overexpressing lines, as exemplified by line #26, showed a decrease in leaf angle relative to the EV control line (Figure 1a). Such leaf epinasty suggests alterations in auxin metabolism (Sandalio *et al*., 2016). Under soil‐grown conditions, the *VvCEB1*_*opt*_‐overexpressing lines also showed a 1.3‐ to 1.6‐fold increase in leaf number (Figure 1b–c), a 2.7‐ to 3.3‐fold increase in leaf area (Figure 1d), and 1.9‐ to 2.4‐fold and 2.8‐ to 3.3‐fold increases in leaf fresh and dry weight, respectively, relative to the Col‐0 wild‐type and EV control lines (Figure 1e–f). Similarly, rosette diameter increased 1.6‐ to 2.1‐fold (Figure 1g) and rosette fresh and dry weight increased 2.7‐ to 3.1‐fold and 2.7‐ to 2.9‐fold, relative to the control lines, respectively (Figure 1h–i). Plant growth was also monitored for plants grown on agar plates. Although plants showed no significant difference in germination rate on agar plates (Figure S3), the *VvCEB1*_*opt*_‐overexpressing lines showed significantly increased biomass accumulation as measured by either fresh or dry weight of total aerial tissues in plants 1, 2 or 3 weeks of age (Figure S4). Total aerial fresh and dry vegetative biomass increased 1.6‐ to 2.3‐fold and 1.5‐ to 2.9‐fold relative to the wild‐type and EV controls, respectively (Figure S4). These results demonstrate that *VvCEB1*_*opt*_ overexpression promotes aerial growth detectable within 1 week whether the plants are grown in soil under real‐world conditions or on agar plates.

Close examination of leaf morphology revealed a significant increase in leaf teeth number and height within multiple leaves in *VvCEB1*_*opt*_‐overexpressing lines compared to control lines (Figure S5). Such enhanced leaf margin serrations are known to arise from spatially distributed peaks in auxin modulated by *PIN‐FORMED 1* (*PIN1*), a polar auxin efflux carrier, and the growth repressor *CUP‐SHAPED COTYLEDON 2* (*CUC2*) (Bilsborough *et al*., 2011). Thus, this observation suggests that auxin content, transport and signalling are altered in the *VvCEB1*_*opt*_‐overexpressing lines.

**Figure 2:**
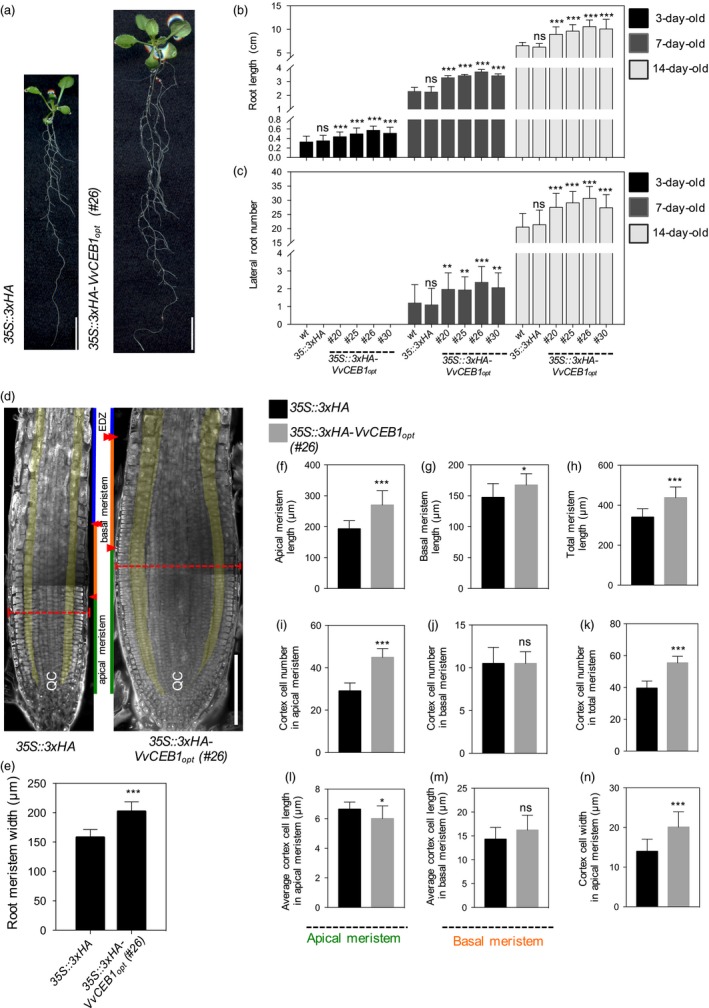
*Vv* CEB1_opt_ overexpression increases root biomass in *Arabidopsis*. (a) Root images of *35S::3xHA* empty‐vector control line and *VvCEB1*_*opt*_‐overexpressing line (#26). Scale bar, 1 cm. (b) Comparison of primary root length (*n *=* *30). (c) Lateral root number (*n *=* *30). (d) Images of root meristems of *35S::3xHA* empty‐vector control line and *VvCEB1*_*opt*_‐overexpressing line (#26). Orange broken lines indicate root meristem widths. Green, orange and blue lines represent the lengths of the apical meristem, the basal meristem and the elongation/differentiation zone, respectively. Single and double arrowheads indicate the borders of the apical meristem and basal meristem, respectively (Hacham *et al*., 2011). The cortex cell layers are pseudo‐coloured in yellow. QC indicates the quiescent centre. Scale bar, 150 μm. (e) Comparison of root meristem width (*n *=* *16). Quantification of (f) apical meristem length, (g) basal meristem length and (h) total meristem length (*n *=* *12). Quantification of (i) cortical cell number in apical meristem zone, (j) cortical cell number in basal meristem zone and (k) total cortical cell number (*n *=* *12). Average cortical cell number in (l) apical meristem zone and (m) basal meristem zone (*n *=* *12). (n) Cortical cell width in apical meristem zone (*n *=* *120). Values represent means ± SD, ns = non‐significant, *P <* *0.05, **P <* *0.01 and ***P <* *0.001, one‐way anova with Dunnett's multiple comparison test (b and c) and Student's *t*‐test (e–n).

In addition to aerial growth, the effects of *VvCEB1*_*opt*_ overexpression in hypocotyls and roots of *Arabidopsis* were also examined. Hypocotyl length decreased 0.7‐ to 0.8‐fold, but width increased 1.4‐ to 1.7‐fold, respectively, compared to control lines (Figure S6). Under agar plate‐grown conditions, the four *VvCEB1*_*opt*_‐overexpressing lines showed a 1.3‐ to 1.7‐fold increase in root length (Figure 2a,b) and a 1.3‐ to 1.9‐fold increase in lateral root number (Figure 2c) in 3‐, 7‐ and 14‐day‐old plants, respectively, relative to control lines. Total fresh and dry root biomass increased 1.6‐ to 1.8‐fold and 1.6‐ to 2.0‐fold, respectively, relative to control lines (Figure S7). Root meristems were significantly wider in the *VvCEB1*_*opt*_‐overexpressing line #26 relative to the EV control when measured at their widest point (Figure 2d,e). This increase in root thickness was due in part to an increase in the number of cell layers (Figure 2d). Furthermore, the length of the apical, basal and total root meristem was 1.1‐ to 1.3‐fold greater than that in the EV control line (Figure 2f–h). The root meristem zone can be divided into the apical and basal meristem zones (Hacham *et al*., 2011; Ishikawa and Evans, 1995; Verbelen *et al*., 2006). The apical meristem is characterized by a high rate of cell division and proliferation. The basal meristem is also referred to as the transition zone between the apical meristem and the elongation zone, where cells exhibit slower proliferation rates or complete cessation of cell division and cells become larger due to elongation. These increases in length were due in part to an increase in cell number within the cortex of the apical meristem (Figure 2i–k). The width of the cortical cells within the apical meristem was also 1.4‐fold greater than that in the EV control line, but the length of these cells was shorter (Figure 2l–n). Individual root cell size of all root cell types (e.g. epidermis, cortex and endodermis) in the mature root zone was also increased significantly, relative to control lines (Figure S8). These results show that *VvCEB1*_*opt*_ overexpression increased overall root size with an increase in meristem zone cell number and size, and lateral root number.

### VvCEB1_opt_ overexpression increases leaf cell size, chloroplast number and protein content

**Figure 3:**
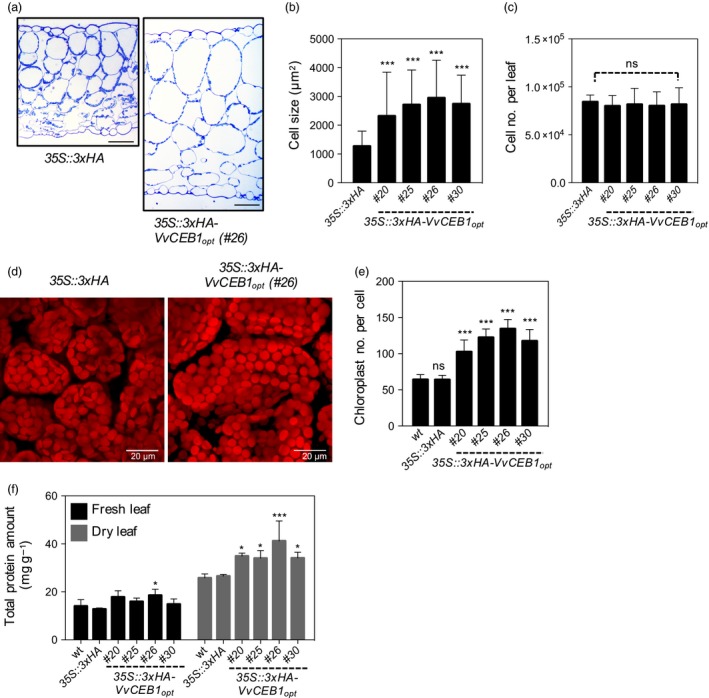
*Vv* CEB1_opt_ overexpression increases cell size in *Arabidopsis*. (a) Transverse sections of leaves from the *35S::3xHA* empty‐vector control line and *VvCEB1*_*opt*_‐overexpressing line (#26). Scale bar, 100 μm. (b) Palisade mesophyll cell size (*n *=* *110). (c) Mesophyll cell number per fifth fully expanded leaf (*n *=* *10). (d) Images of palisade mesophyll cells and chloroplasts of *35S::3xHA* empty‐vector control line and *Vv* CEB1_opt_‐overexpressing line (#26). Scale bar, 20 μm. (e) Chloroplast number per palisade mesophyll cell (*n *=* *30). (f) Total protein amount of fifth leaves on a fresh or dry weight basis (*n *=* *4). Values represent means ± SD, ns = non‐significant, *P <* *0.05, **P <* *0.01 and ***P <* *0.001, one‐way anova with Dunnett's multiple comparison test.

To determine whether increased leaf size was due to increased cell number or cell size, cell size was measured in transverse leaf sections. Cell size in leaves increased by 1.3‐ to 1.7‐fold in all four *VvCEB1*_*opt*_‐overexpressing lines relative to controls (Figures 3a,b and S9a). Palisade mesophyll cell size (as measured by cell area) increased by 1.8‐ to 2.3‐fold in the *VvCEB1*_*opt*_‐overexpressing lines compared with the EV control line (Figure 3a,b). Spongy mesophyll cell size increased by 2.0‐ to 2.5‐fold in the *VvCEB1*_*opt*_‐overexpressing lines (Figure S9b). Leaf epidermal pavement cells also exhibited significant size increases on both the adaxial and abaxial leaf surfaces (Figure S9c–e). However, overall cell number per leaf showed no significant increase (Figure 3c), suggesting that the increased aerial organ size of the *VvCEB1*_*opt*_‐overexpressing lines was the result of increased cell size. The only exception was found in root meristems where both cortical cell size and number were increased (Figure 2d,l–n). Thus, the increased cortical cell size, root length and width, and greater number of lateral roots likely contribute to the improved growth and reproductive capacity of the *VvCEB1*_*opt*_‐overexpressing lines relative to control lines (Figure 6).

Despite the larger cell size, no increase in DNA content per cell was observed in either leaves or roots (Figure S9f), suggesting that the increase in cell size was not associated with an increase in ploidy level of the cells. Increased somatic endopolyploidy has been described in desert succulents with large cells (De Rocher *et al*., 1990); however, in *Arabidopsis*, increased cell size was not necessarily correlated with increased endopolyploidy (Tsukaya, 2013).

The increased cell size also resulted in a 1.6‐ to 2.0‐fold increase in chloroplast number per cell in the palisade cells of the *VvCEB1*_*opt*_‐overexpressing lines compared with control lines (Figure 3d,e). This increase in chloroplast number was reflected in an increase in chlorophyll content per leaf or plant, but not on a fresh weight basis due to the larger size and greater number of leaves in the *VvCEB1*_*opt*_‐overexpressing lines (Figure S10). The *VvCEB1*_*opt*_‐overexpressing lines also exhibited up to a 1.5‐fold increase in leaf protein content on a dry weight basis (Figure 3f). This increase was likely due in part to the observed increases in chloroplast number per cell. The increases in chlorophyll and protein contents corresponded well with a significant increase in light‐harvesting chlorophyll–protein complex II subunit B1 (*LHB1B1*, At2g34430) mRNA expression in leaves and flowers of *VvCEB1*_*opt*_‐overexpressing lines #26 (Tables S2 and S3). Interestingly, increased cell size was also associated with a significant decrease in cell wall thickness in a majority of *VvCEB1*_*opt*_‐overexpressing lines (Figure S11). These results suggest that increased cellular expansion might be coupled with thinner cell walls, but no gross detrimental effects on plant structural integrity were apparent in these lines other than the reduced leaf angle observed in young plants (Figure 1a).

### VvCEB1_opt_ overexpression alters the ionome

Plant cell growth and expansion are driven by the ability of the plant to maintain cellular osmotic adjustment and turgor, which drive cellular growth and expansion, and regulate stomatal function (Barragán *et al*., 2012). Thus, the possibility that increased cellular expansion might be driven by alterations in the ionic composition of the cells was examined by conducting detailed ionomic analysis. While most of the 27 inorganic ions surveyed showed no significant changes, a significant decrease in total leaf Ca and P contents was observed in a majority of lines relative to the control lines (Figure S12). The decrease in Ca might be related to the observed decrease in cell wall mass as the cell wall is known to be a major storage site for Ca within plants (Kader and Lindberg, 2010). In contrast, significant increases in total leaf K, S and Mo contents were observed (Figure S12). The observed increase in total leaf K, which drives osmotic potential within cells to promote cellular growth and expansion in an auxin‐dependent manner (Claussen *et al*., 1997), is consistent with the observed increases in cell size. Elevated Mo content of leaves might be related to the improved growth rates associated with increased nitrate reductase and xanthine dehydrogenase activity (Ventura *et al*., 2010). Increased aldehyde oxidase (AO) activity, a molybdenum cofactor‐containing enzyme and *ATAO1* mRNA expression has been observed in the indole‐3‐acetic acid (IAA)‐overproducing mutant *sur1*, suggesting that this AO activity catalyses the conversion of indole‐3‐acetaldehyde (IAAld) to IAA (Sekimoto *et al*., 1998; Seo *et al*., 1998). Such activity would be consistent with the observed increases in auxin content within *VvCEB1*_*opt*_‐overexpressing lines (Figure 7b,c).

### VvCEB1_opt_ overexpression increases reproductive capacity

**Figure 4:**
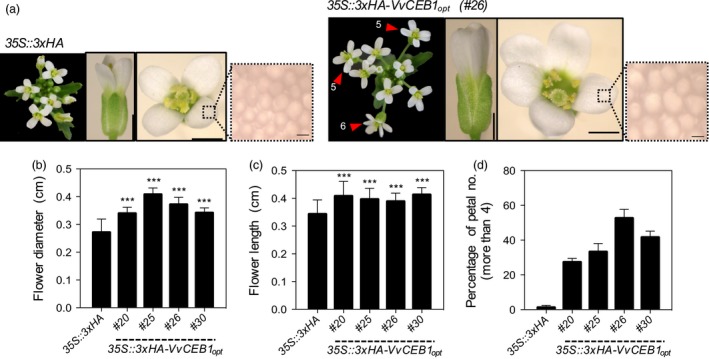
*Vv* CEB1_opt_ overexpression increases flower size and number of petals and sepals in *Arabidopsis*. (a) Representative images of the inflorescence apex, flower and petal epidermis cell (left to right) of the *35S::3xHA* empty‐vector control and *VvCEB1*_*opt*_‐overexpressing lines (#26). Magnified images represent petal epidermal cells. Numbers indicate number of flower petals >4. Scale bars indicate 0.1, 0.1 cm and 10 μm (left to right), respectively. (b) Flower diameter was measured top view petal tip‐to‐petal tip (*n *=* *63). (c) Flower length was measured side view (*n *=* *63). (d) Percentage of flowers with petal numbers >4 (*n *=* *3). Values represent means ± SD, ***P <* *0.001, one‐way anova with Dunnett's multiple comparison test.

The effects of *VvCEB1*_*opt*_ overexpression were evaluated on the reproductive capacity of *Arabidopsis*. Sepal number was greater in *VvCEB1*_*opt*_‐overexpressing lines than in the control line (Figure S13a,b). Flowers of *VvCEB1*_*opt*_‐overexpressing lines were also 1.2‐ to 1.5‐fold larger in diameter (top view measured petal to petal), 1.2‐fold longer (side view) and 1.3‐ to 1.5‐fold wider (side view) than the EV control line (Figures 4a–c and S13c). Flowers in the *VvCEB1*_*opt*_‐overexpressing lines also had a greater number of petals per flower (Figure 4d). Primary inflorescence stems were also 1.2‐ to 1.7‐fold greater in diameter in the *VvCEB1*_*opt*_‐overexpressing lines with larger vascular bundles, an indicator of increased auxin activity (Figure S14a–c).

**Figure 5:**
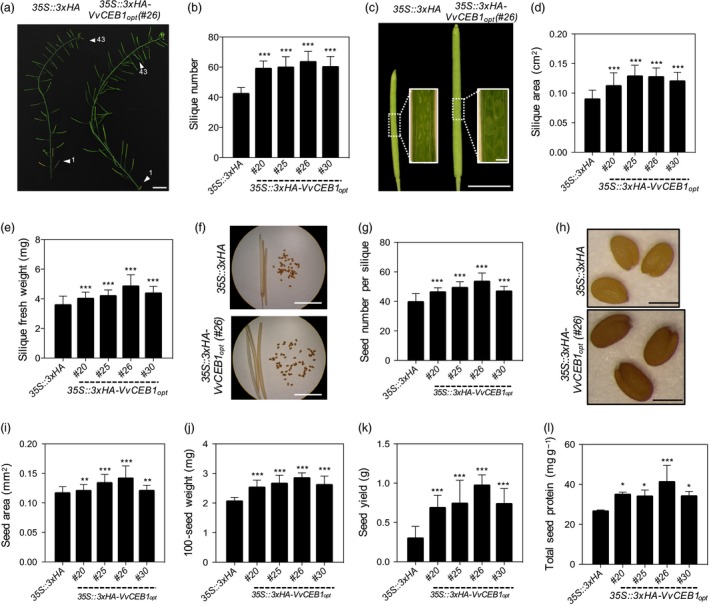
VvCEB1_opt_ overexpression increases size of reproductive structures and seed yield in *Arabidopsis*. (a) Representative images of primary inflorescence stem of the *35S::3xHA* empty‐vector control line and the *VvCEB1*_*opt*_‐overexpressing line (#26). Arrowheads indicate 1st and 43rd silique from rosette leaf. Scale bar, 1.5 cm. (b) Silique number within primary inflorescence (*n *=* *20). (c) Representative images of fully developed siliques of the *35S::3xHA* empty‐vector control line and *VvCEB1*_*opt*_‐overexpressing line (#26). Scale bar, 0.5 cm. Scale bar in inset images, 0.5 mm. (d) Silique area (*n *=* *50). (e) Silique fresh weight (*n *=* *40). (f) Representative images of seed number per dried silique of the *35S::3xHA* empty‐vector control line and *VvCEB1*_*opt*_‐overexpressing line (#26). Scale bar, 0.5 cm. (g) Seed number per silique (*n *=* *30). (h) Representative seed images of the *35S::3xHA* empty‐vector control line and *VvCEB1*_*opt*_‐overexpressing line (#26). Scale bar, 0.5 mm. (i) Seed area (*n *=* *100). (j) 100‐seed weight (*n *=* *30). (k) Seed yield per plant (*n *=* *10). (i) Total seed protein (*n *=* *4). Values represent means ± SD, *P <* *0.05, **P <* *0.01 and ***P <* *0.001, one‐way anova with Dunnett's multiple comparison test.

In addition to increased flower size and floral organs per flower, the number of flowers and thus siliques, per inflorescence was 1.3‐ to 1.4‐fold greater in the *VvCEB1*_*opt*_‐overexpressing lines than in the EV control line (Figure 5a,b). Silique area increased 1.2‐ to 1.4‐fold (Figure 5c,d), and silique fresh weight increased 1.1‐ to 1.3‐fold (Figure 5e) in the *VvCEB1*_*opt*_‐overexpressing lines. Seed number per silique was also 1.1‐ to 1.3‐fold greater in the *VvCEB1*_*opt*_‐overexpressing lines (Figure 5f,g). Seed size increased 1.1‐ to 1.2‐fold as measured by seed area, and 100‐seed weight increased by 1.2‐ to 1.4‐fold (Figure 5h–j). Total seed yield increased by an average of 2.3‐ to 3.2‐fold in the *VvCEB1*_*opt*_‐overexpressing lines (Figure 5k). Accompanying the increased seed size was a significant increase in total seed protein (Figure 5l). Interestingly, the *VvCEB1*_*opt*_‐overexpressing lines showed a 2‐week delay in floral development (bolting) whether grown in soil or on agar plates under 12‐h day and a 1‐week delay under 16‐h day conditions (Figure S15a–d).

The greater reproductive capacity of the *VvCEB1*_*opt*_‐overexpressing lines might arise from improved nutrient and water uptake due to larger root size and branching and greater source capacity due to increased leaf and rosette size. To assess the leaf source capacity, starch and soluble sugar contents of leaves were measured. The *VvCEB1*_*opt*_‐overexpressing lines showed significantly lower soluble sugar and starch contents than control lines when measured on a per tissue fresh weight or a per leaf area basis (Figure S16a–c,f,g). However, the *VvCEB1*_*opt*_‐overexpressing lines showed a 1.5‐ to 1.6‐fold and a 2.4‐ to 2.9‐fold increase in soluble sugar content when expressed on a per leaf or a per plant basis, respectively (Figure S16d,e), but showed no significant difference in starch content when expressed on a per leaf or a per plant basis, respectively (Figure S16h,i). These results resemble those observed by the overexpression of the *UPA20* gene from sweet bell pepper (*C. annuum*), which caused a decrease in starch content in the resulting hypertrophic cells (Kay *et al*., 2007). Interestingly, increased soluble sugars are known to increase IAA biosynthesis (Sairanen *et al*., 2012). Thus, the observed increases in soluble sugars might be linked to the observed increases in auxin accumulation in *VvCEB1*_*opt*_‐overexpressing lines (Figure 7b,c) and to the enhanced expression of *SUCROSE SYNTHASE 3* (*SUS3*, At4g02280) in all tissues of the *VvCEB1*_*opt*_‐overexpressing lines #26, but was most significantly induced in the inflorescences (Table S2).

### Increased cell and organ size involves complex transcriptional reprogramming

**Figure 6:**
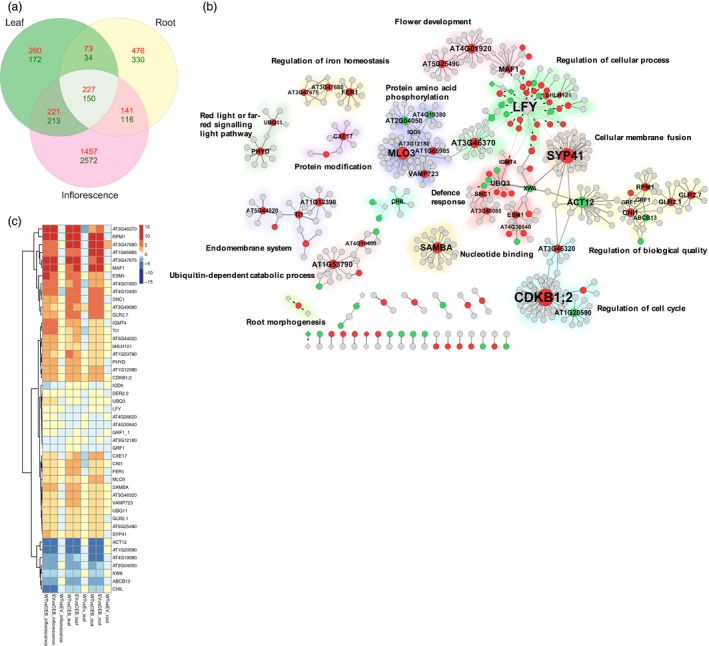
Functional network analysis of the mRNA gene expression in VvCEB1_opt_‐overexpressing lines compared with control lines. (a) Three‐way Venn diagram analysis of RNA‐Seq reads revealed distinct expression patterns between leaves, roots and inflorescence tissues. Numbers of genes that are differentially expressed between the Col‐0 wild‐type and *35S::3xHA* empty‐vector control lines and the *VvCEB1*_*opt*_‐overexpressing line (#26) as determined by two or more methods (e.g. DESeq2, edgeR, ROTS and voom) with ≥twofold increase (red) or decrease (green) in relative transcript abundances and a false discovery rate (FDR) of less than 0.001. (b) Protein–protein and transcriptional regulatory network of differentially expressed genes shared among leaves, roots and inflorescences as determined by edgeR. Gene nodes with increased, decreased or unchanged mRNA expression are indicated by red, green or grey circles, respectively. The node size indicates the degree of connectivity of nodes. Nodes indicated by diamonds represent TFs. The edges shown in solid or dotted lines represent protein–protein or regulatory interactions, respectively. Arrows indicate activation, and short bars represent repression. Network modules enriched for gene ontology biological process terms are highlighted with different colours. (c) Heat map of hierarchical clustering analysis of networked genes with high connectivity (≥4) based on their topology coefficients representing similarities in gene expression profiles across all organ types as determined by edgeR. The colour scale indicates log_2_‐fold changes in mRNA abundance.

To investigate the transcriptional programme driving the increased biomass and reproductive capacity due to *VvCEB1*_*opt*_ overexpression, Illumina‐based RNA‐Seq was performed to profile mRNA expression in leaves, roots and primary inflorescences (Figures S18–S23). A set of 227 and 150 genes was identified whose mRNAs showed significantly increased or decreased transcript abundance, respectively, in leaves, roots and inflorescences in the *VvCEB1*_*opt*_‐overexpressing line #26 relative to both the wild‐type and EV control lines using the consensus of four different RNA‐Seq analysis programmes (e.g. DESeq2, edgeR, ROTS and voom) (Figure 6a and Table S1). Inflorescences showed the greatest diversity of transcripts due to the complexity of different cell types within this organ, followed by roots and then leaves. The consensus set of 227 genes with increased transcript abundance showed enrichment for gene ontology (GO) terms involved in several biological processes. Analysis using ThaleMine at the Araport database (Krishnakumar *et al*., 2015) revealed significant enrichment of genes involved in a wide range of functions including defence response, ADP binding and *NB‐ARC* (nucleotide‐binding adaptor shared by *APAF‐1*, R proteins and *CED‐4*) domains from resistance proteins involved in plant innate immunity (Figure S24). The mRNA expression profiles unique to each organ type are reported (Figures S19–21 and Tables S2–S4). Overall, this analysis revealed a complex assortment of mRNA expression changes representing a diverse array of cellular functions.

### Network analysis reveals complex functional associations

**Figure 7:**
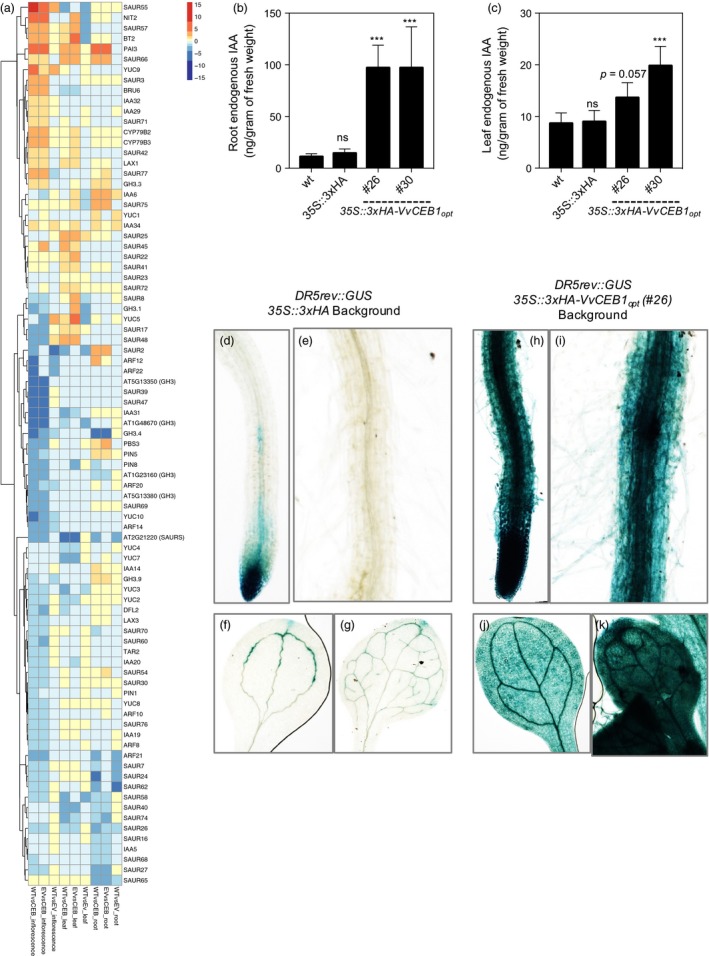
*Vv* CEB1_opt_ overexpression increased auxin accumulation in *Arabidopsis*. (a) Heat map of hierarchical clustering analysis of manually curated genes with functions related to auxin biosynthesis, perception, transport and response. Indole‐3‐acetic acid (IAA) content in (b) roots and (c) leaves of wild‐type (wt), *35S::3xHA* empty‐vector (EV) and *35S::VvCEB1*_opt_ lines (*n *=* *5). Values represent means ± SD, ns = non‐significant, ***P < 0.01, one‐way anova with Dunnett's multiple comparison test. Expression of *DR5rev::GUS* in *35S::3xHA* empty‐vector (EV) line: (d) root tip, (e) mature root region and root hairs, (f) cotyledon and (g) 1st leaf of EV control plant. Expression of *DR5rev::GUS* in *Ox‐VvCEB1*_*opt*_ line (#26): (h) root tip, (i) mature root region and root hairs, (j) cotyledon and (k) 1st leaf of 10‐day‐old seedlings.

Network analysis of known protein–protein interactions (PPIs) and regulatory relationships was conducted to explore the potential functional associations among the shared set of 377 differentially expressed genes common to leaves, roots and inflorescences (Figure 6a). The network comprised of 479 genes (82 and 32 genes with increased and decreased relative mRNA abundance, respectively) connected by 484 protein–protein and regulatory interactions (Figure 6b and Table S5). The relative differential expression of hub genes as estimated by edgeR with greater than four connections within the network is shown as a hierarchical clustering heat map for comparison of each organ in the *VvCEB1*_*opt*_‐overexpressing line #26 relative to both the wild‐type and EV control lines (Figure 6c). Network hubs that exhibited increased mRNA expression patterns in the *Vv*CEB1_opt_‐overexpressing line #26 included genes involved in flower timing, plant cell defence responses associated with vesicle‐mediated membrane transport system components involved in cell wall growth and root hair formation and salicylic acid‐dependent defence responses, iron homeostasis and multiple members of the auxin‐responsive protein family of genes associated with cell proliferation and expansion (see also Figure 7a). The gene networks unique to each organ type are also reported (Figures S25–S27) along with the differential expression of selected hub genes with large numbers of connections (Tables S6–S8).

The network analysis revealed a regulatory hub containing the MAF1, a MADS‐box TF, which, when overexpressed, results in late‐flowering plants (Scortecci *et al*., 2001). MAF1 expression was elevated in the *VvCEB1*_*opt*_‐overexpressing line #26, which is consistent with the delayed flowering phenotype in all *VvCEB1*_*opt*_‐overexpressing lines (Figure S15). Other late‐flowering genes were also highly expressed in the *VvCEB1*_*opt*_‐overexpressing inflorescence and leaves, including *CONSTANS‐like 9* (*CO9*), which delays flowering when overexpressed (Cheng and Wang, 2005), and *FLAVIN‐BINDING KELCH REPEAT F‐BOX1* (*FKF1*), which represses flowering (Takase *et al*., 2011) (Tables S2–S3). In contrast, the transcript abundance of *LEAFY* (*LFY*) decreased in leaves of *VvCEB1*_*opt*_‐overexpressing line #26 (Figure 6b,c), which is consistent with its role in promoting the transition from vegetative to reproductive growth (Blázquez *et al*., 1997) and the observed delay in flowering (Figure S14). *LFY* activates the expression of a leucine‐rich repeat protein kinase family protein (AT3G46370), which serves as a major hub, and displayed increased mRNA abundance (Figure 6b,c). Within this subnetwork, a *bHLH121* TF, which displayed increased transcript abundance (Figure 6b,c and Table S1), was also identified. In *Arabidopsis*,* bHLH121* activates the mRNA expression of *HAK5*, a high‐affinity K^+^ transporter, in response to K^+^ deprivation (Hong *et al*., 2013). HAK5 (At4g13420) was identified, but its relative transcript abundance was not found to be significantly induced in any organ. However, a putative potassium transporter (At3g56290) was identified with significantly increased mRNA expression in roots (Table S4). Increased K^+^ uptake is consistent with the observed increases in leaf K content (Figure S12) and would be predicted to drive cellular expansion within the *VvCEB1*_*opt*_‐overexpressing lines. A related but uncharacterized bHLH TF (AT3G56770) also showed increased mRNA expression in all tissues (Table S1) and is an excellent candidate for future functional analysis.

A major hub protein within the protein amino acid phosphorylation subnetwork was the *MILDEW RESISTANCE LOCUS O 3* (*MLO3*) protein, which displayed enhanced mRNA abundance in the *VvCEB1*_*opt*_‐overexpressing line #26 relative to control lines (Figure 6b,c). MLO3 encodes a member of the plasma membrane‐localized family of the plant‐specific seven‐transmembrane domain proteins, which are involved in cell defence responses and induced by fungal/bacterial pathogens and osmotic stress (Chen *et al*., 2006). *MLO3* interacts with a transmembrane protein of unknown function (AT1G65985) and vesicle‐associated membrane protein 723 (*VAMP723*), which is presumably involved in vesicle‐mediated membrane transport. Five other vesicle‐associated membrane proteins associated with membrane fusion events were identified within the network (Table S1), including hub protein *SYNTAXIN OF PLANTS 41* (*SYP41*), which encodes a t‐SNARE protein that relies on *VTI13* (AT3G29100), and a VTI‐type v‐SNARE (vesicle Soluble NSF Attachment protein REceptor) associated with vacuolar trafficking for cell wall growth and root hair formation (Larson *et al*., 2014). The enhanced expression of multiple components of the vesicle‐mediated membrane transport system suggests that the larger cell sizes might require additional material for cell wall formation, larger overall organ sizes and enhanced lateral root formation.

The *SAMBA* gene, which encodes a valine tRNA ligase and is a plant‐specific negative regulator of the anaphase‐promoting complex/cyclosome (APC/C) involved in the degradation of A‐type cyclins, was identified as a hub for the nucleotide‐binding subcomplex (Figure 6b,c). Loss‐of‐function *samba* mutants have enlarged organ size due to the stimulation of cell proliferation in developing seeds early in seedling development (Eloy *et al*., 2012; Vanhaeren *et al*., 2014). However, in *VvCEB1*_*opt*_‐overexpressing line #26, *SAMBA* mRNA expression was increased relative to controls, which might be related in some way to a lack of cell proliferation in most organs except for root cortical meristems (Figure 2i,k). The *BTB AND TSZ DOMAIN PROTEIN 2* (*BT2*), which modulates silique size and seed set (Robert *et al*., 2009), also showed increased mRNA expression, consistent with the increased size of these organs (Figure 5c,d,h–j) and increased seed production and total seed protein (Figure 5k,l) in the *VvCEB1*_*opt*_‐overexpressing lines.

An enriched subnetwork for defence response was also observed (Figure 6b,c), which were comprised of several small hubs that included genes associated with salicylic acid‐dependent defence responses, such as the *SUPPRESSOR OF NPR1‐1*,* CONSTITUTIVE 1* (*SNC1*) gene, which encodes a Toll interleukin‐1 receptor–nucleotide‐binding–leucine‐rich repeat‐type resistance gene (*TIR‐NB‐LRR‐type*) (Xu *et al*., 2014). Another hub in the cell defence response subnetwork, the myrosinase‐associated protein *EPITHIOSPECIFIER MODIFIER 1* (*ESM1*), controls the *ESM1* quantitative trait loci (QTL) involved in hydrolysis of glucosinolate into isothiocyanate leading to insect resistance (Zhang *et al*., 2006b). The very strong induction of RPM1 mRNA expression, which encodes an NBS‐LRR protein that confers resistance to *Pseudomonas syringae* (Russell *et al*., 2015), is an example of the enrichment of proteins capable of recognizing pathogen‐derived effector molecules. Also related to defence‐response functions was the strong induction of *PHYTOALEXIN DEFICIENT 3* (*PAD3*, AT3G26830) in all tissues, but was most significantly induced in the inflorescences (Table S2), and a cytochrome p450 enzyme (CYP71B15), which catalyses the conversion of IAA‐derived dihydrocamalixic acid to camalexin, a powerful phytoalexin conferring innate immunity to insects (Prince *et al*., 2014). While one might expect that a strong induction of plant defences might result in growth inhibition (Campos *et al*., 2016), this was not observed in the *VvCEB1*_*opt*_‐overexpressing line, probably because wide‐scale, jasmonate‐mediated defence responses were not observed as confirmed by mRNA expression profiling.

Network hub genes involved in transcriptional regulation included the target gene FERRITIN 1 (*FER1*), an iron‐storing protein involved in iron homeostasis whose expression is induced by Fe and P starvation, oxidative stress, light and the circadian clock (Bournier *et al*., 2013; Reyt *et al*., 2015). The increased expression of this gene (Figure 6c), as well as *FER3*, might reflect an increased need for *FER1* due to the doubling of chloroplast numbers per cell (Figure 3d,e) or in response to the decrease in P content (Figure S12) in the *VvCEB1*_*opt*_‐overexpressing line #26 compared with control lines. Other genes involved in iron uptake are also present within this cellular process subnetwork, such as FER‐like regulator of iron uptake (*FRU* or *FIT*), a bHLH TF that regulates iron acquisition (Bauer *et al*., 2007).

### Auxin biosynthesis and response network genes

The observed alterations in leaf morphology, increased cell and organ size, and cell number in the root cortex all suggested that auxin biosynthesis, accumulation, transport and signalling were altered in the *VvCEB1*_*opt*_‐overexpressing line. Many auxin biosynthesis genes showed increased in abundance in one or more tissues examined (Figure 7a and Table S1). For example, mRNA for *PHOSPHORIBOSYLANTHRANILATE ISOMERASE 3* (*PAI3*), which encodes the third step in tryptophan biosynthesis leading to auxin biosynthesis (Tao *et al*., 2008), was very strongly induced in all tissues of the *VvCEB1*_*opt*_‐overexpressing line (#26). The mRNA abundances of several genes involved in IAA biosynthesis were increased in all tissues of the *VvCEB1*_*opt*_‐overexpressing line (#26) (Figure 7a and Table S2). These enzymes included *AMI1*, which encodes an enzyme that converts indole‐3‐acetamide to IAA; *CYP79B2* and *CYP79B3*, which encode cytochrome P450 monooxygenases that catalyse the conversion of L‐tryptophan (L‐Trp) to indole‐3‐acetaldoxime (IAOx), an IAA precursor; and *NITRILASE 2* (*NIT2*), which is suggested to convert indole‐3‐acetonitrile (IAN) to IAA (Ljung, 2013). In contrast, *TRYPTOPHAN AMINOTRANFERASE‐RELATED 2* (*TAR2*), which encodes an enzyme that converts L‐Trp to indole‐3‐pyruvic acid (IPA), showed reduced mRNA abundance in flowers. *YUC (YUCCA)* genes, which encode a family of 11 flavin‐containing monooxygenases in *Arabidopsis*, play central roles in auxin biosynthesis through the conversion of IPA to IAA (Dai *et al*., 2013) and in various aspects of plant development (Cheng *et al*., 2006, 2007). The mRNA abundance of most *YUC* genes decreased with the exception of *YUC5*, which showed significantly increased mRNA expression in leaves (Figure 7a and Table S7). Lastly, *SUPERROOT 1 (SUR1)* and *SUR2* (*CYP83B1*), which catalyse the formation of indole‐3‐acetonitrile, a putative IAA precursor synthesized from IAOx (Ljung, 2013), showed non‐significant decreases and increases in mRNA abundance, respectively. Overall, these results suggest a global increase in the biosynthesis of auxin (and possibly auxin conjugates) in the *VvCEB1*_*opt*_‐overexpressing line (#26) relative to the control line, which was confirmed experimentally (Figure 7b–k).

Auxin is perceived by the *TRANSPORT INHIBITOR RESPONSE1/AUXIN‐SIGNALING F‐BOX PROTEINS* (*TIR/AFBs*) coreceptor complex and a member of the *AUXIN/INDOLE ACETIC ACID* (*AUX/IAA*) gene family (Wang and Estelle, 2014). The *TIR/AFB* proteins form subunits of the SKP1‐Cul1‐F‐box (*SCF*)‐type E3 ligase called *SCF* ^*TIR1/AFB*^. *TIR/AFB* proteins did not show significant changes in mRNA expression in the *VvCEB1*_*opt*_‐overexpressing line. The *AUX/IAA* gene family is comprised of 29 genes in *Arabidopsis* (Paponov *et al*., 2008; Remington *et al*., 2004). *AUX/IAA* transcriptional repressor proteins are part of the auxin coreceptors that are targeted for 26S proteasome‐mediated degradation following auxin binding to release auxin‐response factors (ARFs), leading to the transcription of auxin‐regulated genes (Villalobos *et al*., 2012; Wang and Estelle, 2014). Five *IAA* genes (e.g. *IAA6*,* 14*,* 29*,* 32* and *34*) displayed increased mRNA abundance in the *VvCEB1*_*opt*_‐overexpressing line (Figure 7a and Table S2), consistent with results observed for *VvCEB1* overexpression in wine grape embryos (Nicolas *et al*., 2013). However, other *AUX/IAA* genes were repressed in the *VvCEB1*_*opt*_‐overexpressing line. These results suggest a potentiation of the auxin‐signalling coreceptor complexes to mediate a diversity of auxin responses.

Auxin‐response factors, which function as activators of auxin‐responsive mRNA expression, are encoded by 23 genes in *Arabidopsis* (Remington *et al*., 2004). Some ARFs exhibit auxin‐responsive mRNA expression (Paponov *et al*., 2008). *ARF8* has been shown to negatively regulate cell number and expansion in flower petals of *Arabidopsis* via interactions with the bHLH TF *BIGPETALp* (Varaud *et al*., 2011). Only seven ARFs showed significantly altered mRNA abundance changes in the *VvCEB1*_*opt*_‐overexpressing line with all exhibiting reduced expression in flowers with the exception of *ARF12*, which showed increased expression in roots (Figure 7a). Thus, the reduced mRNA expression patterns observed for these ARFs suggest a linkage with the increased size of reproductive organs observed in the *Vv*CEB1_opt_‐overexpressing lines. Also, mRNA expression of *BT2* was induced dramatically in the *Vv*CEB1_opt_‐overexpressing line (Figure 7a and Table S2). *BT2* is an enhancer of certain auxin responses such as epinastic leaves, excessive root hairs and delayed flowering (Mandadi *et al*., 2009).

*SAUR* and *SAUR‐like* genes comprise the largest family of early auxin‐response genes, which appear to function as key regulators of diverse aspects of plant growth, development and senescence (Hagen and Guilfoyle, 2002; Ren and Gray, 2015). Some *SAUR* genes may function as positive effectors of cell elongation and expansion likely through the modulation of auxin transport (Chen *et al*., 2014; Spartz *et al*., 2012). Several members of this subfamily (e.g. *SAUR22* and *SAUR23*) showed increased mRNA expression in one or more tissues of the *VvCEB1*_*opt*_‐overexpressing line (Figure 7a and Table S3). SAUR41 also showed increased expression in all tissues of the *VvCEB1*_*opt*‐_overexpressing line. This SAUR gene promotes cell expansion and increases primary root growth and lateral root numbers when overexpressed (Kong *et al*., 2013). Other *SAUR* genes negatively regulate leaf cell expansion such as *SAUR36* (Hou *et al*., 2013). Many *SAUR* genes exhibited decreases in mRNA expression particularly in inflorescences, shoots and roots (Figure 7a and Tables S2–S4), and reductions in their expression might be related to the increased cell size in the *VvCEB1*_*opt*_‐overexpressing line through such negative regulatory processes.

*GRETCHEN HAGEN 3 (GH3)* genes consist of 19 auxin‐induced, auxin‐conjugating enzyme genes in *Arabidopsis* (Staswick *et al*., 2005). Two of the group II enzymes known to conjugate active IAA (e.g. *GH3.3* and *GH3.9*) showed increases in the *VvCEB1*_*opt*_‐overexpressing line suggesting responses consistent with increased auxin (Figure 7a and Tables S2, S4). These results are similar to the observed increases in mRNA abundance for IAA–amido synthetase (i.e. *GH3.2*,* GH3.3* and *GH3.9*) genes observed in *VvCEB1*‐overexpressing wine grape embryos (Nicolas *et al*., 2013).

Of the eight *PIN‐FORMED* (*PIN*) auxin efflux carrier genes in *Arabidopsis*, only three (i.e. *PIN1*,* PIN5* and *PIN8*) showed differential expression in the *VvCEB1*_*opt*_‐overexpressing line (Figure 7a and Tables S2, S4). Interestingly, *PIN5* showed enhanced expression in roots and both *PIN5* and *PIN8* showed decreased expression in inflorescences. *PIN5* and *PIN8* function to maintain intracellular auxin homeostasis for optimal pollen development and pollen tube growth possibly through negative effects on nuclear auxin signalling (Dal Bosco *et al*., 2012a,b; Ding *et al*., 2012). Of the four members of the *AUXIN IMPORT TRANSPORTER1* (*AUX1*) and *LIKE AUXIN RESISTANT* (*LAX*) genes encoding auxin influx carriers, *LAX1* showed increased mRNA abundance in leaves and inflorescences, whereas *LAX3* showed decreased transcript abundance in inflorescences (Figure 7a and Tables S2, S3). *AUX1/LAX* genes regulate vascular patterning and xylem differentiation and the degree of leaf margin serrations (Fàbregas *et al*., 2015; Kasprzewska *et al*., 2015) as well as female gametophyte development (Panoli *et al*., 2015). These changes in *AUX* transporter mRNA are likely linked to the observed increases in leaf teeth number and height in *VvCEB1*_*opt*_‐overexpressing lines (Figure S5).

### VvCEB1_opt_ overexpression increases global auxin content and lateral leaf primordia proliferation within meristems

To confirm the mechanistic basis of *VvCEB1*_*opt*_–overexpression reprogramming suggested by RNA‐Seq analyses that auxin is likely responsible for driving increased cell expansion and size (Ljung, 2013), auxin content was measured directly in roots and leaves and was found to increase significantly in both tissues (Figure 7b,c). To confirm these results, the *DR5rev::GUS* reporter system was employed to directly visualize increased auxin responses within the plants (Ulmasov *et al*., 1997). This synthetic auxin‐responsive promoter clearly resulted in increased GUS activity when expressed in both roots and leaves of *VvCEB1*_*opt*_‐overexpressing line #26 (Figure 7h–k) relative to the control line (Figure 7d–g). To confirm that the organ proliferation (e.g. increased leaf number) observed in the *VvCEB1*_*opt*_‐overexpressing line #26 was the direct result of increased auxin content, the *DR5rev::EYFP* reporter system was employed to directly visualize leaf primordia proliferation within vegetative meristems (Ulmasov *et al*., 1997; Vernoux *et al*., 2010). A significant increase in the number of lateral leaf primordia within meristems was obvious in the *VvCEB1*_*opt*_‐overexpressing line #26 relative to the control line (Figure S17). Taken together, these results confirmed that the increased organ size and biomass and likely reproductive capacity of the *VvCEB1*_*opt*_‐overexpressing lines arose from increased auxin accumulation and associated proliferation of leaf primordia relative to control lines.

### VvCEB1_opt_ overexpression increases aerial biomass in tobacco

**Figure 8:**
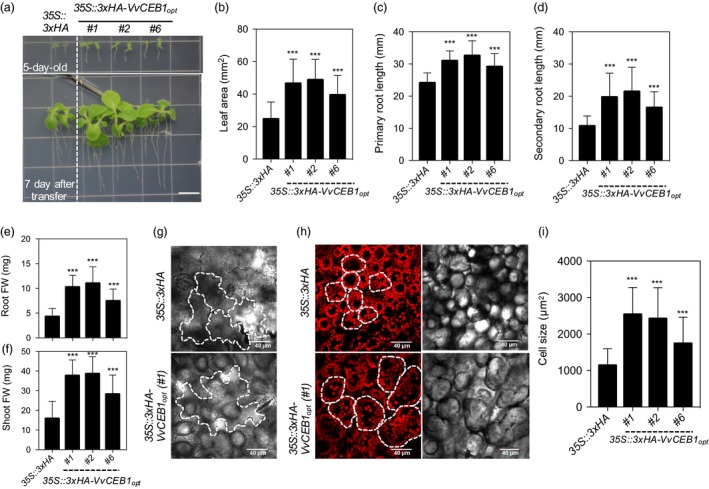
*Vv* CEB1_opt_ overexpression increases overall plant size by increasing cell size *in N. sylvestris* (flowering tobacco). (a) Representative images of the *35S::3xHA* empty‐vector (EV) line and the *35S::3xHA‐VvCEB1*_*opt*_ transgenic tobacco plants. Seeds (T_1_) were grown for 5 days on half‐strength MS medium containing kanamycin (200 mg/L) and transferred to kanamycin‐free half‐strength MS medium (top panel) and grown for 7 days (lower panel). Bar = 1 cm. (b) Quantification of the 1st leaf area (*n *=* *40). (c) Quantification of primary root length (*n *=* *40). (d) Quantification of the secondary root length (*n *=* *40). (e) Quantification of the root fresh weight (FW) (*n *=* *30). (f) Quantification of the shoot fresh weight (*n *=* *30). (g) Lower epidermis cell(s) of the EV and the *VvCEB1*_*opt*_‐overexpressing lines. White dotted lines indicate the shape of cells. (h) Palisade mesophyll cell(s) and chlorophyll autofluorescence of the EV and the *VvCEB1*_*opt*_‐overexpressing lines. White dotted lines indicate the shape of cells. (i) Quantification of the palisade mesophyll cell size (*n *=* *60). Values represent means ± SD and ***P < 0.001, one‐way anova with Dunnett's multiple comparison test.

The effects of *VvCEB1*_*opt*_ overexpression were also tested in a crop species, flowering tobacco (*N. sylvestris*), and resulted in significant increases in leaf area and primary and secondary root growth (Figure 8a–d). The *VvCEB1*_*opt*_‐overexpressing lines also showed significantly increased root and shoot fresh weights relative to the EV control line (Figure 8e–f). The *VvCEB1*_*opt*_‐overexpressing lines also showed increased size of palisade mesophyll cells relative to the EV control line (Figure 8g–i). These results show that *VvCEB1*_*opt*_ overexpression provides a generally applicable approach for improving biomass production in crops.

## Conclusions

Overexpression of a bHLH TF from wine grape, which is normally associated with fruit engustment, in *Arabidopsis* resulted in transcriptional reprogramming that led to a global increase in cell size and associated increases in leaf size and number, rosette size, hypocotyl width, root size and proliferation of lateral roots, larger reproductive organs, as well as greater flower and silique number. These larger organs resulted in an overall increase in vegetative biomass and seed production. RNA‐Seq analysis suggested that larger cell size is apparently driven by auxin‐mediated processes with accompanying changes to ionomic homeostasis to drive cellular expansion. This hypothesis was confirmed by direct measurement of auxin content, which was increased in roots and leaves of *VvCEB1*_*opt*_‐overexpressing lines leading to proliferation of lateral leaf primordia within meristems. Future studies aimed at identifying the direct target genes of *VvCEB1*_*opt*_ using chromatin immunoprecipitation sequencing (ChIP‐Seq), or related methods should help to better define the exact mode of action of this TF. Potential deployment of this biomass and seed yield improvement strategy to crops was demonstrated for flowering tobacco, suggesting that this approach could play a significant role in enhancing food and bioenergy crop production.

## Experimental procedures

### Codon optimization and synthesis of *VvCEB1*_*opt*_ gene

The coding sequence (CDS) of *VvCEB1* was codon‐optimized according to *Arabidopsis thaliana* ecotype *Columbia* (Col‐0) codon usage tabulated from the Codon Usage Database (http://www.kaxusa.or.jp/codon/) in GenBank. Rare codons within *VvCEB1* with frequencies of less than 0.15% were altered to more closely match those of *Arabidopsis* (Figure S1a). The codon‐optimized CDS of *VvCEB1* (*VvCEB1*_*opt*_) and 3xHuman influenza hemagglutinin (*3xHA*) protein tag with L1 and L2 attachment (Att) sites were synthesized by ATUM (formerly DNA 2.0) (https://www.ATUM.bio).

### Construction of cloning vectors with recombinant inserts

The *VvCEB1*_*opt*_ and *3xHA* tag were cloned into the binary vectors ImpGWB415 (*CaMV35S::3xHA*‐attR1‐attR2‐NOS terminator) and ImpGWB402 (*CaMV35S::attR1‐attR2‐NOS* terminator), respectively, using the Gateway™ LR Clonase™ II Enzyme Mix (Invitrogen, Carlsbad, CA) (Nakagawa *et al*., 2007). For the subcellular localization study, the *VvCEB*_*opt*_ coding region was also cloned into the binary vector ImpGWB405 (*CaMV35S::attR1‐attR2‐sGFP‐NOS* terminator) containing the C‐terminal synthetic green fluorescent protein (sGFP). Recombinant plasmids *35S::3xHA* (EV control), *35S::3xHA‐VvCEB1*_*opt*_ and *35S::VvCEB1*_*opt*_ *‐sGFP* were chemically transformed into *Escherichia coli* (NEB^®^ 10‐beta competent *E. coli*, New England BioLabs, Ipswich, MA) (Figure S1b–d). The plasmids were extracted and verified fully by DNA sequencing at the Nevada Genomics Center (Reno, NV). Each plasmid was chemically transformed into the *Agrobacterium tumefaciens* strain GV3101 for *Agrobacterium*‐mediated transformation of *A. thaliana* (Col‐0).

For the *DR5rev::GUS* and the *DR5rev::EYFP* vectors, the DR5rev (9 × TGTCTC) element (Ulmasov *et al*., 1997) was PCR‐amplified and cloned into the D‐TOPO™ vector (Invitrogen). DR5rev entry clones were cloned into the binary vectors ImpGWB533 (attR1‐attR2‐GUS‐NOS terminator) and ImpGWB540 (attR1‐attR2‐EYFP‐NOS terminator), respectively. Each DR5rev reporter construct was transformed into T_3_ homozygous lines of *35S::3xHA* (EV) and *35S::3xHA‐VvCEB1*_opt_ (#26), respectively. DR5 reporter lines were selected on full‐strength Murashige and Skoog (MS) basal medium containing Gamborg's vitamins (pH = 5.7), 1% sucrose and 7 g/L phytoagar (M404; Phytotechnology Laboratories, Shawnee Mission, KS) containing 25 μg/mL hygromycin in a growth chamber (Model CU‐32L; Percival Scientific, Inc., Perry, IA) under a 16‐h photoperiod (16‐h light 135 μmol/m^2^/s/8‐h dark).

### *Arabidopsis* and *Nicotiana* transformation

*Agrobacterium* harbouring *35S::3xHA*,* 35S::3xHA‐VvCEB1*_*opt*_ or *35S::VvCEB1*_*opt*_ *‐sGFP* were used to transform these plasmids into *A. thaliana* (Col‐0) using the floral dip method (Zhang *et al*., 2006a). T_1_ transformants were selected following growth on full‐strength Murashige and Skoog (MS) basal medium containing Gamborg's vitamins (pH = 5.7), 1% sucrose and 7 g/L phytoagar (M404; Phytotechnology Laboratories) containing 50 μg/mL kanamycin in a growth chamber (Model CU‐32L; Percival Scientific, Inc.) under 16‐h/8‐h (135 μmol/m^2^/s light/dark) cycles at 23 °C/21 °C (day/night). Lines with T_2_ progeny segregating 3 : 1 for kanamycin resistance/sensitivity were further propagated, and four independent T_3_ homozygous seed lines of *35S::3xHA‐VvCEB1*_*opt*_ (#20, #25, #26 and #30) and one homozygous seed line of *35S::3xHA* were selected for further study. All transgenic seed lines and Col‐0 wild‐type (wt) seeds were harvested at the same time to minimize differences in seed quality and were subsequently used for phenotypic characterization. *Nicotiana sylvestris* plants expressing the *35S::3xHA* and *35S::3xHA‐VvCEB1*_*opt*_ constructs were transformed as described (Zhang *et al*., 2012). Seeds (T_1_) were germinated and grown on half‐strength MS containing kanamycin (200 mg/L) for 5–7 days to select for transformants.

### Seed sterilization and plant growth conditions

For *in vitro*‐grown conditions, seeds were incubated with sterilized water and subjected to 4 °C for 3 days in darkness. After seed stratification, seeds were sterilized in 70% ethanol for 1 min followed by 20% (v/v) household bleach for 3 min and then were rinsed five times with sterilized water. For the long‐day growth conditions, seedlings were grown on full‐strength MS basal medium containing Gamborg's vitamins (pH = 5.7), 1% sucrose and 7 g/L phytoagar in a Percival Scientific Model CU‐32L growth chamber under a 16‐h photoperiod (16‐h light 135 μmol/m^2^/s/8‐h dark) cycles at 23 °C/21 °C (day/night). For the half‐day growth conditions, seedlings were grown in a growth chamber under a 12‐h photoperiod (12‐h light 135 μmol/m^2^/s/12‐h dark) cycles at 23 °C/21 °C (day/night). Growth media specific to various experiments are indicated below.

### Subcellular localization

T_1_ *35S::VvCEB1*_*opt*_ *‐sGFP* seedlings were screened on the full‐strength MS basal medium containing Gamborg's vitamins (pH = 5.7), 1% sucrose and 7 g/L phytoagar in a Percival Scientific Model CU‐32L growth chamber under a 16‐h photoperiod. The roots of surviving transgenic plants were stained with 4′,6‐diamidino‐2‐phenylindole (DAPI) solution (Fluoroshield™ with DAPI; Sigma‐Aldrich, St. Louis, MO) for 10 min at room temperature. Samples were observed using confocal laser‐scanning microscopy (FluoView™ FV1000; Olympus, Tokyo, Japan). DAPI and GFP were excited at 405 and 488 nm with a laser, respectively. The fluorescence emission was collected at 461/50 nm for DAPI and 510/50 nm for GFP.

### RNA extraction and real‐time quantitative PCR

Seeds of the four *VvCEB1*_*opt*_‐overexpressing, Col‐0 wild‐type and EV control lines were germinated and grown on full‐strength MS basal medium containing Gamborg's vitamins (pH = 5.7), 1% sucrose and 7 g/L phytoagar in a Percival Scientific Model CU‐32L growth chamber under a 16‐h photoperiod. Total RNA was extracted from 100 mg tissue samples of whole seedlings using the RNeasy^®^ Plant Mini Kit (Qiagen, Valencia, CA). First‐strand cDNA synthesis from 500 ng of total RNA was performed using the iScript Advanced cDNA Synthesis Kit (Bio‐Rad Laboratories, Hercules, CA), following the manufacturer's instructions. Real‐time qPCR was performed using SsoAdvanced SYBR Green Supermix (Bio‐Rad Laboratories), and the SYBR signals were monitored using a C1000 Thermal Cycler and CFX96 Real‐Time System detection instrument (Bio‐Rad Laboratories). The following standard thermal profile was used for PCR reactions: 95 °C for 30 s, 40 cycles of 95 °C for 5 s and 60 °C for 30 s. Amplicon dissociation curves (i.e. melting curves) were recorded after cycle 40 by heating from 65 °C to 95 °C in 0.5 °C increments with 3 s per step. Expression levels for the *VvCEB1*_*opt*_ gene in four transgenic lines were normalized using the *Arabidopsis TIP41‐like* (AT4G34270) gene as standard (Czechowski *et al*., 2005). The following primer pairs were used: *VvCEB1*_*opt*_ (5′‐GCAGCGCTGTTATCACAAGT‐3′ and 5′‐GAGGGTGAAGGTGGTGAGAC‐3′) and *Arabidopsis TIP41‐like* (5′‐GTGAAAACTGTTGGAGAGAAGCAA‐3′ and 5′‐TCAACTGGATACCCTTTCGCA‐3′).

### Protein extraction and immunoblot analysis

Whole seedlings were grown on full‐strength MS basal medium containing Gamborg's vitamins (pH = 5.7), 1% sucrose and 7 g/L phytoagar in a Percival Scientific Model CU‐32L growth chamber under a 16‐h photoperiod, harvested and homogenized in liquid nitrogen. Total proteins were extracted from 3‐week‐old seedlings. Tissue samples (500 mg) were quickly incubated with 1 mL of modified denaturing buffer [50 mm 2‐amino‐2‐(hydroxymethyl)‐1,3‐propanediol‐HCl, pH 7.5, 250 mm NaCl, 0.1% IGEPAL CA‐630, 4 m urea, 10 mm NaPO4, pH = 6.0, and 1× protease inhibitor cocktail (Roche Applied Science, Indianapolis, IN)] for 10 min and were centrifuged for 30 min at 16 000 *× g* at 4 °C for 30 min (Lim *et al*., 2015). The soluble supernatant fraction was moved to a new microcentrifuge tube, and protein concentration was determined using the Pierce BCA Protein Assay Kit (Thermo Fisher Scientific, Rockford, IL). All protein samples were then adjusted to a final concentration of 1.0 μg/μL in Laemmli sample buffer. Each sample (10 μL) was separated by SDS‐PAGE using 4%–15% polyacrylamide gels (Bio‐Rad) and then transferred to nitrocellulose blotting membrane (Bio‐Rad). Immunoblot analysis was conducted using anti‐HA primary antibody (clone 3F10; Roche Diagnostics Corp., Indianapolis, IN; 1 : 2000), with a secondary goat anti‐rat lgG (EMD Millipore, Billerica, MA) and anti‐actin antibody (Ab197345; Abcam, Cambridge, MA; 1 : 500) with a secondary donkey anti‐rabbit lgG (Amersham Life Science, Arlington Heights, IL). Membranes were exposed to the Pierce Enhanced Chemiluminescence Reagent (Thermo Fisher Scientific) for 1 min at room temperature and visualized using a ChemiDoc MP Imaging System (Bio‐Rad). Actin immunodetection and total proteins stained with Ponceau S (Fisher Biotech, Fair Lawn, NJ) were used as loading controls.

### Growth conditions and quantification

For the seed germination assays, sterilized seeds of each line were plated on full‐strength MS basal medium containing Gamborg's vitamins (pH = 5.7), 1% sucrose and 7 g/L phytoagar and grown in a Percival Scientific Model CU‐32L growth chamber under a 16‐h photoperiod. Seed germination rates were scored each day for 7 days after sowing.

For whole‐plant biomass assays, seedlings of each line were grown on full‐strength MS basal medium containing Gamborg's vitamins (pH = 5.7), 1% sucrose and 7 g/L phytoagar in a Percival Scientific Model CU‐32L growth chamber under a 16‐h photoperiod. Fresh weights of 30 1‐week‐old seedlings, ten 2‐week‐old seedlings and individual 3‐week‐old plants for each line were determined after germination. After measuring fresh weights, plants were fully dehydrated at 60 °C for 24 h and dry weights were measured.

For phenotypic analysis of vegetative aerial organs, seeds of each line were directly sown and grown in soil (Sunshine 781, custom blend, 45%–50% peat moss, Scotts‐Sierra Horticultural Products, Marysville, OH) in 89‐mm square plastic pots (Kord, Inc., Toronto, CA) in a Percival Scientific Model AR‐75L2 growth chamber under a 16‐h photoperiod. Four‐week‐old rosettes and detached leaves were photographed to measure leaf number, rosette diameter and the area of fifth fully expanded true leaves. To measure the thicknesses of leaves and inflorescence stems of *VvCEB1*_*opt*_‐overexpressing lines, seeds were germinated and grown in soil under a 12‐h photoperiod. For analysis of leaf thickness and inflorescence stem thickness, the fifth fully expanded true leaves from 4‐week‐old plants and primary inflorescence stems were measured using a digital micrometer (Model PK‐1015, Mitutoyo Corp., Kawasaki, Japan).

To quantify leaf area, single plants of each of the *VvCEB1*_*opt*_‐overexpressing lines were grown together in the same pot with single plants of the EV control line for 4 weeks after germination. Detached leaves were photographed, and leaf area was measured using ImageJ software (http://imagej.nih.gov/ij/).

For hypocotyl growth measurements, plants were germinated and grown in vertical position on plates on half‐strength MS basal media containing Gamborg's vitamins (pH = 5.7), 1% sucrose and 7 g/L phytoagar in a Percival Scientific Model CU‐32L growth chamber under a 16‐h photoperiod. Fourteen‐day‐old plants were photographed, and hypocotyl lengths and widths were measured using ImageJ software.

For root elongation measurements, sterilized seeds were germinated and grown in vertical position on half‐strength MS basal medium containing Gamborg's vitamins (pH = 5.7), 1% sucrose and 7 g/L phytoagar in a Percival Scientific Model CU‐32L growth chamber under a 16‐h photoperiod. Seedlings were scanned at 3, 5, 7 or 14 days after germination, and root lengths, widths and lateral root numbers were quantified using ImageJ software. To measure root biomass, sterilized seeds were grown vertically on full‐strength MS basal medium containing Gamborg's vitamins (pH = 5.7), 1% sucrose and 7 g/L phytoagar in a Percival Scientific Model CU‐32L growth chamber under a 16‐h photoperiod. Fresh weights and dry weights were determined using detached roots from 3‐week‐old plants.

### Root meristem and mature root cell analysis

Seeds of the *VvCEB1*_*opt*_‐overexpressing line #26 and the EV control line were germinated and grown on half‐strength MS basal medium containing Gamborg's vitamins (pH = 5.7), 1% sucrose and 7 g/L phytoagar in a Percival Scientific Model CU‐32L growth chamber under a 16‐h photoperiod for 2 weeks. The lipophilic probe, *N*‐(3‐triethylammoniumpropyl)‐4‐(6‐(4‐(diethylamino) phenyl) hexatrienyl) pyridinium dibromide (FM4‐64; Thermo Fisher Scientific), was used to stain the plasma membranes of cells in the root. FM4‐64 was added to half‐strength liquid MS basal medium containing Gamborg's vitamins (pH = 5.7) at a final concentration of 10 μm, and the detached roots were incubated in the FM4‐64 solution at room temperature for 2 h to stain the root cells and for 5 h to stain the root vacuoles. Unbound FM4‐64 was washed out three times using sterile deionized water. The roots were stained again with propidium iodide (PI, P4170, 100 μg/mL, Sigma‐Aldrich, Corp.) for 1 min to stain nuclei. Root samples were washed three times with sterile deionized water, and samples were mounted in 50% (v/v) glycerol on glass microscope slides. Images of root meristems and root cells in the maturation zone were captured *via* confocal laser‐scanning microscopy (FluoView™ FV1000; Olympus, Inc., Center Valley, PA). FM4‐64 and PI were excited at 543 nm with a laser, and the fluorescence emission was collected at 612 nm (range 100 nm). Meristem length and cell number were determined for cortical cells as described (Hacham *et al*., 2011). Cortex width in the apical meristem was directly measured using ImageJ software.

### Cell size and number analysis

To measure palisade mesophyll and spongy mesophyll cell sizes per unit area, the fifth fully expanded true leaves were sampled from 4‐week‐old plants of each line grown in soil in a Percival Scientific Model AR‐75L2 growth chamber under a 12‐h photoperiod. Transverse sections of leaves from *VvCEB1*_*opt*_‐overexpressing lines and the EV control line were cut as described (Zambrano *et al*., 2014) with some modifications. Small pieces of leaf (approximately 1–2 × 2–3 mm) were fixed in 1.5% (v/v) glutaraldehyde solution by vacuum infiltration for 30 min and incubated at 4 °C for 16 h. Nine leaf samples of each line were dehydrated in an ethanol series of increasing concentrations (20%, 30%, 50%, 70%, 95% and 100%) for 20 min at each concentration. The samples were then embedded in Epon/Spurr's combination resin formula (Ted Pella, Inc., Redding, CA). Sections with 1 μm thickness were cut using an ultramicrotome (Ultracut UCT; Leica Biosystems Inc., Buffalo Grove, IL) with a diamond knife (Diatome AG, Biel, Switzerland) and stained with toluidine blue O. Images were captured under 10× and 20× magnifications by light microscopy (Eclipse E400; Nikon Inc., Melville, NY) and analysed for cell size and number.

To measure cell size and the number of cells in the upper and lower epidermis, tangential sections of leaves were taken using the fifth fully expanded true leaves from 4‐week‐old plants grown in soil. Leaves were sampled and photographed to estimate total cell numbers per leaf. Small pieces of leaf (approximately 0.5 × 0.5 cm) were submerged in PI solution (100 μg/mL; Sigma‐Aldrich) and subjected to vacuum infiltration for 20 min. Samples were washed three times with sterilized water, and images were captured by laser‐scanning confocal microscopy (Olympus FluoView™ FV 1000). PI was excited at 543 nm with a laser, and the fluorescence emission was collected at 612 nm (range 100 nm). Cell outlines were drawn on both transverse and tangential leaf sections using a Wacom Cintiq 13HD tablet (http://www.wacom.com/) display to trace the cell size and number. Cell size was measured using ImageJ software.

### Inflorescence stem analysis

Inflorescence stem samples were obtained from 3‐week‐old plants from both EV control line and *VvCEB1*_*opt*_‐overexpressing lines. The samples were fixed overnight at 4 °C in FAA fixative (10% v/v formalin, 5% v/v acetic acid and 50% v/v ethanol) with 0.03% v/v Tween 20. The fixed samples were paraffin‐embedded (Paraplast Plus^®^; Leica Biosystems Inc.) after being dehydrated in a series of increasing concentration of ethanol (from 10% to 100% in 10% increments followed by 25%, 50% and 100% tert‐butyl alcohol (TBA) series. The embedded samples were cut into 10‐μm sections using Leica RM 2145 microtome (Leica Biosystems Inc.). The sections were stained using 0.25% w/v toluidine blue in 1× phosphate‐buffered saline (PBS) buffer (137 mm NaCl, 2.7 mm KCl,10 mm Na_2_HPO_4_, 2 mm KH_2_PO_4_) after being rehydrated in a series of decreasing concentration of ethanol (from 100% to 10% in 10% decrements), followed by ddH_2_O and 1× phosphate‐buffered saline (PBS). Bright‐field images of the stained sections were captured using Keyence BZ‐X710 microscope (Keyence Corporation of America, Itasca, IL).

### Polyploidy analysis

Four independent Col‐0 wild‐type, EV control and *VvCEB1*_*opt*_‐overexpressing lines were grown on half‐strength MS basal medium containing Gamborg's vitamins (pH = 5.7) (M404; Phytotechnology Laboratories), 1% sucrose and 0.7% phytoagar in a Percival Scientific Model CU‐32L growth chamber under a 16‐h photoperiod. Roots and leaves were sampled separately for each line in three replicates and placed in between moist paper towels in a Ziploc bag. Samples were analysed by Benaroya Research Institute (Flow Cytometry and Imaging Core Laboratory, Seattle, WA). Briefly, intact nuclei suspensions were prepared by chopping plant tissues and lysing protoplasts in MgSO_4_ buffer. Chicken erythrocyte nuclei were used as an internal standard for these measurements. Nuclear DNA content was performed using a FACSort Flow Cytometer (Becton Dickinson, Inc., Franklin Lakes, NJ) as described (Arumuganathan and Earle, 1991).

### Transmission electron microscope analysis

To analyse the cell wall thickness of palisade mesophyll cells and starch granules in chloroplasts, 4‐week‐old plants were grown in soil in a Percival Scientific Model AR‐75L2 growth chamber under a 12‐h photoperiod. Small pieces of leaves (approximately 1–2 × 2–3 mm) were fixed in 1.5% (v/v) glutaraldehyde solution by vacuum infiltration for 30 min and incubated at 4 °C for 16 h. Tissues were rinsed with 0.1 m PO_4_ buffer and post‐fixed for 2 h in 1% phosphate‐buffered osmium tetroxide. Tissue samples were dehydrated by an ethanol series of increasing concentrations (20%, 30%, 50%, 70%, 95% and 100%), followed by three changes of 100% ethanol for 10 min each, and transitioned in 1 : 1 with ethyl alcohol : propylene oxide (PO) for 10 min. Dehydration was completed using two changes of 100% PO. Infiltration began using Epon [Cat. #14900; Epon 812 Substitute, Electron Microscopy Sciences (EMS), Hatfield, PA]/Spurr's resin (Cat. # 18306‐4112, ERL 4221; Ted Pella, Inc.) in 100% PO at a 1 : 3 ratio heated by microwave (Heumann, 1992; Russin and Trivett, 2001) at 100 watts for 5 min, followed by a 1 : 1 ratio of Epon/Spurr's resin : PO heated at 100 watts for 5 min, followed by vacuum infiltration and storage overnight at 4 °C. Infiltration was continued in a 3 : 1 ratio of Epon/Spurr's resin : PO heated at 250 watts for 5 min. Lastly, three changes of 100% Epon/Spurr's resin with microwave heating were performed before transferring samples to capsules in cross‐sectional orientation, which were then polymerized for 2 days at 70 °C. Ultrathin sections of the polymerized blocks were cut using a Diatome diamond knife (Diatome and EMS) and an ultramicrotome (Leica Ultracut UCT, Leica, Vienna, Austria) and were picked up on 150‐mesh copper grids. The sections were stained with uranyl acetate and lead citrate before viewing on a Phillips CM120 BioTwin transmission electron microscope (Hillsboro, OR) (Bozzola and Russell, 1992). Micrographs were taken using a Gatan MegaScan 794/20 digital camera (Pleasanton, CA).

### Chloroplast numbers and chlorophyll assays

To measure chloroplast numbers per palisade mesophyll cell and chlorophyll contents, plants were grown in soil for 4 weeks in a Percival Scientific Model AR‐75L2 growth chamber under a 12‐h photoperiod. To count chloroplasts, plants were incubated in the dark for 1 h and the fifth fully expanded true leaves were detached and immediately immersed in 3.7% (v/v) formalin/5% (v/v) acetic acid/50% (v/v) ethanol (FAA) fixative and then subjected to a light vacuum until the leaf tissues sank in the vial (Bomblies *et al*., 2008). Fixed leaf samples were observed using confocal laser‐scanning microscopy (FluoView™ FV1000; Olympus). Chlorophyll autofluorescence in palisade mesophyll cells was fully captured at 5‐μm intervals through the Z‐stack. Chloroplasts were counted using all the images within a single stack to avoid duplicate or uncounted chloroplasts.

For the chlorophyll assay, fresh leaves (300 mg) were ground in liquid nitrogen and incubated in 5 mL of 80% acetone in the dark for 30 min. The supernatant was transferred to a new tube, and the acetone extraction step was repeated. All supernatants from each sample were combined into a single tube, and chlorophyll *a* and *b* contents were measured using a NanoDrop™ 8000 spectrophotometer (Thermo Fisher Scientific). The chlorophyll concentrations were calculated as described (Ni *et al*., 2009).

### Carbohydrate assays

To quantify total sugars and starch contents of leaves, plants were germinated and grown in soil for 4 weeks in a Percival Scientific Model AR‐75L2 growth chamber under a 12‐h photoperiod. Carbohydrate assays were conducted using leaf samples as described (Dubois *et al*., 1956; Fox and Robyt, 1991) with some modifications. Briefly, fully expanded leaves were harvested and ground in liquid nitrogen. Each 500 mg leaf sample was incubated in 50% (v/v) methanol at 80 °C for 30 min. After centrifugation at 3000 × *g* for 10 min, 50 μL of supernatant was mixed with an equal volume of 5% (v/v) phenol and the mixture was incubated in 250 μL of sulphuric acid at 80 °C for 30 min. Total sugar contents were measured at 490 nm using a Wallac 1420 Multilabel Counter (PerkinElmer, Shelton, CT). For starch extraction, leaf pellets were washed three times with sterile water and then homogenized in acetate buffer (pH = 4.5) using a Polytron^®^ PT 1200E (Kinematica, Littau, Switzerland). Starch was digested in an enzyme solution [acetate buffer (pH = 4.5), 300 U of α‐amyloglucosidase (Sigma‐Aldrich) and 25 U of α‐amylase (Sigma‐Aldrich)] at 45 °C for 16 h. Digested starch contents were measured as free sugars at 490 nm as described above.

### Total protein extraction and quantification

Plants were grown in soil for 4 weeks in a Percival Scientific Model AR‐75L2 growth chamber under a 16‐h photoperiod. Fully expanded fifth leaves were harvested and homogenized in liquid nitrogen, and oven‐dried leaves and seeds were ground using a mortar and pestle. Each sample was incubated with 0.5 mL of denaturing buffer for 10 min as described above. Samples were centrifuged for 30 min at 16 000 *× g* at 4 °C for 30 min. Total protein amount was determined using the Pierce BCA Protein Assay Kit (Thermo Fisher Scientific).

### Physiological analysis of reproductive capacity

The flowering time of the primary inflorescence stem was scored over the course of 6 weeks after germination. Plants were germinated and grown in soil or on half‐strength MS basal medium containing Gamborg's vitamins (pH = 5.7), 1% sucrose and 7 g/L phytoagar in a Percival Model AR‐75L2 growth chamber under a 16‐h or 12‐h photoperiod, respectively.

Seeds were germinated and plants grown in soil in individual 89‐mm square plastic pots in a Percival Model AR‐75L2 growth chamber under a 12‐h photoperiod. Plants were grown continuously under well‐watered conditions until physiological maturity. Fully developed flowers and siliques from primary inflorescence stems were photographed using a zoom stereomicroscope (SMZ800; Nikon Instruments Inc.). Individual flower and silique sizes and seed area were measured from scanned images using ImageJ software. Seeds were harvested at maturity, and 100‐seed weight and total seed yield per plant were measured using an electronic analytical balance (AS313; Ohaus Corp., Parsippany, NJ).

### Sample collection and RNA extraction for transcriptome analysis

To compare the transcriptome profiles, RNA‐sequencing (RNA‐Seq) analysis was performed on three different plant organs: leaf, root and primary inflorescence. Three seeds each of *VvCEB1*_*opt*_‐overexpressing line #26 and Col‐0 wild‐type and EV control lines were germinated and grown together in 250 mL of full‐strength MS basal medium containing Gamborg's vitamins (pH = 5.7), 1% sucrose and 7 g/L phytoagar in plastic tissue culture vessels (C211; Phytotechnology Laboratories, Inc.) under a 12‐h photoperiod. Leaf and root samples were collected from 3‐week‐old plants before bolting. The primary inflorescences included flowers, siliques, cauline leaves and secondary branches of Col‐0 wild‐type and EV control lines collected from 6‐week‐old plants. The inflorescences of *VvCEB1*_*opt*_‐overexpressing line #26 were sampled from 8‐week‐old plants at the same developmental stage compared to the control lines. Three biological replicates were conducted for each organ type for each line. Total RNA was isolated from 100 mg leaf, root and inflorescences tissue samples using the RNeasy Plant Mini Kit (Qiagen). All RNA‐Seq libraries for these 27 samples were prepared using the TrueSeq RNA Library Preparation Kit version 2 (Illumina, San Diego, CA), following the manufacturer's protocol. All cDNA libraries were pooled together and sequenced on two high‐output flow cells using an Illumina NextSeq 500 instrument with 150‐bp paired‐end read lengths using 302 cycles plus six cycles for barcoded multiplexed samples.

### Read preprocessing for transcriptome analysis

Raw Illumina reads were obtained from BaseSpace Sequence Hub (https://basespace.illumina.com/home/index). Reads were preprocessed using Trimmomatic software (version 0.36) (Bolger *et al*., 2014). Adapters and low‐quality sequences were filtered out with the minimum Phred‐like quality score (*Q*‐score) of 20 and minimum length of 50 bp. Only trimmed paired‐end reads were retained for all subsequent analysis (Figure S18).

### Read mapping and differential gene expression for transcriptome analysis

Trimmed reads were aligned to an *A. thaliana* annotation obtained from Araport (Araport11 Pre‐release 3) (Krishnakumar *et al*., 2015) using Bowtie2 (version 2.2.4) (Langmead and Salzberg, 2012). The results were sorted using SAMtools (version 0.1.19‐44428 cd) (Li *et al*., 2008), and read counts for each sample were calculated using RSEM (version 1.2.25) (Li and Dewey, 2011). EM read counts per gene were used for downstream analysis. Differential gene expression was carried out using four different R statistical packages, DESeq2 (version 1.6.3) (Love *et al*., 2014), edgeR (version 3.8.6) (Robinson *et al*., 2010), ROTS (version 1.1.2) (Seyednasrollah *et al*., 2016) and voom (version 3.22.7) (Law *et al*., 2014). A false discovery rate (FDR) cut‐off of <0.01 and fold change of ±2 were used to identify differentially expressed genes with all four R programs. These criteria were guided by comparisons within biological sample replicates rather than among samples (Figure S19). The quality of samples was then assessed using a modified Trinity pipeline (Figure S20) (Haas *et al*., 2013). The principal component analysis data of the sample correlation matrix were generated from log_2_‐transformed FPKM values standardized by Z‐score (Figure S21) (Ma and Dai, 2011).

### Evaluation of differential gene expression using different algorithms

To identify robust differential gene expression, a three‐step evaluation was applied. With an FDR cut‐off of <0.001 and fold change in gene expression of ±2, wild‐type Col‐0 was compared with a *VvCEB1*_*opt*_‐overexpressing line #26, the *35S::3xHA* EV control line was compared with a *VvCEB1*_*opt*_‐overexpressing line, and wild‐type Col‐0 was compared with the *35S::3xHA* EV control line for three tissue types. Four‐way Venn diagrams were generated with four different algorithms, resulting in nine Venn diagrams (Figure S23). Genes that were detected as differentially expressed with more than two algorithms for each tissue type with no overlapping expression with Col‐0 wild‐type *versus* the EV control lines were treated as true differentially expressed genes and were considered tissue‐specific differentially expressed genes. Three‐way Venn diagrams were constructed to identify differentially expressed genes with overlapping expression in leaves, roots and primary inflorescence stems or those with tissue‐specific expression. All Venn diagram analyses and visualization were performed using jvenn (Bardou *et al*., 2014).

### Creation and visualization of gene networks

Interaction networks were constructed using PPIs from the Biological General Repository for Interaction Datasets (BioGrid, *A. thaliana*, ver. 3.4.137) (Chatr‐Aryamontri *et al*., 2015) and gene regulatory information from the *Arabidopsis* Gene Regulatory Information Server (AGRIS, accessed 19 May 2015) (Yilmaz *et al*., 2011). The binary PPI networks were constructed by connecting all first‐degree interacting proteins within each of the differentially expressed gene sets. Next, the results of the binary PPIs were enhanced to include gene regulatory information to extend the interaction network using information derived from the published literature. Networks were visualized using Cytoscape (version 3.4.0) (Shannon *et al*., 2003), and native NetworkAnalyzer was used to define analysis network properties, including edge connectivity and topology coefficients. Nodes were weighted to represent edge connectivity, and edges were indicated from literature‐derived annotations. Node colour and shape indicate the mode of regulation and associated TFs, respectively. The expression heat maps were drawn using Pretty heat map (pheatmap, version 1.0.8) (Kolde, 2015) using the correlation distances and Ward.D2 cluster analysis method (Murtagh and Legendre, 2014). The MCODE plug‐in was used to further divide the network into modules, using a cut‐off value for the connectivity degree of the nodes (Bader and Hogue, 2003). The GO enrichment analysis was performed with a threshold *P *<* *0.05 based on a hypergeometric test, corrected for multiple comparisons using the Holm–Bonferroni FDR in ThaleMine, Araport (Krishnakumar *et al*., 2015).

### Auxin quantitative assay

To compare the auxin amounts within the wild‐type Col‐0, EV control and *VvCEB1*_*opt*_‐overexpressing lines (#26), leaf and root were harvested, frozen and ground in liquid nitrogen. Four biological replicates were conducted for each organ type for each line. Free IAA in plant tissues was measured using a Thermo TSQ vantage GC‐MS/MS (GC‐triple quadruple MS). Auxin content was measured by the Plant Metabolomics Facility at the University of Minnesota as described (Liu *et al*., 2013).

### Ionomic analysis

Col‐0 wild‐type, EV control and four *VvCEB1*_*opt*_‐overexpressing lines were germinated and grown in soil in the greenhouse under a 16‐h photoperiod. Four‐week‐old leaves were harvested and oven‐dried at 65 °C for 24 h. A 500 mg of dried leaf samples was used to determine ion elements (Al, As, B, Ba, Be, Ca, Cd, Co, Cr, Cu, Fe, K, Mg, Mn, Mo, Na, Ni, P, Pb, Rb, S, Si, Sr, Ti, V and Zn) using the wet ashing method by inductively coupled plasma‐optical emission spectrometry (ICE‐OES). Ion analysis was performed using an iCap 7600 Duo ICP‐OES analyser by the Research Analytical Laboratory at the University of Minnesota (http://ral.cfans.umn.edu) as described (Munter and Grande, 1981; Munter *et al*., 1984). Chloride concentration was determined after extraction with CaSO4 as described (U.S. Environmental Protection Agency, 1979).

### DR5 reporter assays

For the histochemical β‐glucuronidase (GUS) assay, 10‐day‐old hygromycin‐resistant plants of the *DR5rev::GUS/EV* and the *DR5rev::GUS/35S::3xHA‐VvCEB1*_*opt*_ (#26) were selected and treated with 90% acetone on ice for 30 min, then washed once for 10 min in GUS staining buffer (50 mm sodium phosphate buffer, pH 7.0, 4 mm EDTA, 1 mm K_4_Fe(CN)_6_, 1 mm K_3_Fe(CN)_6_ and 0.1% Triton X‐100) and then incubated at 37°C for 16 h in GUS staining buffer plus 1 mg/mL X‐Gluc (5‐Bromo‐4‐chloro‐3‐indolyl glucuronide, X871; Phytotechnology Lab, Overland Park, KS) (Jefferson *et al*., 1987). Tissue was dehydrated in a series of increasing concentrations of ethanol (70%, 80%, 90% and 100% v/v) over 2 days. Images were photographed using a microscope (BZ‐X700; Keyence).

For the *DR5rev::EYFP* imaging in the shoot apical meristem, 2‐week‐old hygromycin‐resistant plants were used and images were captured using Zeiss Axio Zoom version 16 stereomicroscope (Carl Zeiss AG, Thornwood, NJ). EYFP and chloroplast autofluorescence were excited at 470/40 and 572/25 nm, and the fluorescence emission was collected at 425/50 and 629/62 nm, respectively.

### Accession numbers

Raw sequencing data reported in this study are available in the Sequence Read Archive (http://www.ncbi.nlm.nih.gov/sra) under BioProject Accession Number PRJNA325158.

## Conflict of interest

The authors have no conflict of interests to declare.

## Supporting information


**Figure S1** Subcellular localization of codon‐optimized *VvCEB1–sGFP* fusion protein in *A. thaliana*.
**Figure S2** Characterization of the wild‐type, 35S*::3xHA* empty‐vector control line and *VvCEB1*_opt_‐overexpressing *Arabidopsis* lines.
**Figure S3** Seed germination rates of wild‐type, 35S*::3xHA* empty‐vector control line, and *VvCEB1*_opt_‐overexpressing *Arabidopsis* lines.
**Figure S4** *VvCEB1*_*opt*_ overexpression in *Arabidopsis* increases shoot biomass.
**Figure S5** *VvCEB1*_*opt*_‐overexpressing *Arabidopsis* plants exhibit increased leaf teeth number and height along the leaf margin.
**Figure S6** *VvCEB1*_*opt*_‐overexpressing *Arabidopsis* plants exhibit hypocotyls with decreased length and increased width.
**Figure S7** *VvCEB1*_*opt*_‐overexpressing *Arabidopsis* plants exhibit increased root biomass.
**Figure S8** *VvCEB1*_*opt*_‐overexpression in *Arabidopsis* plants increases root cell size.
**Figure S9** *VvCEB1*_*opt*_‐overexpressing *Arabidopsis* plants have increased leaf thickness and cell size, but lack increased ploidy.
**Figure S10** *VvCEB1*_*opt*_‐overexpressing *Arabidopsis* plants exhibit increased chlorophyll contents on a per leaf or per plant basis.
**Figure S11** *VvCEB1*_*opt*_‐overexpressing *Arabidopsis* plants exhibit decreased cell wall thickness.
**Figure S12** *VvCEB1*_*opt*_ overexpression alters the concentration of Ca, K, P, S, and Mo in *Arabidopsis* leaves.
**Figure S13** *VvCEB1*_*opt*_‐overexpressing *Arabidopsis* plants exhibit increased sepal number and flower width.
**Figure S14** *VvCEB1*_*opt*_‐overexpressing *Arabidopsis* plants exhibit increased primary inflorescence stem thickness by increasing cell size.
**Figure S15** *VvCEB1*_*opt*_‐overexpressing *Arabidopsis* plants exhibit delayed bolting under half‐day and long‐day conditions.
**Figure S16** *VvCEB1*_*opt*_‐overexpressing *Arabidopsis* plants have increased soluble sugar and decreased starch contents.
**Figure S17** *VvCEB1*_*opt*_‐overexpressing *Arabidopsis* plants have increased lateral leaf primordia within the shoot apical meristem (SAM).

**Figure S18** Summary of total RNA‐Seq read counts per tissue type, read mapping rate, and read quality for individual samples.
**Figure S19** Testing for two‐fold differential expression within biological sample replicates between RNA‐Seq samples from each organ type.
**Figure S20** RNA‐Seq expression correlation matrix heat maps of RNA‐Seq samples generated by four different differential expression analysis packages.
**Figure S21** Principal component analysis (PCA) of RNA‐Seq expression across each of the 27 data sets.
**Figure S22** Volcano plots showing differential mRNA expression within various dataset comparisons.
**Figure S23** Venn diagrams showing the number of differentially expressed genes within (a) inflorescence, (b) leaf, and (c) root with statistically significant fold‐changes in mRNA expression between Col‐0 wild type (WT) compared with *VvCEB1*_*opt*,_‐overexpressing line #26 *35S::3xHA* empty‐vector control (EV) compared with *VvCEB1*_*opt*_‐overexpressing line #26, Col‐0 WT compared with EV, and all possible combinations of the pairs of genotypes.
**Figure S24** The consensus set of 227 genes with increased transcript abundance showed enrichment for gene ontology (GO) terms involved in several biological processes.
**Figure S25** Network analysis of functional associations of differentially expressed genes within inflorescences.
**Figure S26** Network analysis of functional associations of differentially expressed genes within leaves.
**Figure S27** Network analysis of functional associations of differentially expressed genes within roots.

**Table S1** List of differentially expressed genes in inflorescences, leaves, and roots following Venn Diagram analysis.
**Table S2** List of differentially expressed genes in inflorescences.
**Table S3** List of differentially expressed genes in leaves.
**Table S4** List of differentially expressed genes in roots.
**Table S5** List of network‐connected genes in inflorescences, leaves, and roots following Venn diagram analysis.
**Table S6** List of network‐connected genes in inflorescences.
**Table S7** List of network‐connected genes in leaves.
**Table S8** List of network‐connected genes in roots.
