# Supplementary material for: A Vitis vinifera basic helix–loop–helix transcription factor enhances plant cell size, vegetative biomass and reproductive yield
Source: Plant Biotechnol J. 2018 Apr 16;16(9):1595–615. doi: 10.1111/pbi.12898 (PMC6096725; doi:10.1111/pbi.12898)
Supplement: Supplementary file 1 — Figure S1 Subcellular localization of codon‐optimized VvCEB1–sGFP fusion protein in A. thaliana. Figure S2 Characterization of the wild‐type, 35S::3xHA empty‐vector control line and VvCEB1 opt‐overexpressing Arabidopsis lines. Figure S3 Seed germination rates of wild‐type, 35S::3xHA empty‐vector control line, and VvCEB1 opt‐overexpressing Arabidopsis lines. Figure S4 VvCEB1 opt overexpression in Arabidopsis increases shoot biomass. Figure S5 VvCEB1 opt ‐overexpressing Arabidopsis plants exhibit increased leaf teeth number and height along the leaf margin. Figure S6 VvCEB1 opt ‐overexpressing Arabidopsis plants exhibit hypocotyls with decreased length and increased width. Figure S7 VvCEB1 opt ‐overexpressing Arabidopsis plants exhibit increased root biomass. Figure S8 VvCEB1 opt ‐overexpression in Arabidopsis plants increases root cell size. Figure S9 VvCEB1 opt ‐overexpressing Arabidopsis plants have increased leaf thickness and cell size, but lack increased ploidy. Figure S10 VvCEB1 opt ‐overexpressing Arabidopsis plants exhibit increased chlorophyll contents on a per leaf or per plant basis. Figure S11 VvCEB1 opt ‐overexpressing Arabidopsis plants exhibit decreased cell wall thickness. Figure S12 VvCEB1 opt overexpression alters the concentration of Ca, K, P, S, and Mo in Arabidopsis leaves. Figure S13 VvCEB1 opt ‐overexpressing Arabidopsis plants exhibit increased sepal number and flower width. Figure S14 VvCEB1 opt ‐overexpressing Arabidopsis plants exhibit increased primary inflorescence stem thickness by increasing cell size. Figure S15 VvCEB1 opt ‐overexpressing Arabidopsis plants exhibit delayed bolting under half‐day and long‐day conditions. Figure S16 VvCEB1 opt ‐overexpressing Arabidopsis plants have increased soluble sugar and decreased starch contents. Figure S17 VvCEB1 opt ‐overexpressing Arabidopsis plants have increased lateral leaf primordia within the shoot apical meristem (SAM). [file PBI-16-1595-s003.pdf]

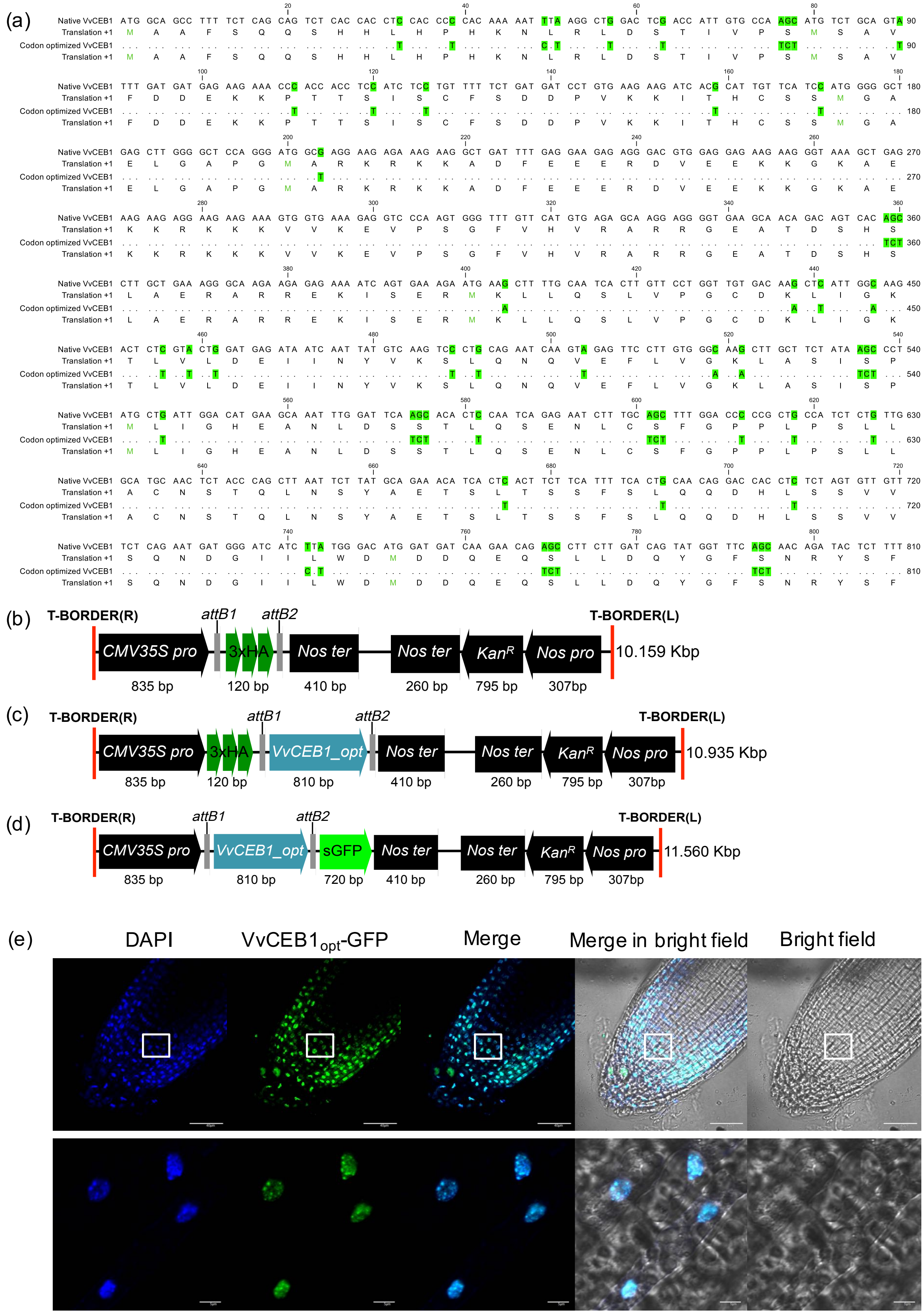

Figure S1. Subcellular localization of codon-optimized VvCEB1-sGFP fusion protein in *A. thaliana*. (a) Sequence alignment of a native *Vv*Ceb1 sequence to codon-optimized *VvCeb1* (*Vv*Ceb1$_{opt}$) for ectopic overexpression in *Arabidopsis*. Green shading or dots in codon-optimized *Vv*Ceb1 (lower line) indicate the modified or unmodified nucleotides relative native *Vv*Ceb1, respectively. Schematic representation of binary vector constructs used in this study. (b) The codon-optimized sequence of *Vv*Ceb1 was synthesized and cloned into the ImpGWB415 vector containing the CaMV *35S* promoter for transformation of *Arabidopsis*. (c) The synthesized 3xHA tag was cloned into the ImpGWB402 vector and

transformed into *Arabidopsis* for the *35S::3xHA* empty-vector control. (d) The *Vv*Ceb1$_{opt}$ fragment was cloned into the ImpGWB405 vector containing the CaMV *35S* promoter and C-terminal synthetic green fluorescent protein (sGFP) to study subcellular localization. Kanamycin (Kan^{R}) was used as the selectable marker. T-border (R) and T-border (L) indicate T-DNA right border and T-DNA left borders, respectively. (e) Nuclear localization of the VvCEB1$_{opt}$-sGFP fusion protein. The *35S::VvCEB1$_{opt}$-sGFP* construct was transformed into *Arabidopsis*. Seven-day-old seedlings (T_1) were used to analyze subcellular localization. Images in the lower panel correspond to magnification of the regions indicated by the white squares in the upper panel. Scale bars, 40 μm (top panels) and 5 μm (bottom panels).

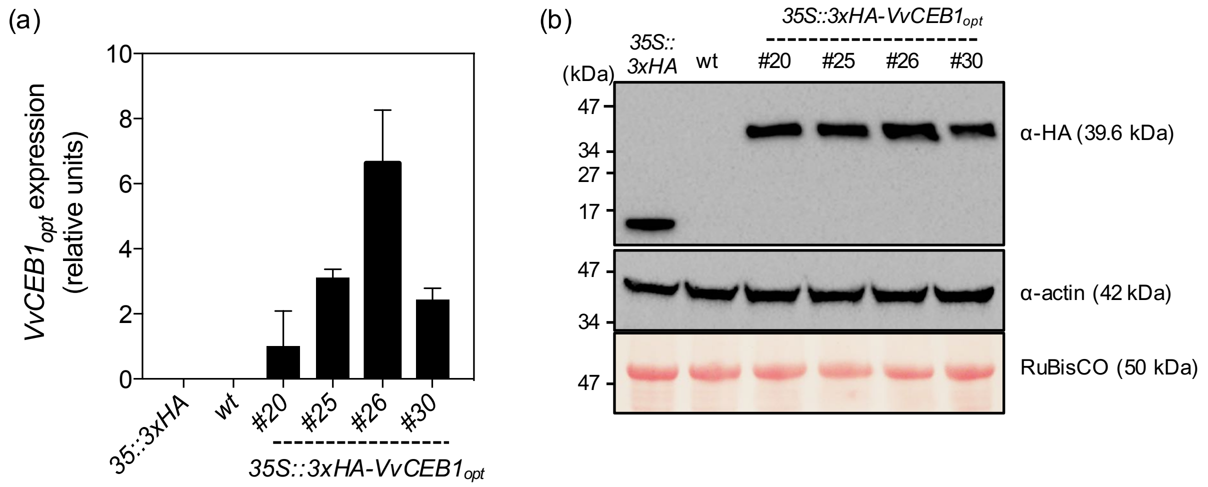

Figure S2. Characterization of the wild-type, 35S*::3xHA* empty-vector control line and *VvCEB1*$_{opt}$-overexpressing *Arabidopsis* lines. (a) Quantitative real-time PCR analysis of *Vv*Ceb1$_{opt}$ transcript abundance in *35S::3xHA* empty-vector control lines, wild-type *A. thaliana* ecotype Col-0, and four independent *VvCEB1*$_{opt}$-overexpressing lines (#20, #25, #26, and #30). Transcript levels of VvCeb1$_{opt}$ in four different lines were quantified using *TIP41-like* (AT4G34270) expression as a normalization standard. Values represent means ±s.d. of three biological replicates. (b) Immunoblot analysis with anti-HA antibody (clone 3F10, Roche Applied Science, Indianapolis, IN, USA) performed to measure protein abundance within the *3xHA-VvCEB1*$_{opt}$ transgenic lines. Immunodetection of actin and Ponceau S staining of RuBisCO were used as loading controls.

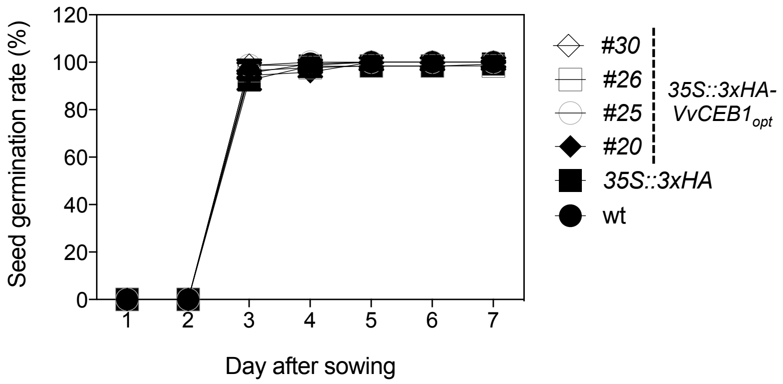

Figure S3. Seed germination rates of wild-type,35S::3xHA empty vector control, and *Vv*CEB1$_{\mathbf{opt}}$-overexpressing *Arabidopsis* lines. Seeds were germinated and grown on MS agar medium for 7 days under a 16-h photoperiod. Seed germination rates of four independent *VvCEB1$_{opt}$*-overexpressing lines (#20, #25, #26, and #30), wild-type (wt) *A. thaliana* ecotype Col-0, and the *35S::3xHA* empty-vector control line were scored for 7 days (*n* = 3).

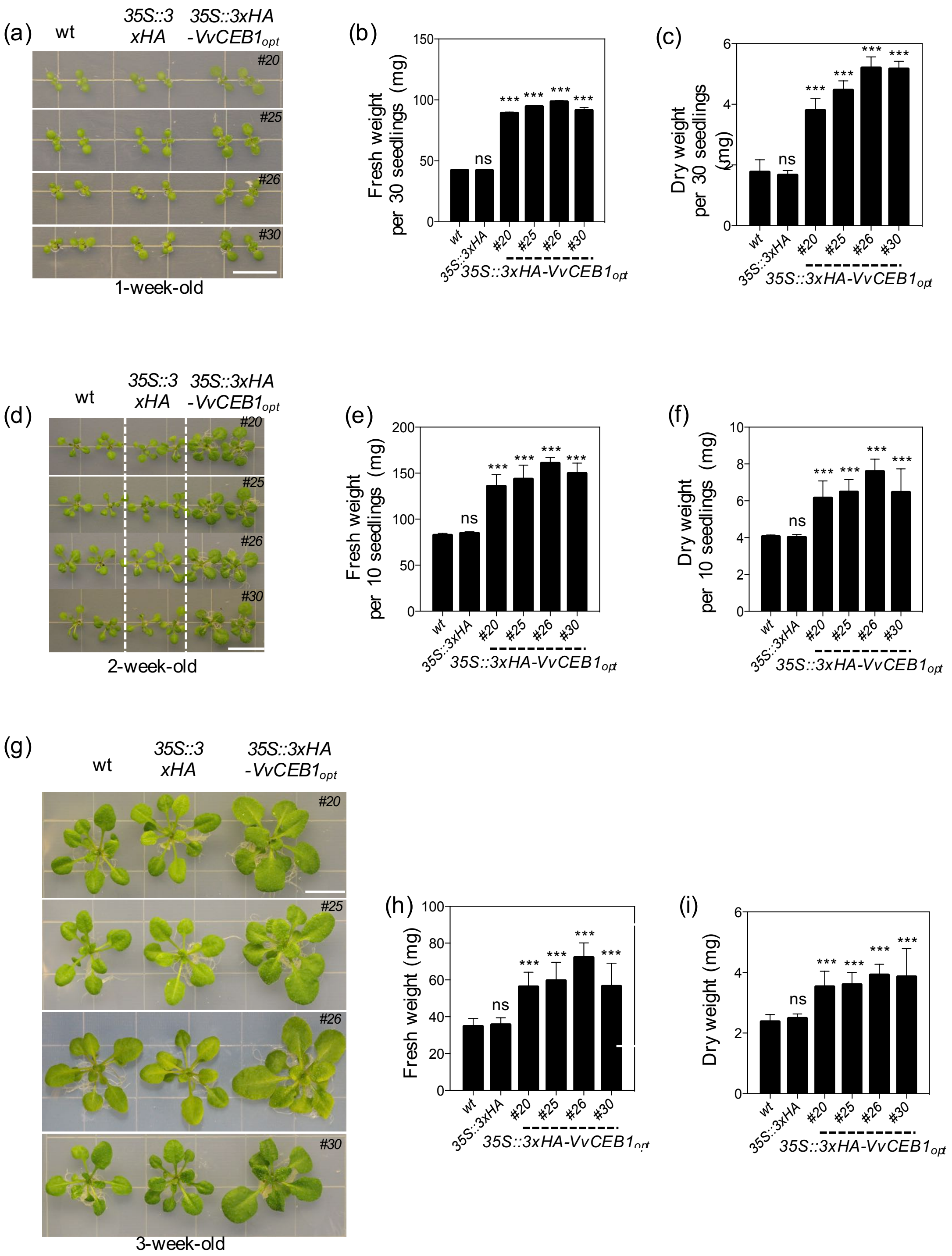

Figure S4. *Vv*CEB1$_{opt}$ overexpression in *Arabidopsis* increases shoot biomass. Seeds were germinated and grown on MS agar medium for 7 days for quantification of the seedling biomass. (a) Representative images of one-week-old seedlings of *VvCEB1$_{opt}$*- overexpressing lines, and the Col-0 wild type, and the *35S::3xHA* empty-vector control lines. Scale bar, 1 cm. (b) Quantification of fresh weight of 30 seedlings (*n* = 3 replicates). (c) Dry weight of 30 seedlings (*n* = 3 replicates). Seeds were germinated and grown on MS agar medium for 2

weeks for quantification of shoot biomass. (d) Representative images of two-week-old seedlings of four independent *VvCEB1*$_{opt}$-overexpressing lines, Col-0 wild type, and the *35S::3xHA* empty-vector control line. Scale bar, 1 cm. (e) Quantification of fresh weight of 10 seedlings (n = 3 replicates). (f) Dry weight of 10 seedlings (n = 3 replicates). Seeds were germinated and grown on MS agar medium for 3 weeks for quantification of plant biomass. (g) Representative images of three-week-old seedlings of four independent *VvCEB1*$_{opt}$-overexpressing lines, Col-0 wild type, and the *35S::3xHA* empty-vector control line. Scale bar, 1 cm. (h) Quantification of fresh weight per plant (n = 10). (i) Dry weight of three-week-old seedlings (n = 10). Values represent means ±s.d., ns = non-significant, ***p < 0.001, One-way ANOVA with Dunnett's multiple comparison test.

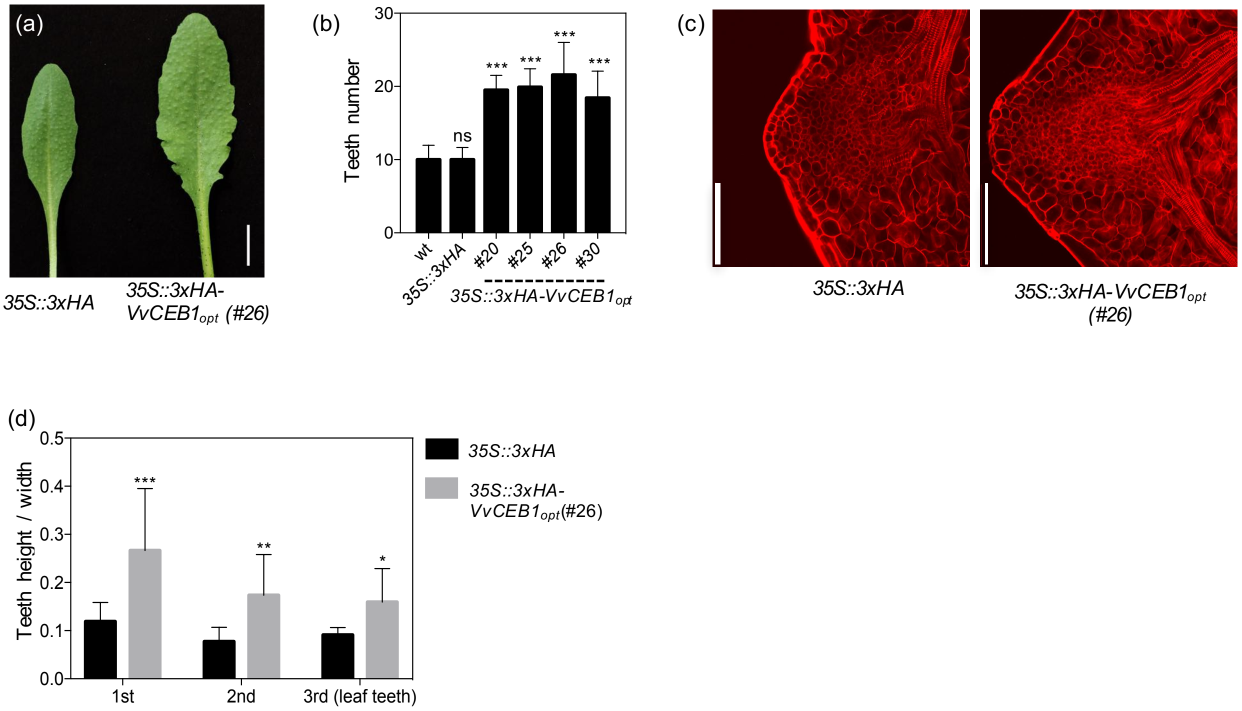

Figure S5. *Vv*CEB1$_{opt}$-overexpressing *Arabidopsis* plants exhibit increased leaf teeth number and height along the leaf margin. Seeds of Col-0 wild-type (wt), the *35S::3xHA* empty-vector control, and *VvCEB1$_{opt}$*-overexpressing lines (#20, #25, #26, and #30), were germinated and grown in soil mix for 4 weeks under a 12-h photoperiod. (a) Representative images of fifth leaves of *VvCEB1$_{opt}$*-overexpressing line and the *35S::3xHA* empty-vector control line. Scale bar, 1 cm. (b) Quantification of teeth number per leaf (n = 12) (c) Confocal laser scanning images of 1st leaf teeth from petiole. Fifth leaves of four-week-old plants were stained with propidium iodide (PI). Scale bar, 100 µm. (d) Quantification of leaf teeth height/length ratio (n = 14). Values represents means ±s.d., ns = non-significant, *p < 0.05, **p < 0.01, and ***p < 0.001, One-way ANOVA with Dunnett's multiple comparison test.

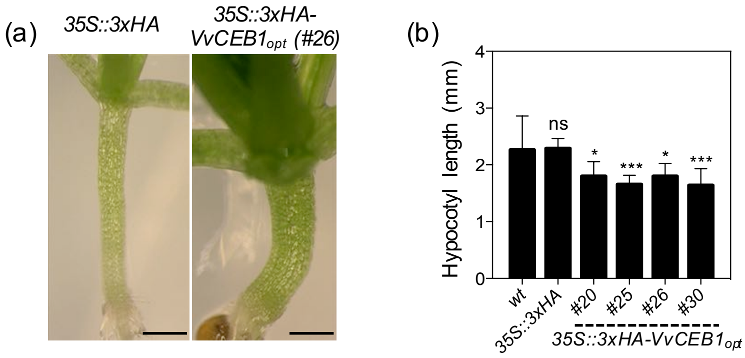

Figure S6. *Vv*CEB1$_{opt}$ overexpressing *Arabidopsis* plants exhibit hypocotyls with decreased length and increased width. Seeds of Col-0 wild-type, the *35S::3xHA* empty-vector control line, and four *VvCEB1$_{opt}$*-overexpressing lines were germinated and grown vertically on half-strength Murashige and Skoog (MS) agar medium for 14 days under a 16-h photoperiod. (a) Images of hypocotyls from VvCEB1$_{opt}$-overexpressing line (#26) and the *35S::3xHA* empty-vector control. Scale bar, 0.5 mm. (b) Comparison of hypocotyl length (n = 10). (c) Hypocotyl width (n = 10). Values represent means ±s.d., ns = non-significant, *$p < 0.05$, **$p < 0.01$, and ***$p < 0.001$, One-way ANOVA with Dunnett's multiple comparison test.

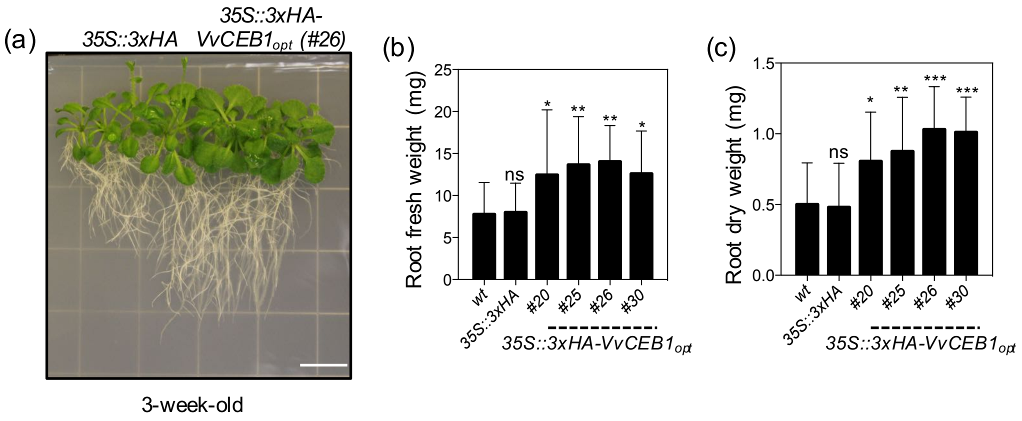

Figure S7. *Vv*CEB1$_{opt}$-overexpressing *Arabidopsis* plants exhibit increased root biomass. Seedlings were grown vertically on half-strength MS medium for 3 weeks under a 16-h photoperiod (16 hours of light per day). (a) Representative images of root biomass of *35S::3xHA-VvCEB1*$_{opt}$ line (#26), and the *35S::3xHA* empty-vector control line. Scale bar, 1 cm. (b) Quantification of root fresh weight of three-week-old seedlings (n = 20). (c) Root dry weight of three-week-old seedlings (n = 20). Values represent means ±s.d., ns = non-significant, *p < 0.05, **p < 0.01, and ***p < 0.001, One-way ANOVA with Dunnett's multiple comparison test.

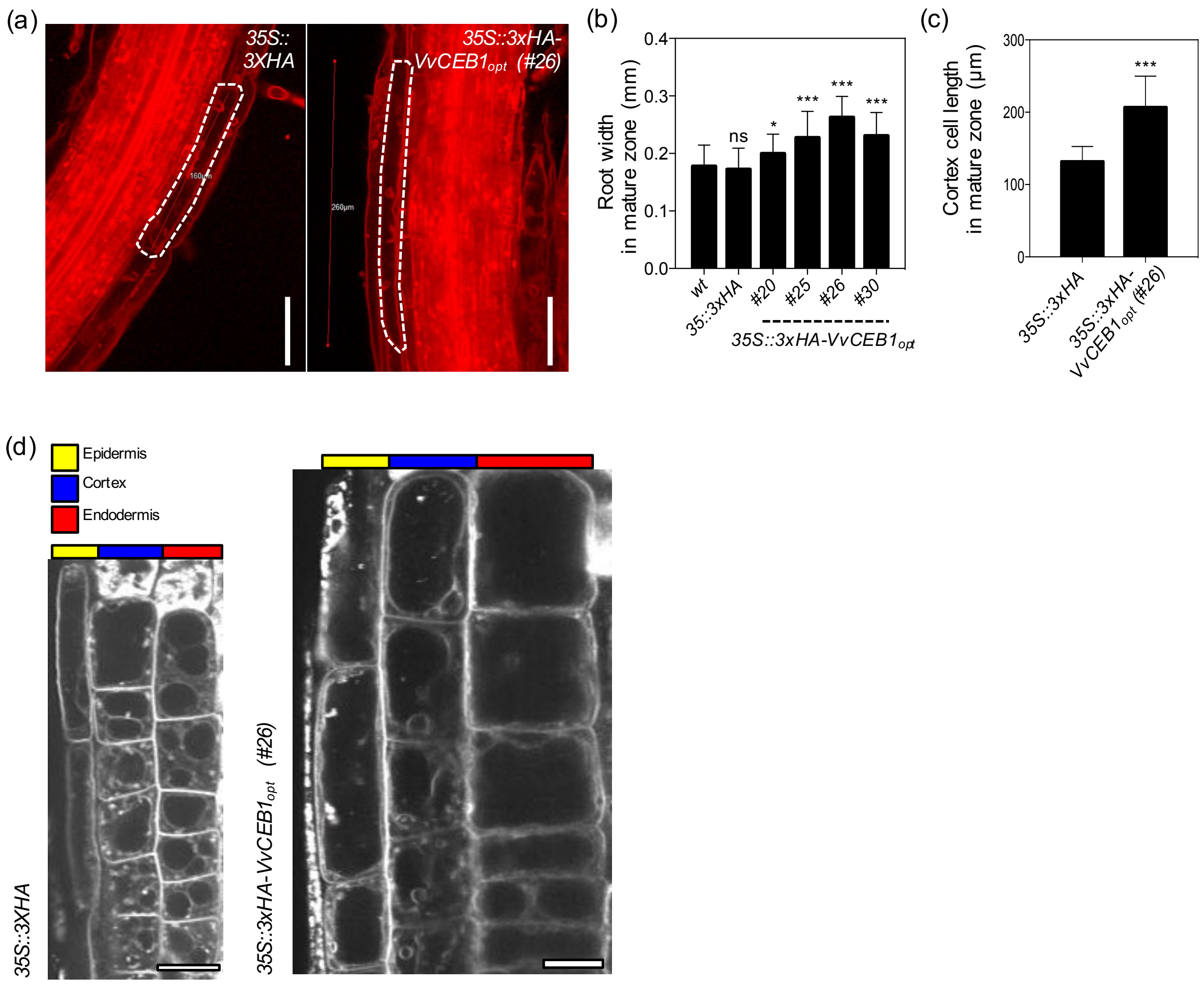

Figure S8. *Vv*CEB1$_{opt}$-overexpression in *Arabidopsis* plants increases root cell size. Seeds of Col-0 wild-type (wt), the *35S::3xHA* empty-vector control line, and the four *VvCEB1$_{opt}$*-overexpressing lines, were germinated and grown vertically on half-strength MS agar medium for 14 days under a 16-h photoperiod (16 hours of light per day). (a) Confocal laser scanning images of root cells stained with FM4-64 (2 h) and PI in mature zone. White broken lines indicate the outlines of representative cortical cells. Scale bar, 60 µm. (b) Quantification of primary root width in the maturation zone (n = 30). (c) Quantification of cortical root cell length in mature zone (n = 60). (d) Images of cells and vacuoles in basal meristem zone stained with FM4-64 (5 h) captured by confocal laser scanning microscopy. Yellow, blue, and red lines indicate the epidermis, cortex, and endodermis, respectively. Scale bar, 10 µm. Values represent means ±s.d., ns = non-significant, *p < 0.05, and ***p < 0.001, One-way ANOVA with Dunnett's multiple comparison test (b) and Student's t-test (c).

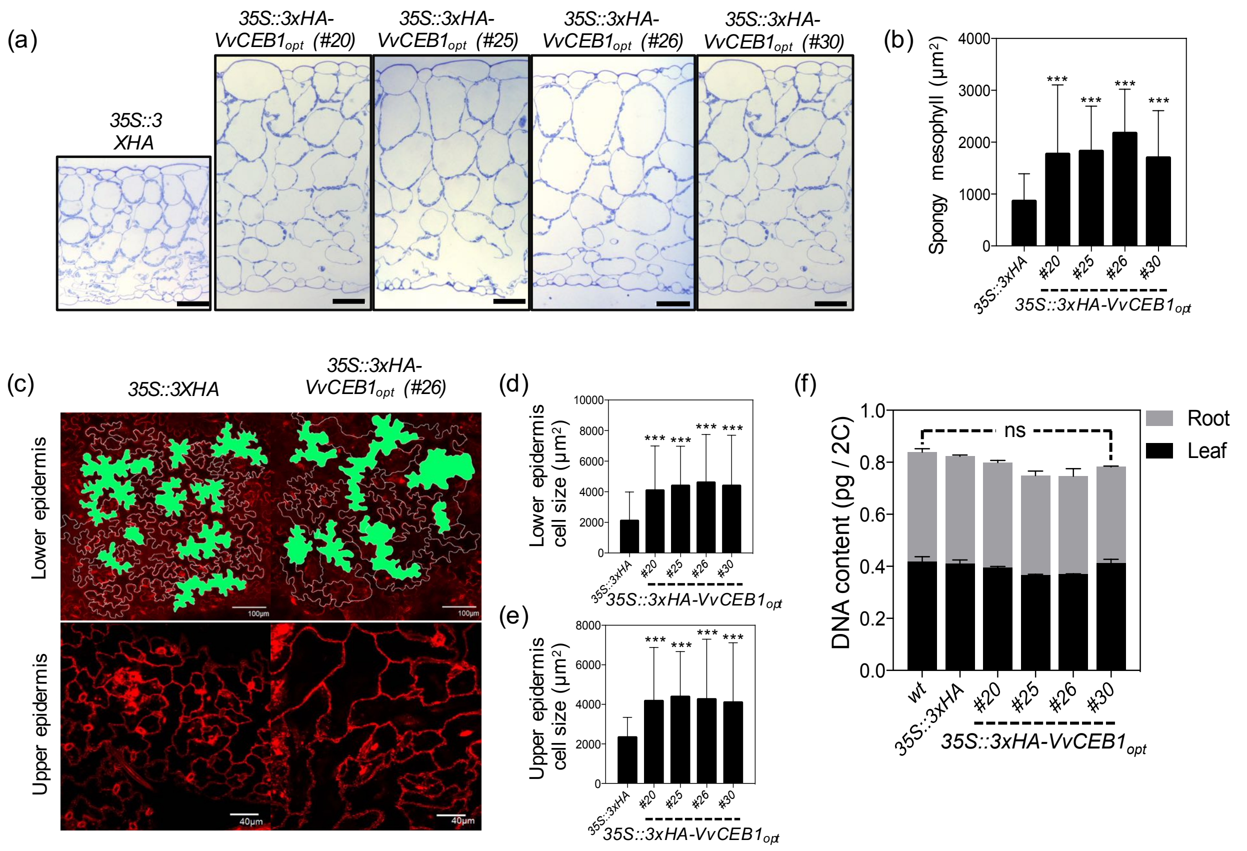

Figure S9. *Vv*CEB1$_{opt}$-overexpressing *Arabidopsis* plants exhibit increased leaf thickness and cell size, but lack increased ploidy. Seeds of the *35S::3xHA* empty-vector control and the VvCEB1$_{opt}$-overexpressing lines (#20, #25, #26, and #30) were germinated and grown in soil mix for 4 weeks under a 12-h photoperiod. (a) Transverse sections of leaves from four *Vv*CEB1$_{opt}$-overexpressing lines and the *35S::3xHA* empty-vector control line stained with toluidine blue O. Scale bar, 100 μm. (b) Quantification of the size of spongy mesophyll cells (*n* = 350). (c) Representative images of tangential leaf sections from VvCEB1$_{opt}$-overexpressing line (#26) and the *35S::3xHA* empty-vector control. Upper and lower epidermis cell layers of four-week-old plants were stained with propidium iodide (PI). Images were captured using laser scanning confocal microscopy. Green shading indicates representative areas of individual cells of the lower epidermis. Scale bars, 100 μm (upper panels) and 40 μm (lower panels). (d) Quantification of the size of cells in the lower epidermis (*n* = 90). (e) Quantification of the size of cells in the upper epidermis (*n* = 50). (f) Roots and leaves of three-week-old *Vv*CEB1$_{opt}$-overexpressing lines, Col-0 wild-type, and the *35S::3xHA* empty-vector control line were separately sampled and DNA contents were analyzed by flow cytometry (*n* = 3 replicates). Values represents means ±s.d., ns = non-significant, $**p < 0.01$, and $***p < 0.001$, One-way ANOVA with Dunnett's multiple comparison test.

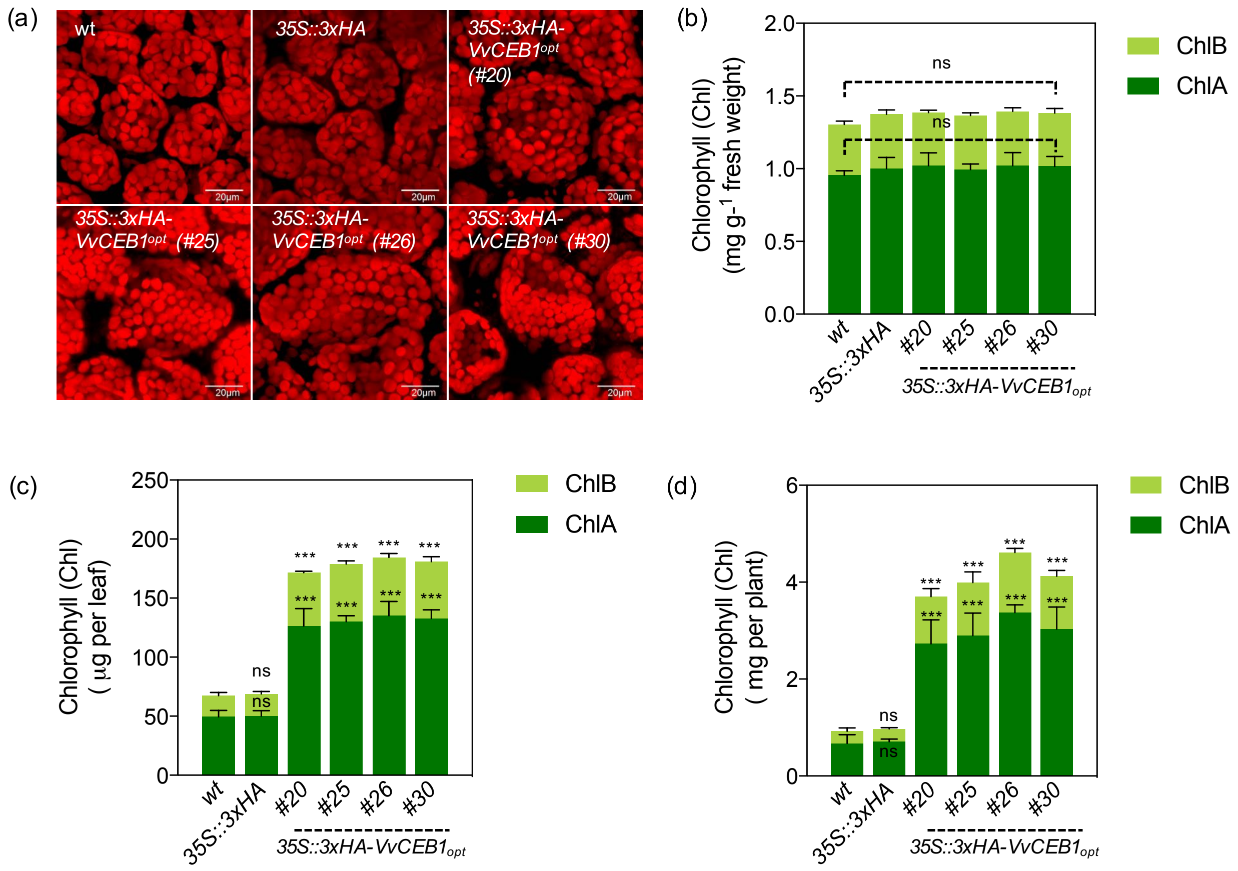

Figure S10. *Vv*CEB1$_{opt}$-overexpressing *Arabidopsis* plants have increased chlorophyll contents on a per leaf or per plant basis. Seeds of wild-type *A. thaliana* ecotype Col-0, the *35S::3xHA* empty-vector control line, and the four *VvCEB1$_{opt}$*-overexpressing lines were germinated and grown in soil mix for 4 weeks under a 12-h photoperiod (12 hours of light per day). (a) Confocal laser scanning images of palisade mesophyll cells and chloroplasts of *VvCEB1$_{opt}$*-overexpressing lines and control lines. Chlorophyll auto-fluorescence in the leaf palisade mesophyll cells is shown in red. The 3D-projection was generated by combining a Z-stack of images. Scale bar, 20 µm. Fifth fully expanded true leaves were sampled and measured for chlorophyll contents (n = 4 replicates). (b) Chlorophyll contents per gram fresh weight. (c) Chlorophyll contents per leaf. (d) Chlorophyll contents per plant. Values represent means ± s.d., ns = non-significant, ***p < 0.001, One-way ANOVA with Dunnett's multiple comparison test.

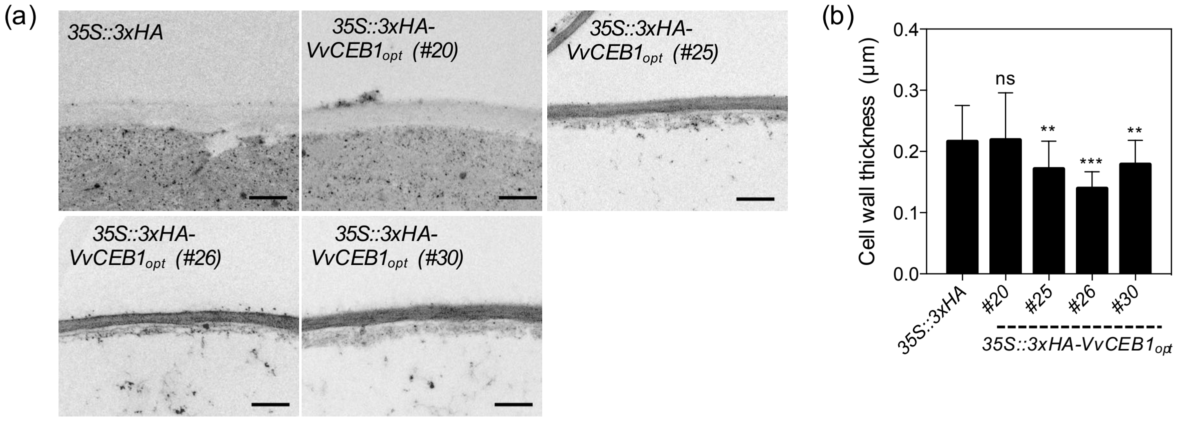

Figure S11. *Vv*CEB1$_{opt}$-overexpressing *Arabidopsis* plants exhibit decreased cell wall thickness. (a) Representative cell wall images of the *35S::3xHA* empty-vector control line and the four VvCEB1$_{opt}$-overexpressing lines and the from four-week-old, soil mix-grown plants grown under a 12-h photoperiod. Images were captured by transmission electron microscopy. Scale bar, 0.5 µm. (b) Quantification of cell wall thickness (n = 45). Values represents means ±s.d., ns = non-significant, **p < 0.01, and ***p < 0.001, One-way ANOVA with Dunnett's multiple comparison test.

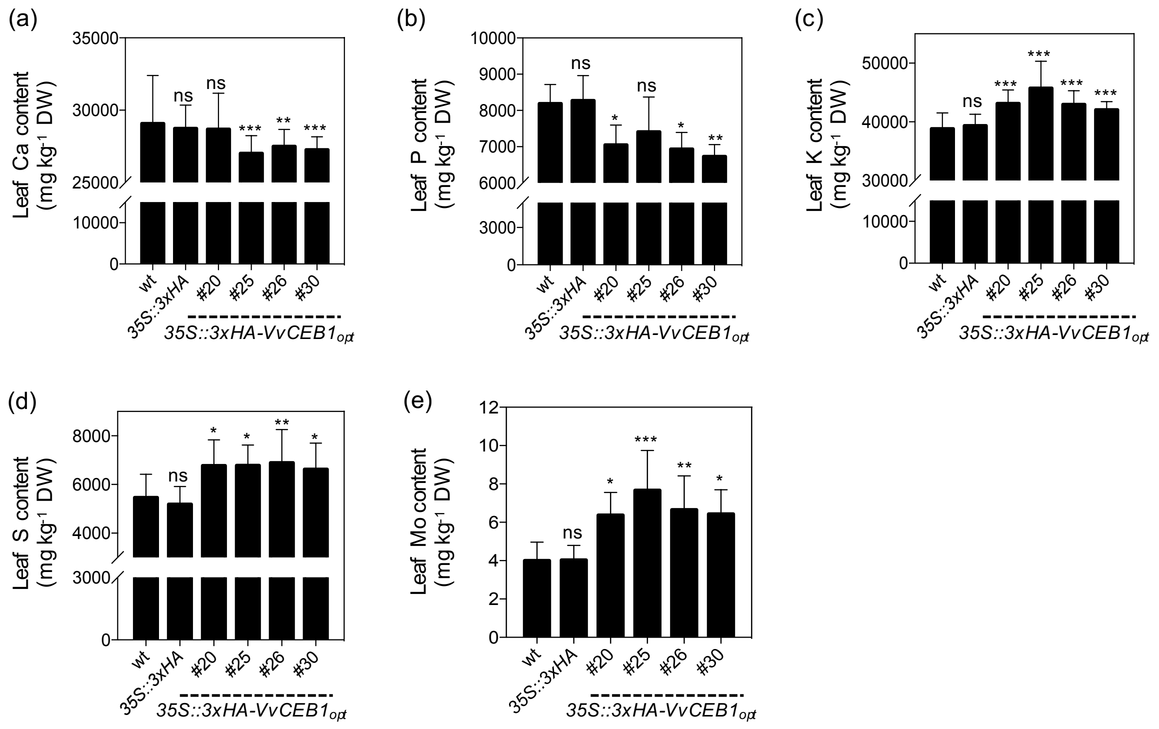

Figure S12. *Vv*CEB1$_{opt}$ overexpression alters the concentration of Ca, K, P, S, and Mo in *Arabidopsis* leaves. Determine the concentrations of inorganic ions in wild-type plants, *35S::3xHA* empty-vector control line, and *VvCEB1$_{opt}$*-overexpressing lines. (a) Calcium, (b) Potassium, (c) Phosphorus, (d) Sulfur, and (e) Molybdenum content on a dry weight basis (n = 6). Values represent means ±s.d., ns = non-significant, *$p < 0.05$, **$p < 0.01$, and ***$p < 0.001$, one-way ANOVA with Dunnett's multiple comparison test.

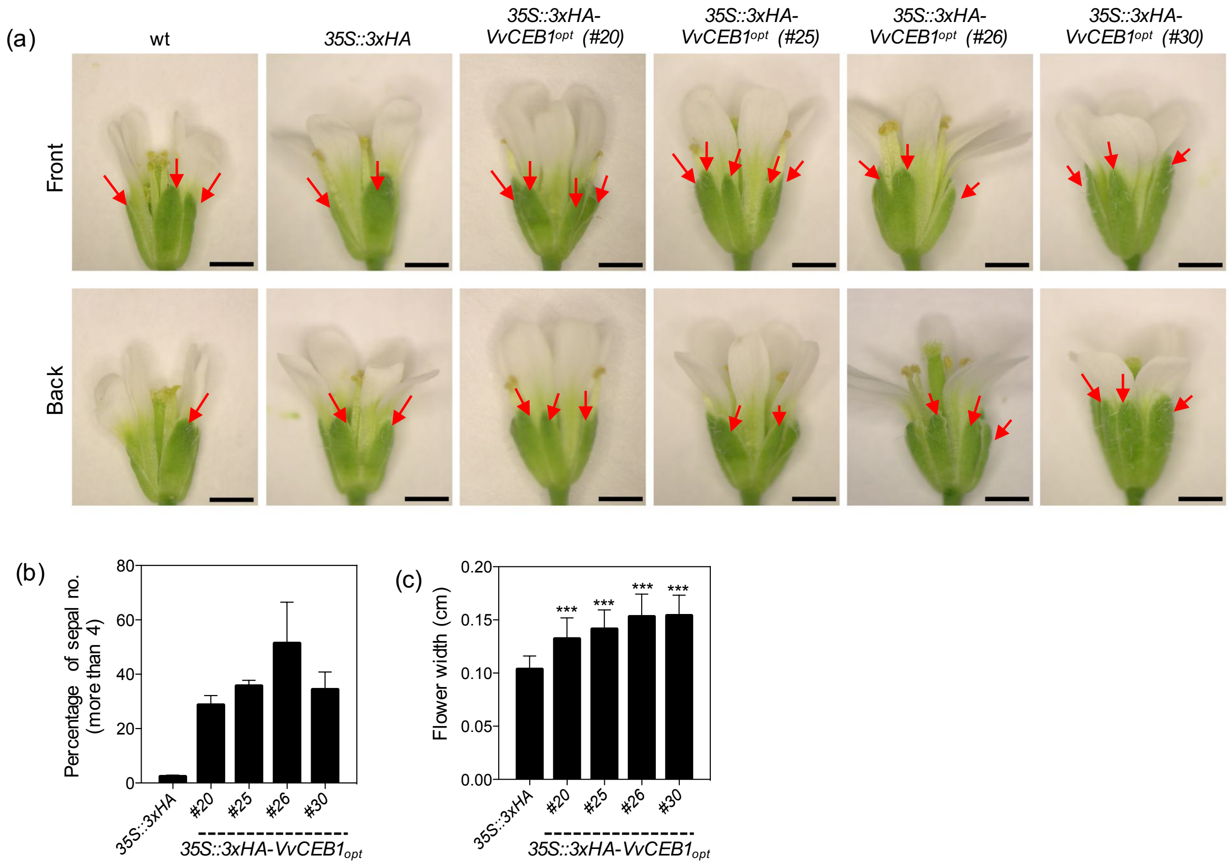

Figure S13. *Vv*CEB1$_{opt}$-overexpressing *Arabidopsis* plants increase sepal number in flowers and flower width. (a) Representative flower images of Col-0 wild type (wt), the *35S::3xHA* empty-vector control line, and the four *VvCEB1$_{opt}$*-overexpressing lines,. Arrows indicate sepal number. Scale bar, 1 mm. (b) Quantification of percentage of flowers with sepal number greater than 4 (*n* = 3 replicates). (c) Quantification of flower width measured horizontally (*n* = 63). Values represent means ± s.d., ****p* < 0.001, One-way ANOVA with Dunnett's multiple comparison test.

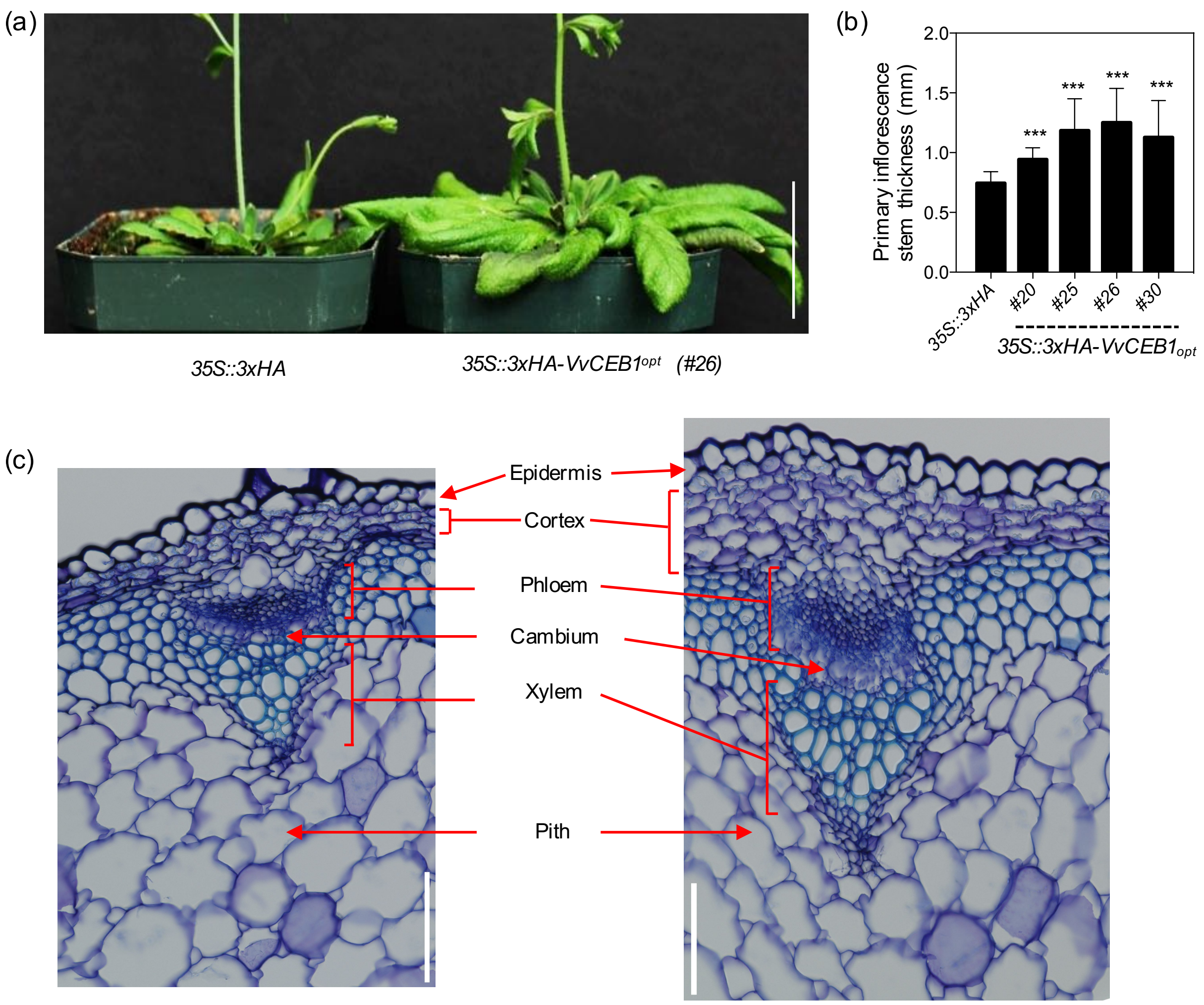

Figure S14. *Vv*CEB1$_{opt}$-overexpressing *Arabidopsis* plants exhibit increased inflorescence stem thickness by increasing cell size. Seeds of *35S::3xHA* empty-vector control line and the four *VvCEB1$_{opt}$*-overexpressing lines were germinated and grown in soil mix under a 12-h photoperiod (12 hours of light per day). (a) Representative images of the primary inflorescence stem of the *35S::3xHA* empty-vector control line and the *VvCEB1$_{opt}$*-overexpressing line (#26) at 2 weeks after bolting. Scale bar, 5 cm. (b) Quantitation of the diameter of the primary inflorescence stem (n = 30). (c) Representative images of stem cross sections. Primary inflorescence stems were stained with toluidine blue O. Scale bar, 100 µm. Values represent means ± s.d., ***p < 0.001, One-way ANOVA with Dunnett's multiple comparison test.

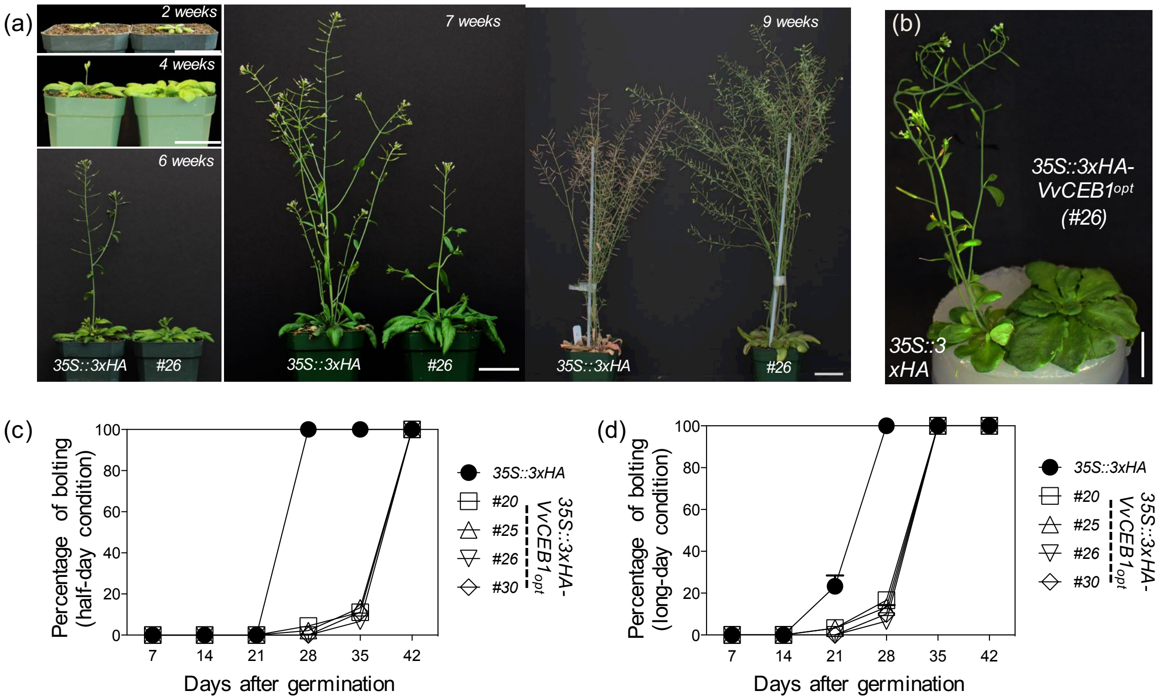

Figure S15. *Vv*CEB1$_{opt}$ overexpressing *Arabidopsis* plants exhibit delayed bolting under half-day and long-day conditions. (a) Representative images of delayed flowering and leaf senescence of the *35S::3xHA* empty-vector control line and the VvCEB1$_{opt}$-overexpressing line (#26) under half-day condition. Seeds of *35S::3xHA* (empty-vector control) line and the four independent *35S::3xHA-VvCEB1opt* lines (#20, #25, #26, and #30) were germinated and grown in soil for nine weeks under a 12-h photoperiod. Scale bar, 5 cm. (b) Representative images of delayed flowering of the *35S::3xHA* empty-vector control line and the *Vv*CEB1$_{opt}$-overexpressing line (#26) under long-day condition. Seeds of the *35S::3xHA* empty-vector control line and the four independent *Vv*CEB1$_{opt}$-overexpressing lines were germinated and grown on MS agar medium under a 16-h photoperiod. Scale bar, 5 cm. Quantification of flowering time under (c) half-day and (d) long-day conditions (*n* = 3 replicates).

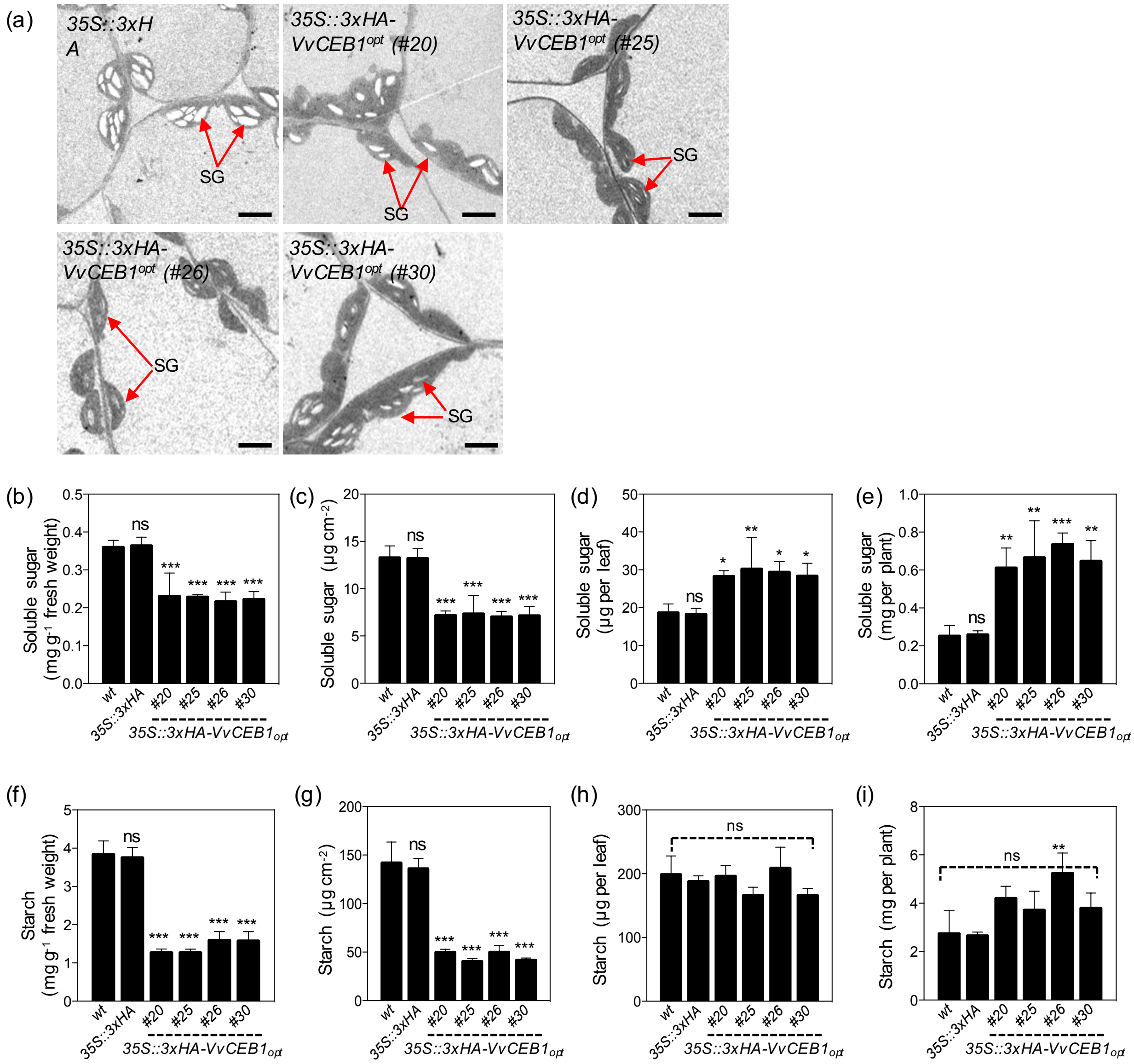

Figure S16. *Vv*CEB1$_{opt}$-overexpressing *Arabidopsis* plants have increased soluble sugar and decreased starch contents. Seeds of wild-type (wt) *A. thaliana* ecotype Col-0, the *35S::3xHA* empty-vector control line, and the four *Vv*CEB1$_{opt}$-overexpressing lines were germinated and grown in soilless growth medium for 4 weeks under a 12-h photoperiod. Fifth fully expanded true leaves were sampled at noon for carbohydrate assays. (a) Representative images of chloroplasts in *Vv*CEB1$_{opt}$-overexpressing lines, Col-0 wild type, and *35S::3xHA* empty-vector control lines. Images were captured by transmission electron microscopy. Arrows indicate starch granules (SG). Scale bar, 5 µm. Soluble sugar contents in leaves (*n* = 4 replicates). (b) Soluble sugar contents per gram fresh weight. (c) Soluble sugar contents per leaf area (cm^2). (d) Soluble sugar contents per leaf. (e) Soluble sugar contents per plant. Starch contents in leaves (*n* = 4 replicates). (f) Starch contents per gram fresh weight. (g) Starch contents per unit leaf area (cm^2). (h) Starch contents per leaf. (i) Starch contents per plant.

Values represent means ±s.d., ns = non-significant, *$p < 0.05$, **$p < 0.01$, and ***$p < 0.001$, One-way ANOVA with Dunnett's multiple comparison test.

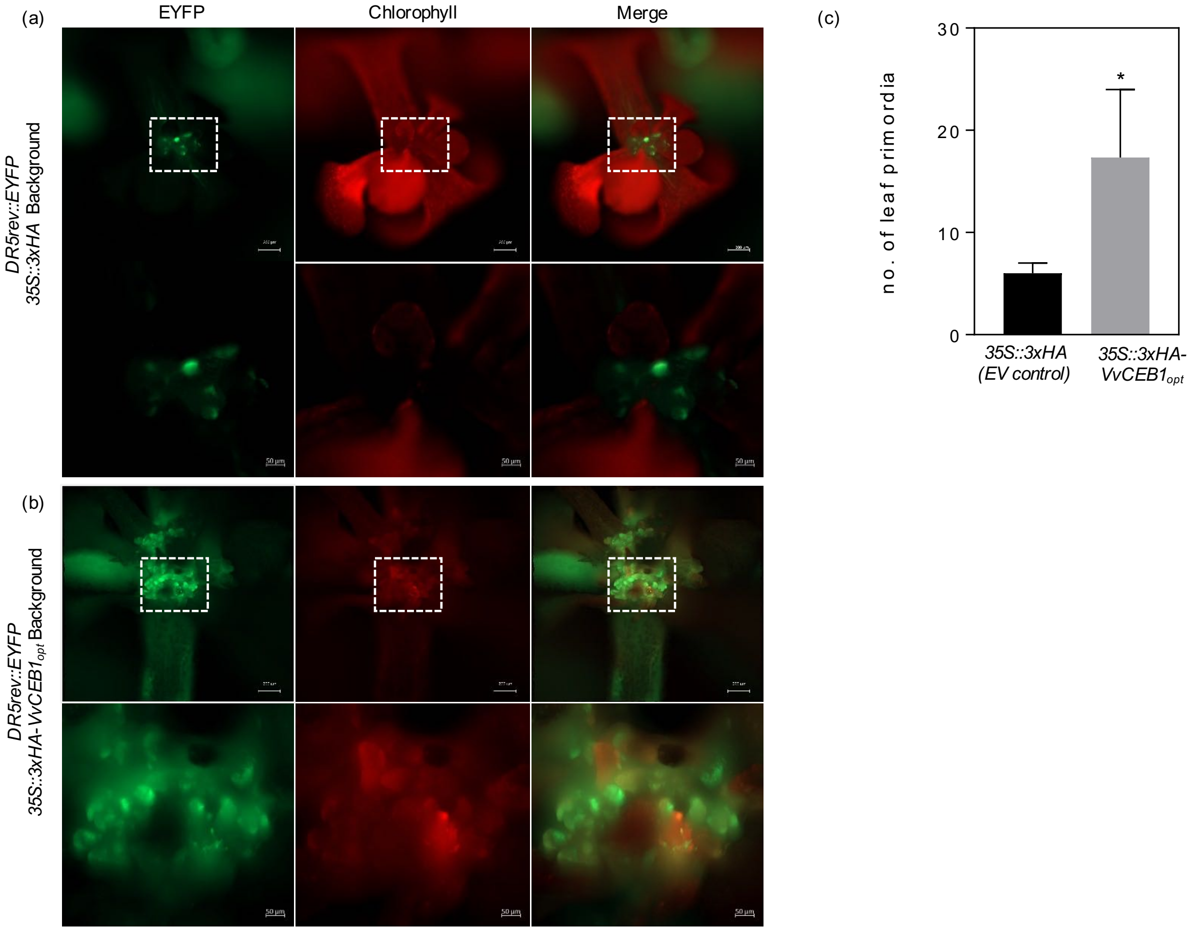

Figure S17. VvCEB1opt overexpression increases leaf primordia number in the shoot apical meristem (SAM) in Arabidopsis. (a), Expression of DR5rev::EYFP in the SAM region of empty vector (EV) control and (b) Ox-VvCEB1opt (#26) transgenic background lines (Size bars = 200 µm or 50 µm (insets)). (c) Quantification of leaf primordia number ($n = 3$). Values represents means ±s.d. and *p < 0.05. Student's t-test.
