# Supplementary material for: A Vitis vinifera basic helix–loop–helix transcription factor enhances plant cell size, vegetative biomass and reproductive yield
Source: Plant Biotechnol J. 2018 Apr 16;16(9):1595–615. doi: 10.1111/pbi.12898 (PMC6096725; doi:10.1111/pbi.12898)
Supplement: Supplementary file 2 — Figure S18 Summary of total RNA‐Seq read counts per tissue type, read mapping rate, and read quality for individual samples. Figure S19 Testing for two‐fold differential expression within biological sample replicates between RNA‐Seq samples from each organ type. Figure S20 RNA‐Seq expression correlation matrix heat maps of RNA‐Seq samples generated by four different differential expression analysis packages. Figure S21 Principal component analysis (PCA) of RNA‐Seq expression across each of the 27 data sets. Figure S22 Volcano plots showing differential mRNA expression within various dataset comparisons. Figure S23 Venn diagrams showing the number of differentially expressed genes within (a) inflorescence, (b) leaf, and (c) root with statistically significant fold‐changes in mRNA expression between Col‐0 wild type (WT) compared with VvCEB1 opt,‐overexpressing line #26 35S::3xHA empty‐vector control (EV) compared with VvCEB1 opt ‐overexpressing line #26, Col‐0 WT compared with EV, and all possible combinations of the pairs of genotypes. Figure S24 The consensus set of 227 genes with increased transcript abundance showed enrichment for gene ontology (GO) terms involved in several biological processes. Figure S25 Network analysis of functional associations of differentially expressed genes within inflorescences. Figure S26 Network analysis of functional associations of differentially expressed genes within leaves. Figure S27 Network analysis of functional associations of differentially expressed genes within roots. [file PBI-16-1595-s002.pdf]

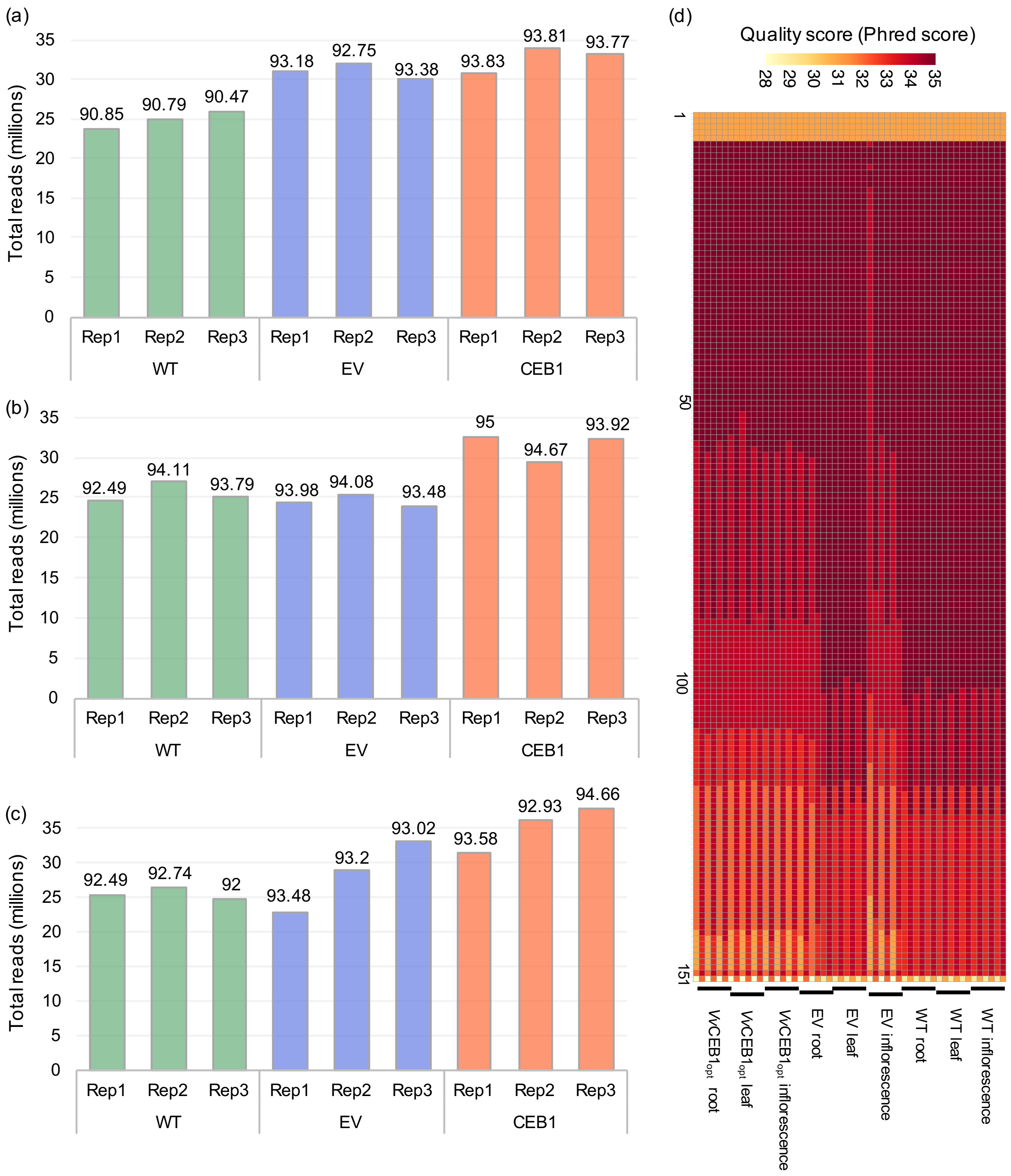
(a)
Total reads (millions)
0
5
10
15
20
25
30
35
90.85
90.79
90.47
93.18
92.75
93.38
93.83
93.81
93.77
Rep1
Rep2
Rep3
WT
EV
CEB1
(b)
Total reads (millions)
92.49
94.11
93.79
93.98
94.08
93.48
95
94.67
93.92
Rep1
Rep2
Rep3
WT
EV
CEB1
(c)
Total reads (millions)
92.49
92.74
92
93.48
93.2
93.02
93.58
92.93
94.66
Rep1
Rep2
Rep3
WT
EV
CEB1
(d)
Quality score (Phred score)
28
29
30
31
32
33
34
35
1
50
100
151
VvCEB1opt root
VvCEB1opt leaf
VvCEB1opt inflorescence
EV root
EV leaf
EV inflorescence
WT root
WT leaf
WT inflorescence

Figure S18. Summary of total RNA-Seq read counts per tissue type, read mapping rate, and read quality for individual samples. Each bar plot corresponds to one replication of Col-0 wild type (WT), the *35S::3xHA* empty-vector control (EV), and *VvCEB1*$_{opt}$-overexpressing line (#26). Each bar indicates the number of reads in each paired-end read set after trimming low-quality reads (Q ≥20) using Trimmomatic (Version 0.36). The number above each bar plot indicates the read-mapping rate (%) produced using Bowtie2. (a) RNA-Seq reads from inflorescence samples. (b) RNA-Seq reads from leaf samples. (c) RNA-Seq reads from root samples. (d) Heat map indicating read quality. The color of each cell represents the Phred quality score within the range of Phred values (scale = 28-35) reflecting the base quality of the individual reads.

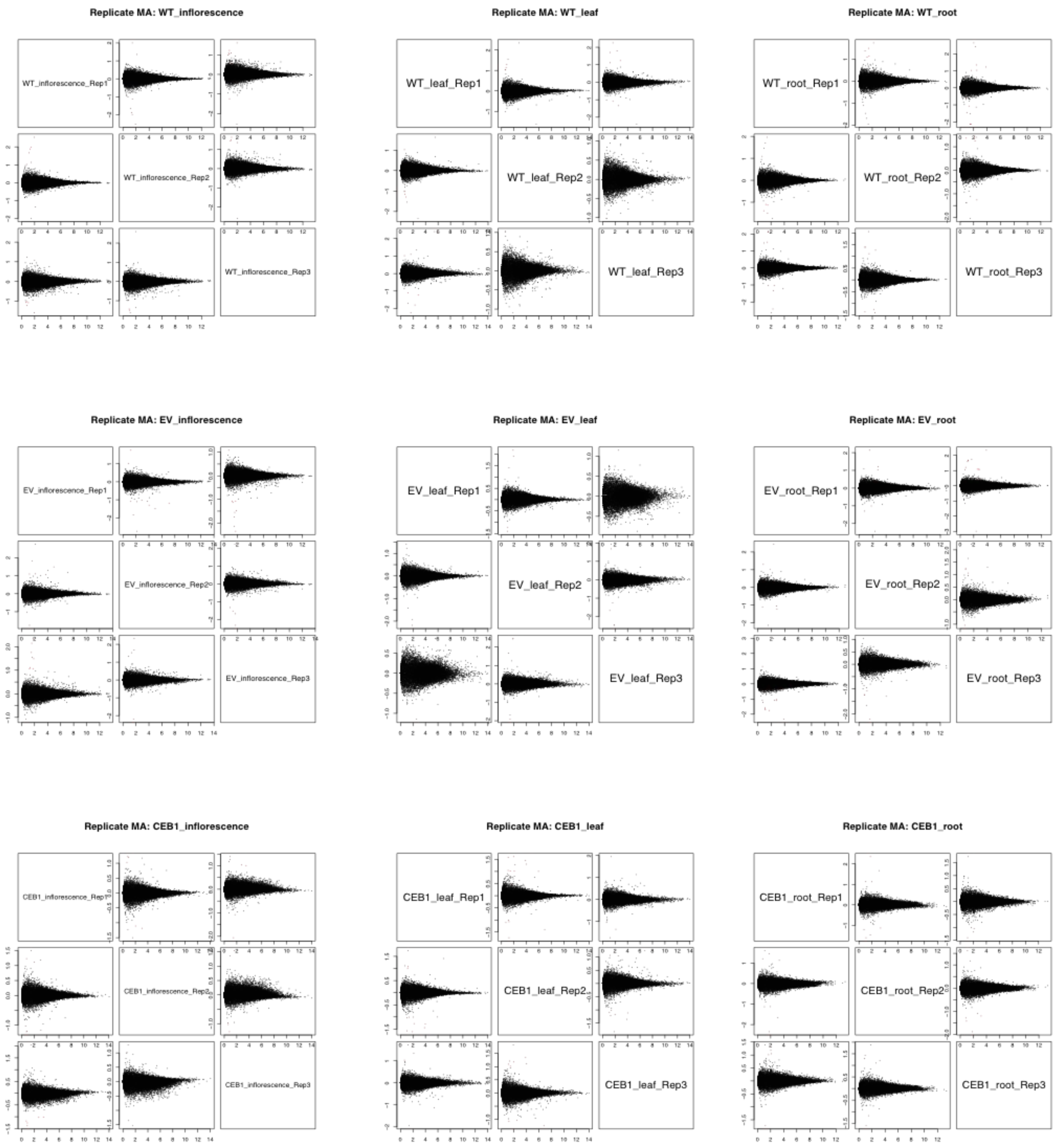

Figure S19. Testing for two-fold differential expression within biological sample replicates between RNA-Seq samples from each organ type. Each of the nine Bland-Altman (MA) plot provides a visual representation of the level of agreement between biological replicates obtained within each of the pair-wise sample comparisons generated by edgeR. Each plot depicts the relative differences identified as significantly different expression at 0.001 FDR and two-fold changes in gene expression are colored in red.

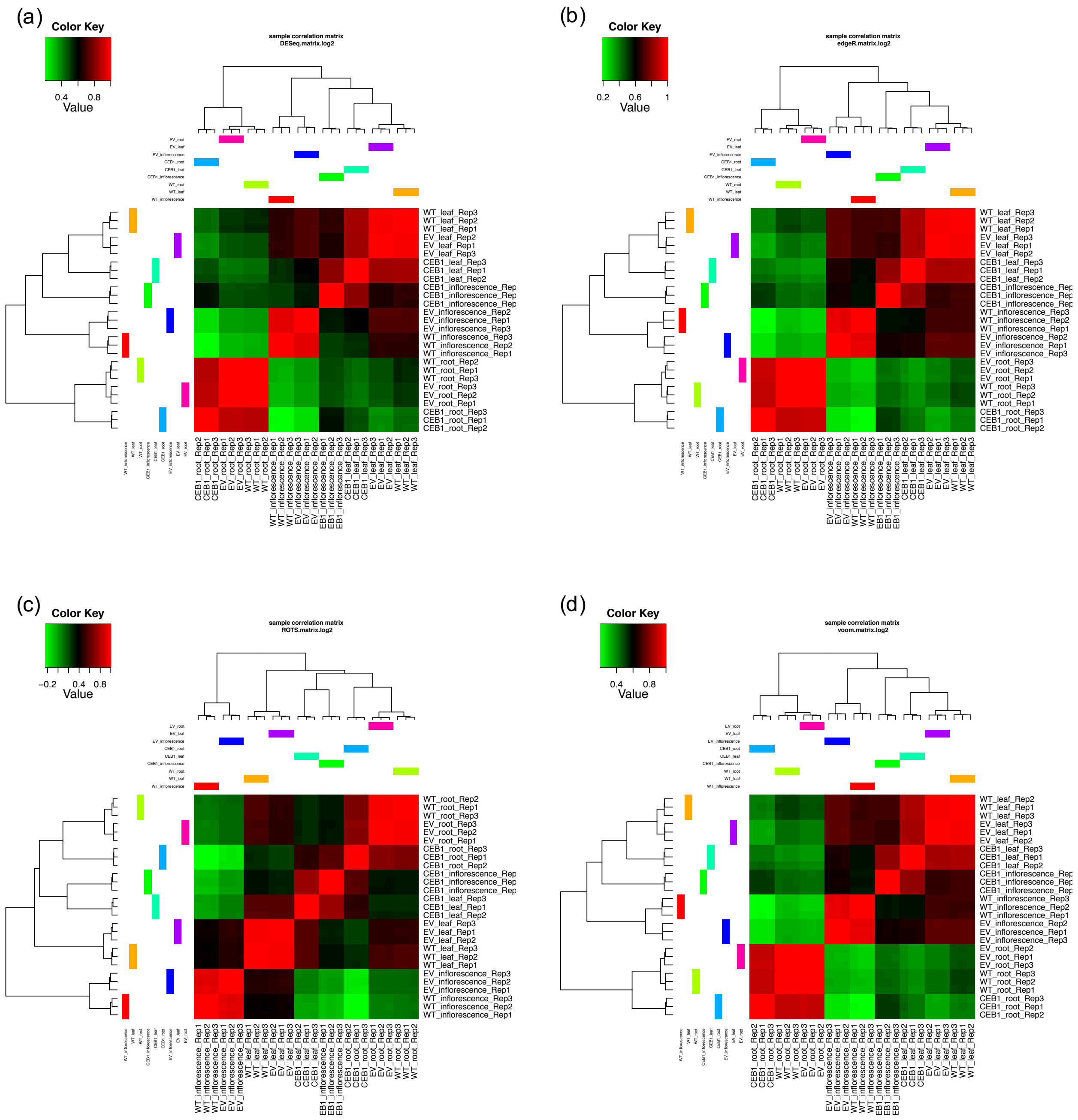

Figure S20. RNA-Seq expression correlation matrix heat maps of RNA-Seq samples generated by four different differential expression analysis packages. The FPKM values calculated by each package for each organ contrast were clustered with a pair-wise Pearson correlation coefficient calculated by each algorithm. The color key located on the top left corner of each heat map indicates the Pearson correlation coefficient score ranges obtained using each algorithm. The colored bars alongside the heat map show the relative clustering obtained for each of the samples. The clusters were generated using the complete linkage method. (a) Heat map generated using DESeq2. (b) Heat map generated using edgeR. (c) Heat map generated using ROTS. (d) Heat map generated using voom.

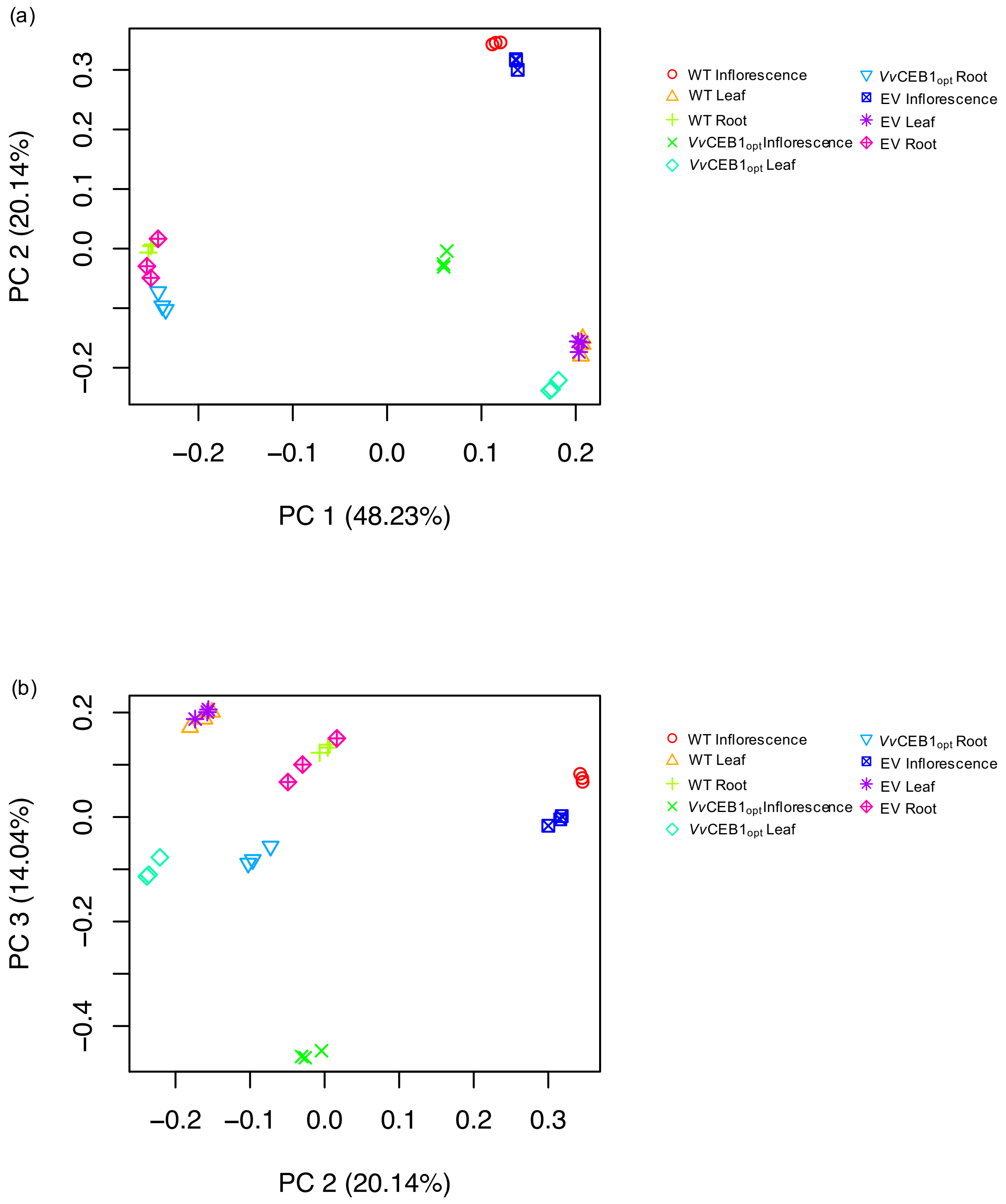

Figure S21. Principal component analysis (PCA) of RNA-Seq expression across each of the 27 data sets. Expression values (FPKMs) were normalized across the entire data set prior to conducting PCA for (a) PC1-2 and (b) PC2-3. Note that each tissue type grouped

together, with the exception of the inflorescence from the *VvCEB1*$_{opt}$-overexpressing line (#26) in PCA1-2.

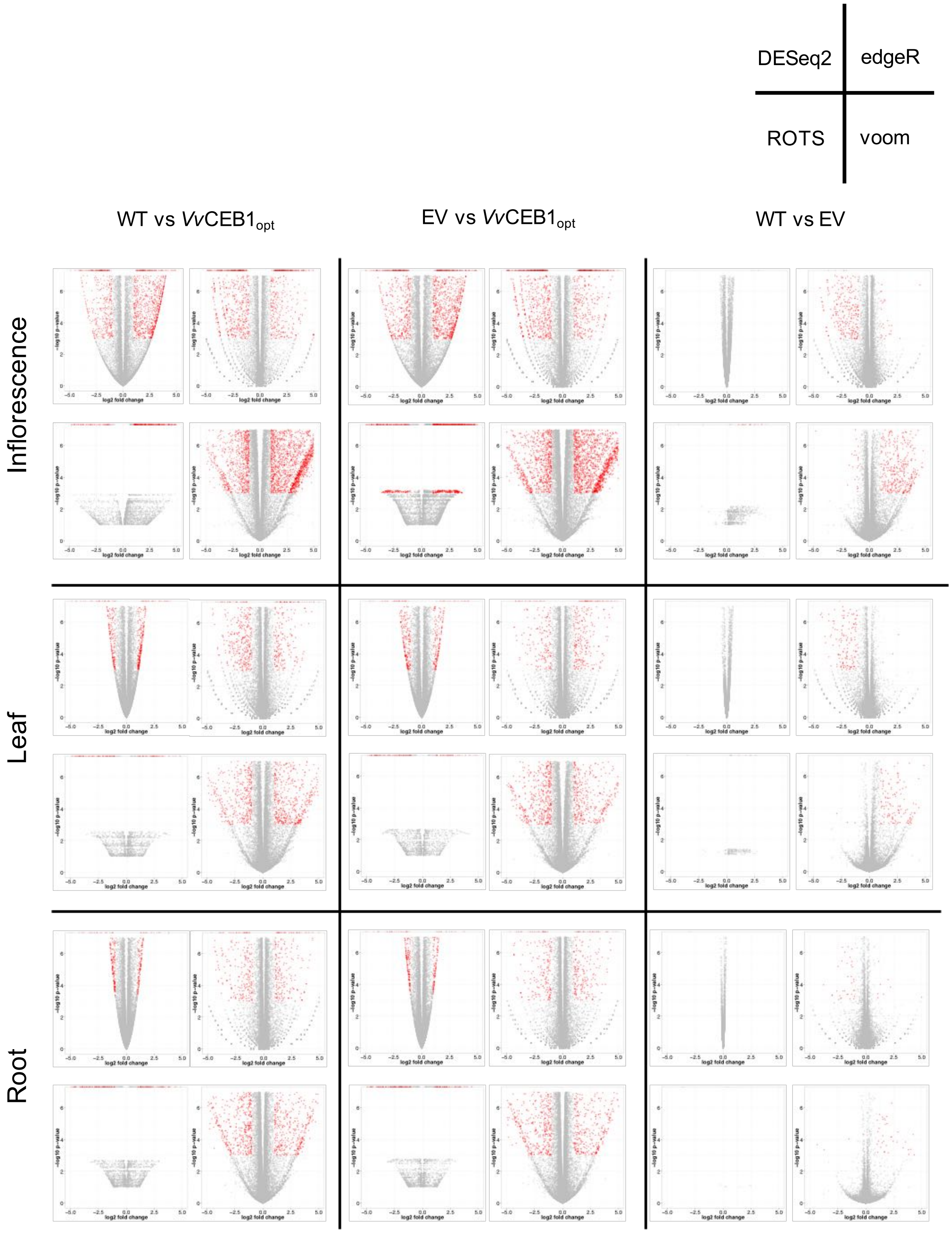

Figure S22. Volcano plots showing differential mRNA expression within various dataset comparisons. Log-fold changes indicated the relative gene expression within each comparison pair. The statistical significance shows negative logarithm. The mRNA expression changes that were statistically significant (p-value ≤ 0.001 and ≥ 2 fold) are

colored in red. The key in top right corner indicates the four different differentially expressed gene (DEG) programs used for each comparison (e.g., DESeq2, edgeR, ROTS, and voom).

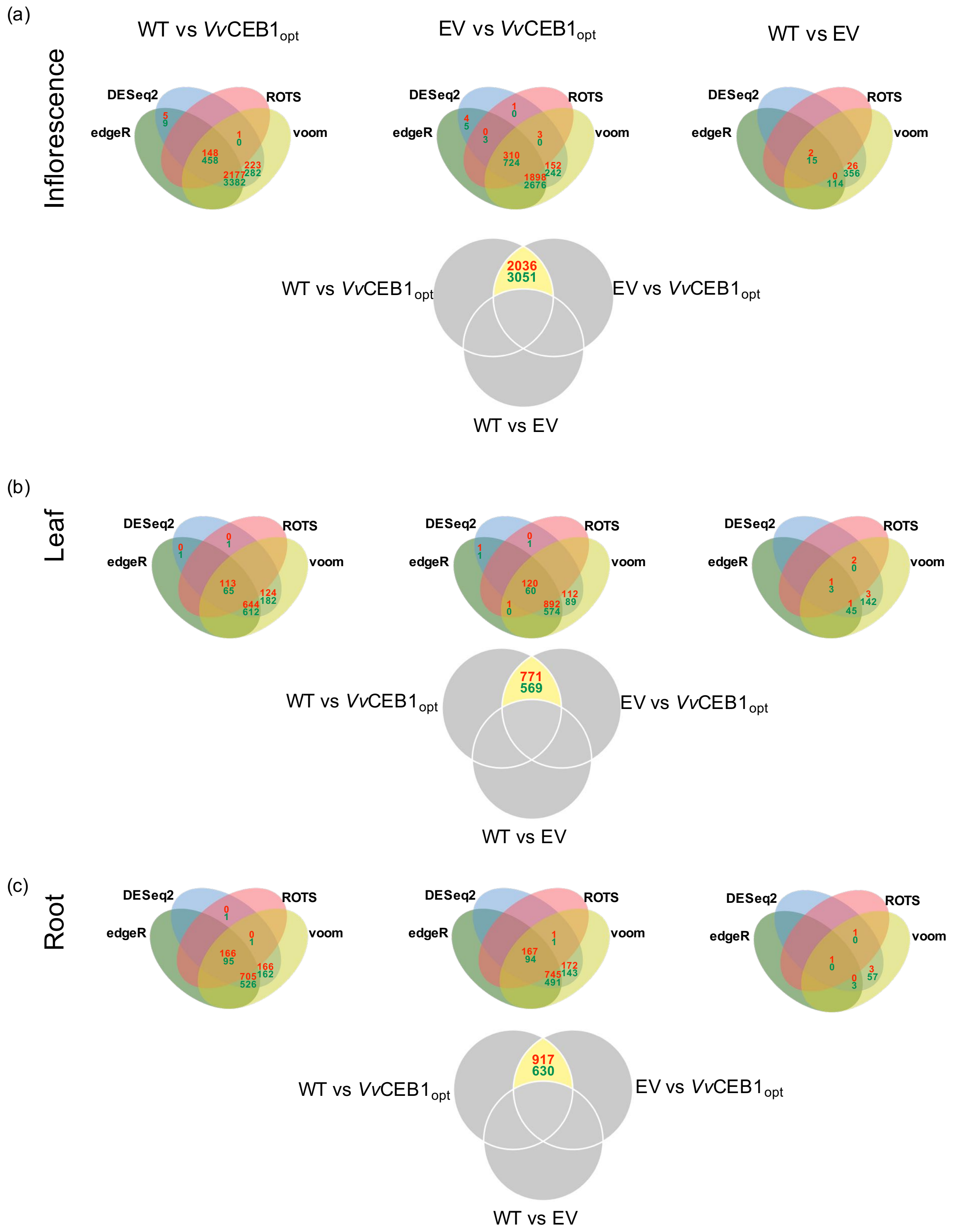

Figure S23. Venn diagrams showing the number of differentially expressed genes within (a) inflorescence, (b) leaf, and (c) root with statistically significant fold-changes in mRNA expression between Col-0 wild type (WT) compared with *VvCEB1$_{opt}$*, *35S::3xHA* empty-vector control (EV) compared with *VvCEB1$_{opt}$*, Col-0 WT compared

with EV, and all possible combinations of the pairs of genotypes. Four-way Venn diagrams were used to show the intersection of the differentially expressed genes identified by more than two tools when comparisons were made using DESeq2, edgeR, ROTS, or voom. Three-way Venn diagrams were used to show the intersection of differentially expressed genes between WT compared with $\textit{VvCEB1}_{opt,}$ and EV compared with $\textit{VvCEB1}_{opt}$, which showed no overlap in gene expression with WT compared with EV.

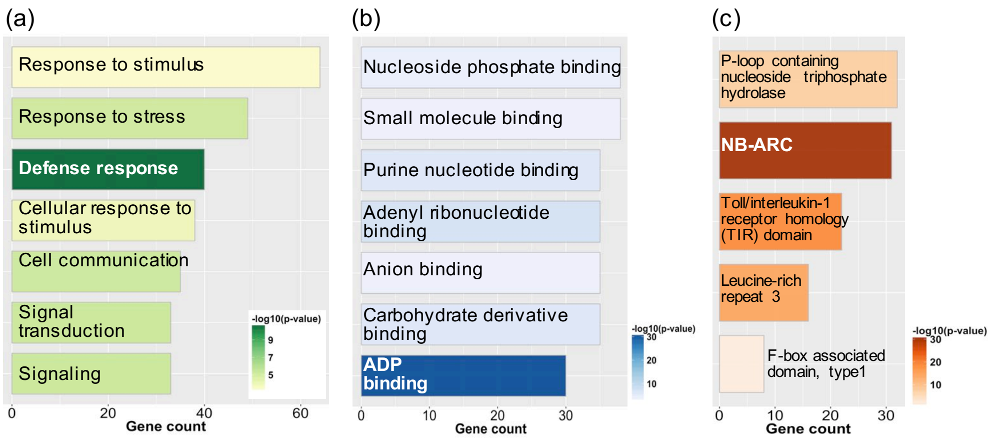

Figure S24. **The consensus set of 227 genes with increased transcript abundance showed enrichment for gene ontology (GO) terms involved in several biological processes.** Analysis using ThaleMine at the Araport database (Krishnakumar et al., 2015) revealed (a) biological process analysis revealed enrichment of genes involved in responses to environmental stress and stimulation, with significant enrichment of genes in cell communication and signaling and especially defense response functions. (b) Molecular function analysis revealed enrichment of genes involved in small molecule binding, anion and carbohydrate derivative binding, and nucleoside binding, with a strong enrichment in ADP binding. (c) Protein domain enrichment analysis revealed enrichment of genes with P-loop nucleoside triphosphate hydrolase activities (NTPase), *NB-ARC* (nucleotide-binding adaptor shared by *APAF-1*, R proteins, and *CED-4*) domains from resistance proteins involved in plant innate immunity, Toll/interleukin-1 receptor homolog (*TIR*) domains of nucleotide binding site-leucine-rich (*LRR*) repeat (*NBS-LRR*) proteins that recognize pathogen-derived effector molecules, leucine-rich repeat domains of disease resistance proteins, and F-box associated domains. Significantly over-represented terms within each database were defined by their hypergeometric distribution after applying the Holm-Bonferroni FDR correction for multiple comparisons with a significance threshold ($p < 0.05$). Bar graph size and color indicates the number of genes associated with enrichment terms and their corrected P values, respectively.

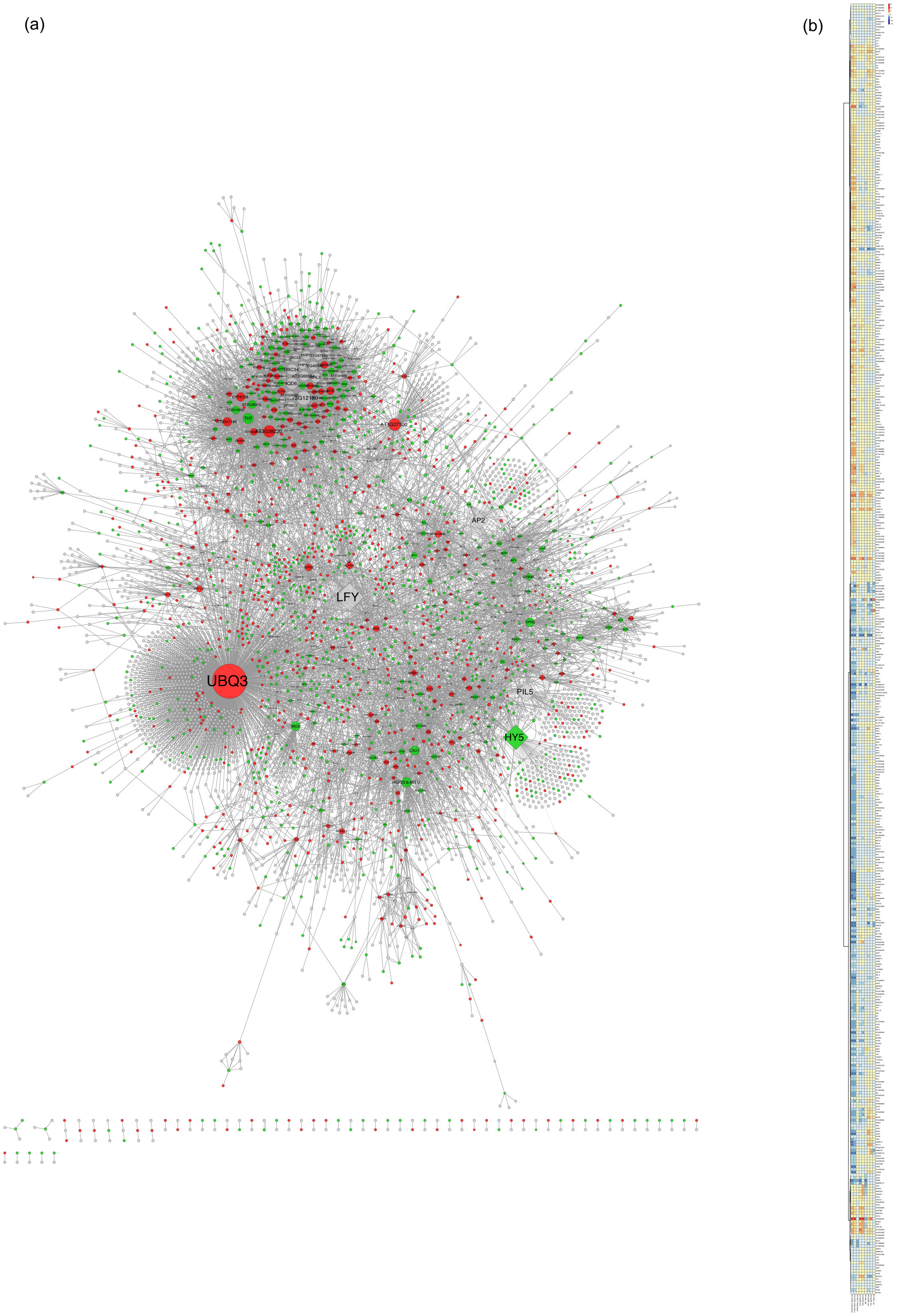

Figure S25. Network analysis of functional associations of differentially expressed genes within inflorescences. (a) Network connecting 5650 genes (776 and 992 genes with increased or decreased relative transcript abundance, respectively) by annotated protein-protein and regulatory interactions. (b) Heat map indicating the relative expression of hub

genes with high connectivity (> 10) based on their topology coefficients (see Figure 6c for more details).

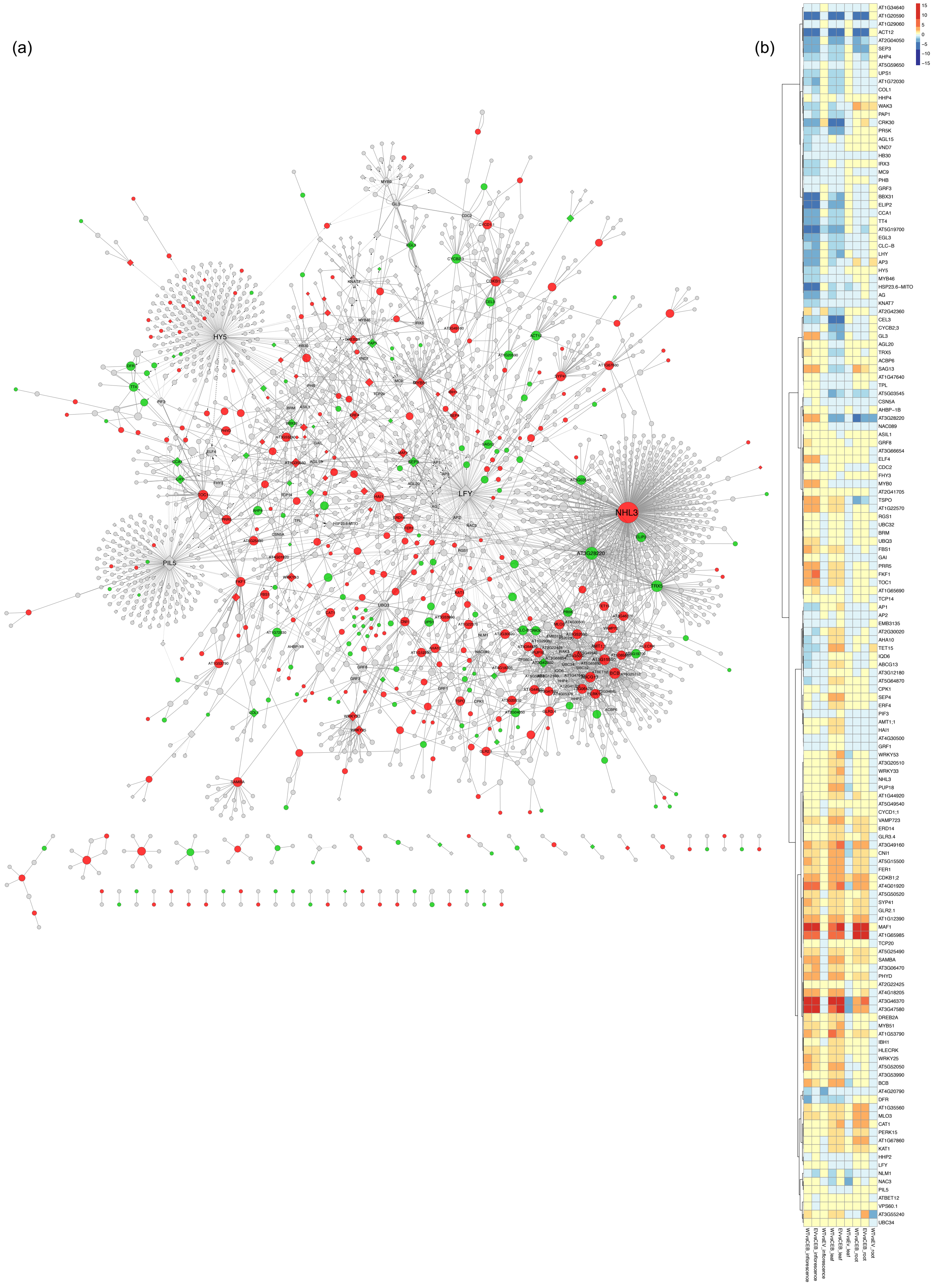

Figure S26. Network analysis of functional associations of differentially expressed genes within leaves. (a) Network connecting 2189 genes (261 and 149 genes with

increased or decreased relative transcript abundance, respectively) by annotated protein-protein and regulatory interactions. (b) Heat map indicates the relative expression of hub genes with high connectivity (> 7) based on their topology coefficients (see Figure 6c for more details).

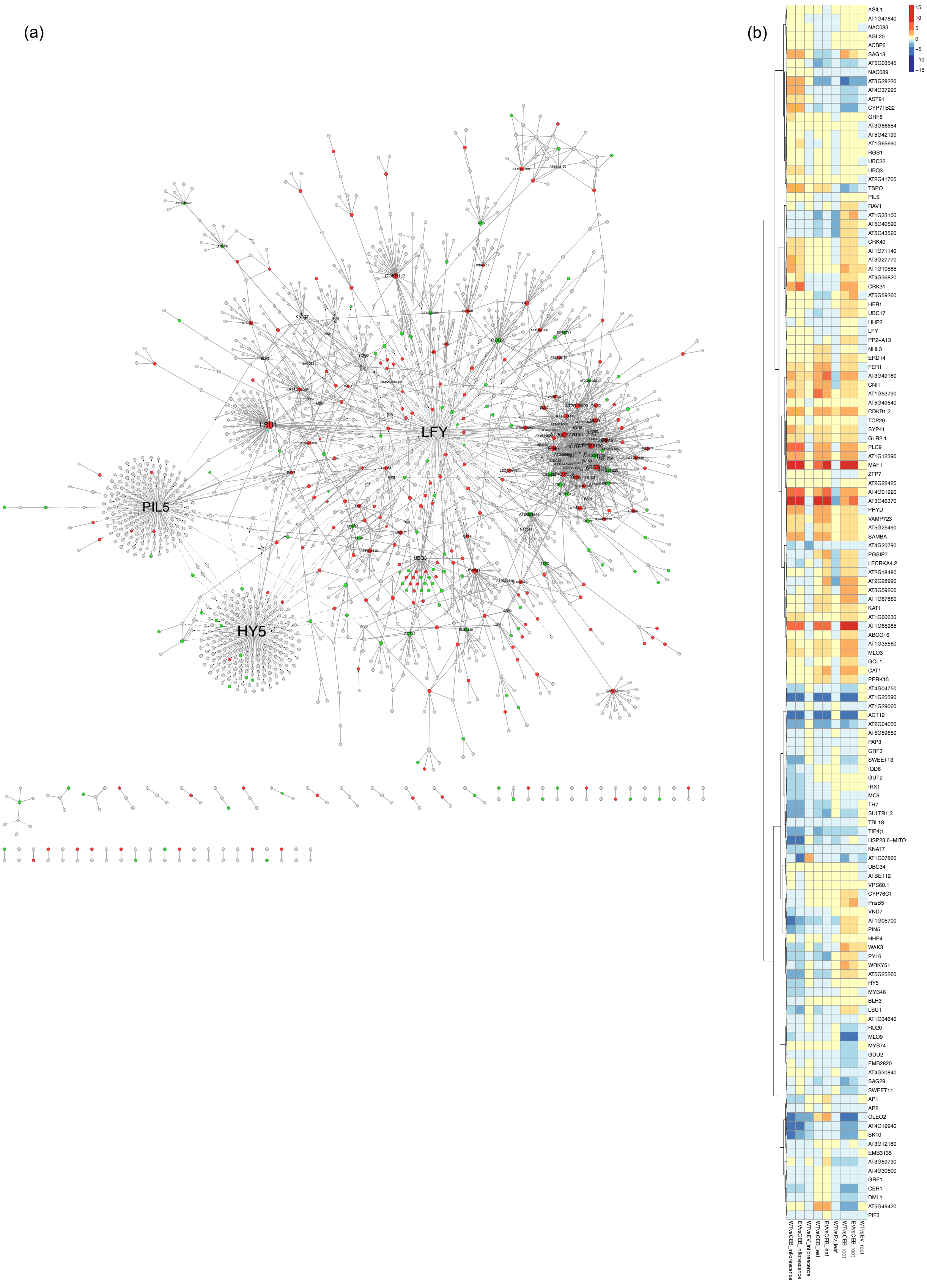
(a)
PIL5
HY5
LFY
LSU1
(b)
15
10
5
0
−5
−10
−15
ASIL1
AT1G47640
NAC083
AGL20
ACBP6
SAG13
AT5G03545
NAC089
AT3G28220
AT4G37220
AST91
CYP71B22
GRF8
AT3G66654
AT5G42190
AT1G65690
RGS1
UBC32
UBQ3
AT2G41705
TSPO
PIL5
RAV1
AT1G33100
AT5G40590
AT5G43520
CRK40
AT1G71140
AT3G27770
AT1G10585
AT4G36820
CRK31
AT5G59260
HFR1
UBC17
HHP2
LFY
PP2−A13
NHL3
ERD14
FER1
AT3G49160
CNI1
AT1G53790
AT5G49540
CDKB1;2
TCP20
SYP41
GLR2.1
PLC9
AT1G12390
MAF1
ZFP7
AT2G22425
AT4G01920
AT3G46370
PHYD
VAMP723
AT5G25490
SAMBA
AT4G20790
PGSIP7
LECRKA4.2
AT2G18480
AT2G28990
AT3G59200
AT1G67860
KAT1
AT1G60630
AT1G65985
ABCG16
AT1G35560
MLO3
GCL1
CAT1
PERK15
AT4G04750
AT1G20590
AT1G29060
ACT12
AT2G04050
AT5G59650
PAP3
GRF3
SWEET13
IQD6
GUT2
IRX1
MC9
TH7
SULTR1;3
TBL18
TIP4;1
HSP23.6−MITO
KNAT7
AT1G07860
UBC34
ATBET12
VPS60.1
CYP76C1
PnsB5
VND7
AT1G05700
PIN5
HHP4
WAK3
PYL6
WRKY51
AT5G25260
HY5
MYB46
BLH3
LSU1
AT1G34640
RD20
MLO9
MYB74
GDU2
EMB2820
AT4G30840
SAG29
SWEET11
AP1
AP2
OLEO2
AT4G19940
SK10
AT3G12180
EMB3135
AT3G59730
AT4G30500
GRF1
CER1
DML1
AT5G49420
PIF3
WTvsCEB_inflorescence
EVvsCEB_inflorescence
WTvsEV_inflorescence
WTvsCEB_leaf
EVvsCEB_leaf
WTvsEV_leaf
WTvsCEB_root
EVvsCEB_root
WTvsEV_root

Figure S27. Network analysis of functional associations of differentially expressed genes within roots. (a) Network connecting 1794 genes (36 and 20 genes with increased or decreased relative transcript abundance, respectively) by annotated protein-protein and regulatory interactions. (b) Heat map indicates the relative expression of hub genes with high connectivity (> 7) based on their topology coefficients (see Figure 6c for more details).
